# A nanobody toolbox targeting dimeric coiled-coil modules for functionalization of designed protein origami structures

**DOI:** 10.1073/pnas.2021899118

**Published:** 2021-04-23

**Authors:** Andreja Majerle, San Hadži, Jana Aupič, Tadej Satler, Fabio Lapenta, Žiga Strmšek, Jurij Lah, Remy Loris, Roman Jerala

**Affiliations:** ^a^Department of Synthetic Biology and Immunology, National Institute of Chemistry, SI-1000 Ljubljana, Slovenia;; ^b^Department of Physical Chemistry, Faculty of Chemistry and Chemical Technology, University of Ljubljana, SI-1000 Ljubljana, Slovenia;; ^c^Graduate Study Program, Faculty of Chemistry and Chemical Technology, University of Ljubljana, SI-1000 Ljubljana, Slovenia;; ^d^Vlaams Instituut voor Biotechnologie, Vrije Universiteit Brussel Center for Structural Biology, Vrije University Brussels, B-1050 Brussels, Belgium

**Keywords:** nanobody, coiled-coil dimer, coiled-coil protein origami, protein assembly, protein design

## Abstract

Coiled-coil (CC) dimers are widely used in protein design because of their modularity and well-understood sequence–structure relationship. In CC protein origami design, a polypeptide chain is assembled from a defined sequence of CC building segments that determine the self-assembly of protein cages into polyhedral shapes, such as the tetrahedron, triangular prism, or four-sided pyramid. However, a targeted functionalization of the CC modules could significantly expand the versatility of protein origami scaffolds. Here, we describe a panel of single-chain camelid antibodies (nanobodies) directed against different CC modules of a de novo designed protein origami tetrahedron. We show that these nanobodies are able to recognize the same CC modules in different polyhedral contexts, such as isolated CC dimers, tetrahedra, triangular prisms, or trigonal bipyramids, thereby extending the ability to functionalize polyhedra with nanobodies in a desired stoichiometry. Crystal structures of five nanobody-CC complexes in combination with small-angle X-ray scattering show binding interactions between nanobodies and CC dimers forming the edges of a tetrahedron with the nanobody entering the tetrahedral cavity. Furthermore, we identified a pair of allosteric nanobodies in which the binding to the distant epitopes on the antiparallel homodimeric APH CC is coupled via a strong positive cooperativity. A toolbox of well-characterized nanobodies specific for CC modules provides a unique tool to target defined sites in the designed protein structures, thus opening numerous opportunities for the functionalization of CC protein origami polyhedra or CC-based bionanomaterials.

## Significance

Coiled-coil dimers are structural motifs which occur frequently in natural proteins. They consist of two α-helices which wind around each other to form a supercoil and can be used as versatile modules for the construction of designed proteins. Here, nanobodies generated against the coiled-coil protein origami tetrahedral cage exhibit binding of one to four nanobodies per coiled-coil dimer in the tetrahedron and also in other types of protein origami structures. High-resolution structures of nanobodies bound to coiled-coil dimers revealed the range of binding motifs, including a positive allosteric binding to the tetrahedron and to the isolated coiled-coil dimeric module, introducing nanobodies as building modules in the protein assembly design.

The coiled-coil (CC) structural motif is one of the most widespread structural elements found in proteins and among the best understood motifs in terms of sequence–structure relationships. It is composed of two or more α-helices in parallel or antiparallel orientation that wind around each other to form a superhelix. The canonical dimeric CC is a twisted left-handed supercoiled structure characterized by heptad repeats (seven amino acid residues labeled *abcdefg*). The elongated shape of the CCs, their periodicity, rigidity, autostabilization, a well-understood principle governing the pairing specificity of the CCs (1, 2), and the ability to control their oligomerization state (3) make them very suitable elements for the de novo design of protein assemblies, such as fibers (4), cages (5), and nanotubes (6). Different sets of orthogonal dimeric CCs have been used to design nanostructures with triangular (7) or rectangular shapes (8), highlighting the importance of this type of modules for protein design.

Precise pairing specificity of CCs in many ways resembles that of the DNA duplex (9). While the structure of the DNA duplex is determined by the complementarity of the base pairs, the pairing specificity of CCs is determined by a combination of hydrophobic and electrostatic interactions between residues at positions *a*, *d*, *e*, and *g* of the heptad repeat. Orthogonal CC dimers have been used to translate the concept of pairwise complementarity of nucleic acid modules (10) into de novo designed three-dimensional (3D) protein nanostructures consisting of a single polypeptide chain that self-assembles into a designed shape with dimeric CCs that form edges. This principle underlies the design of CC protein origami cages, which can adopt polyhedral cage-like structures (11, 12). According to this approach, peptide segments which are orthogonal pairwise-interacting building modules are arranged in a precise sequential order, defining the path of the polypeptide chain to form edges of a stable polyhedral protein cage (13). As in the case of DNA origami (14), the designed structure is defined by the long-range interactions between orthogonal CC segments that direct the final self-assembly; however, the DNA duplex modules are replaced by the dimeric CC modules. In this type of a protein fold, the structure is defined by the topology of the chain of interacting modules rather than by the compact hydrophobic core as in natural proteins (13, 15). The topology of the chain segments can define a large variability of different 3D folds. These are robust, as any CC pair can be exchanged with a different orthogonal pair while maintaining the same polyhedral shape. This strategy was first demonstrated by the design of a single-chain polypeptide tetrahedral fold and later by the design of cages with increasing complexity and size, such as the triangular prism and four-sided pyramid (11, 12).

Although the introduction of amino acid residues at selected positions can be used to functionalize the designed CC protein origami, such as the introduction of metal binding sites (16) or chemically reactive cysteine groups, a targeted binding of CCs using protein domains would be an important addition to the functionalization of designed protein assemblies. Selection of protein domains that specifically bind only to the desired polyhedral edges would represent a modular and exchangeable approach. We reasoned that this could be achieved using single-variable domain heavy-chain only antibodies or nanobodies, which are camelid immunoglobulins that have been minimized to contain only the variable domain. Nanobodies possess the full antigen-binding specificity of the parental antibody and have gained recognition as an alternative to conventional antibodies (17). They usually have an exposed convex paratope that allows them to bind to protein cavities. Nanobodies usually target globular proteins but also recognize linear epitopes (18, 19).

Here, we present and characterize a panel of nanobodies that can be used to functionalize designed proteins built using CC dimers such as protein origami. The nanobodies were selected to bind to different CC modules representing the edges of the designed tetrahedral protein. However, these nanobodies also specifically recognize CC modules in different polyhedral designs practically regardless of the context in which they are positioned within the protein cages. The crystal structures of five complexes consisting of CC dimers and nanobodies show that the nanobodies bind primarily to the noninteracting sites of the CC dimers and, in addition to complementarity determining region 3 (CDR) loops, strongly rely on the nanobody framework residues for binding. The presented crystal structures, including new high-resolution structures of the designed CCs APH_2_ and P5-P6, suggest strategies for rational protein assembly design, since CC are frequently used in this field of synthetic biology.

## Results

### Nanobodies Target Different CC Modules in the Protein Origami Tetrahedron TET12SN.

**Fig. 1.**
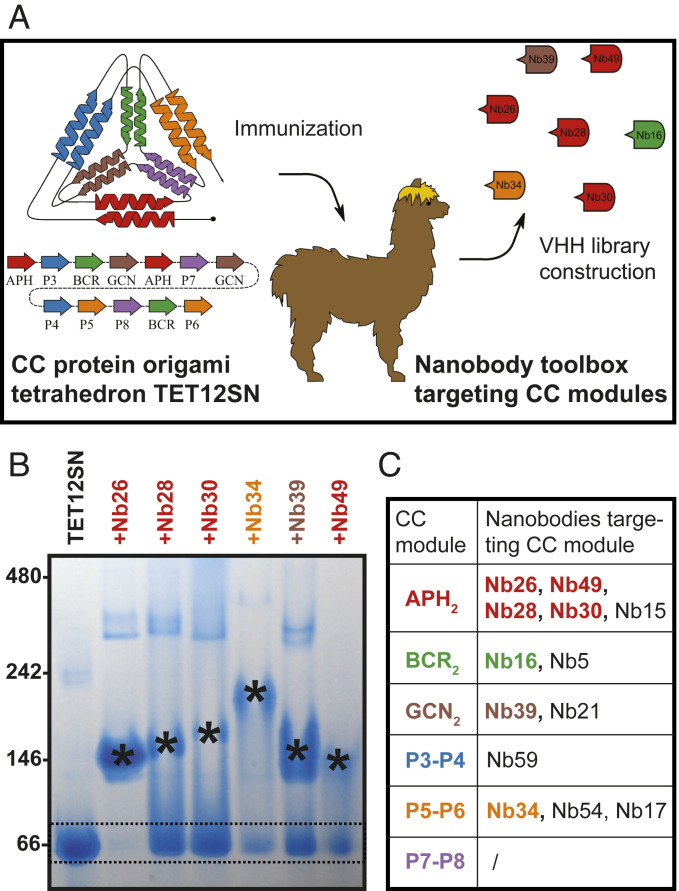
Generated nanobodies bind to CC modules of the protein origami tetrahedron TET12SN. (*A*) TET12SN consists of two antiparallel (APH_2_ and BCR_2_) and four parallel CC modules (GCN_2_, P3-P4, P5-P6, and P7-P8) and was used to immunize a single llama to generate a library of variable domains of camelid heavy-chain only antibody sequences. Nanobodies specific for TET12SN were selected using phage display, produced in *E. coli*, and characterized further. (*B*) Identification of individual nanobodies that bind to the target protein TET12SN by native PAGE (presented are the results for nanobodies Nb26, Nb28, Nb30, Nb34, Nb39, and Nb49, which were characterized in crystal structures). TET12SN (5 µM) was incubated overnight with nanobodies in 5- or 10-fold molar excess. The sizes of the proteins (in kDa) in the protein standard are marked on the left side. The position of TET12SN (53.4 kDa, pI = 4.70) is marked by two dashed lines, while different nanobody-TET12SN complexes are marked with asterisks. The data are representative of two independent experiments. (*C*) Summary of the nanobody panel targeting TETSN12. The characterized nanobodies Nb26, Nb28, Nb30, Nb49, Nb16, Nb39, and Nb34 are highlighted with the same color as the targeted CC module. Additional native PAGE gels showing nanobody binding are presented in *SI Appendix*, Fig. S2.

The most extensively characterized protein origami cage, tetrahedron TET12SN (12), self-assembles from a single 461 amino acid residue polypeptide chain consisting of 12 CC dimer-forming modules adopting antiparallel or parallel orientations (APH_2_, BCR_2_, GCN_2_, P3-P4, P5-P6, and P7-P8). These CC modules form edges of the tetrahedron and are linked by flexible peptides that coincide at the vertices. TET12SN was used to immunize a llama to generate a library that was panned for binders using a phage display (20) (Fig. 1*A*). From this library, 29 unique nanobody sequences were obtained, which were classified into 14 nanobody groups based on the sequence comparison of the CDR3.

We produced recombinant nanobodies in *Escherichia coli* (*SI Appendix*, Fig. S1) and identified their binding epitopes on TET12SN using native polyacrylamide gel electrophoresis (PAGE). Nanobody-TET12SN complexes exhibit reduced electrophoretic mobility compared with free TET12SN (53 kDa), indicating that nanobodies bind TET12SN (Fig. 1*B* and *SI Appendix*, Fig. S2 *A* and *B*). Furthermore, addition of different synthetic peptides forming CCs corresponding to different edges of the TET12SN tetrahedron led to the dissociation of nanobody-TET12SN complexes, identifying peptide pairs that compete with TET12SN in nanobody binding (*SI Appendix*, Fig. S2 *C* and *D*). This enabled identification of binding epitopes for the majority of nanobody groups and revealed that five of six CC modules representing edges of TET12SN are targeted by nanobodies (Fig. 1*C*). We set out to elucidate structural aspects of nanobody recognition for several nanobody complexes with CC dimer peptides (sequence alignment of Nb26, Nb28, Nb30, and Nb49 targeting APH_2_, Nb39 targeting GCN_2_, and Nb34 targeting P5-P6 module is shown in *SI Appendix*, Fig. S3). Assuming that the nanobodies bind the protein origami cages in the same orientation as to the CC, peptides would enable precise positioning of nanobody binding sites on the cages or other types of CC-based structures.

### Recognition of the Antiparallel Homodimeric APH CC by Nb26 and Nb49.

**Fig. 2.**
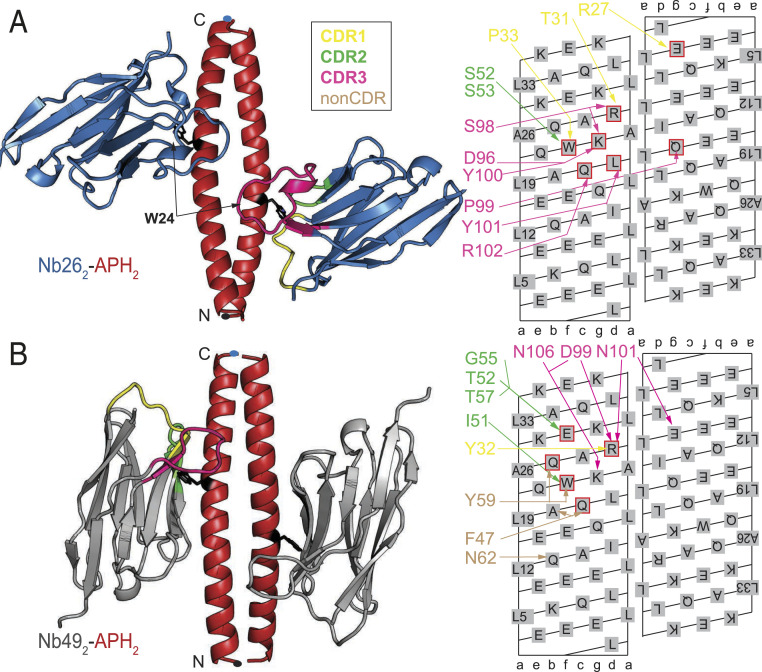
Recognition of the antiparallel APH homodimeric CC by nanobodies Nb26 and Nb49. (*A*) Nb26 (blue) binds to the APH_2_ (red) epitope centered around W24_*f*_ residue (represented in black sticks) using three CDR loops. The APH N and C termini are marked with dots. Nanobody interactions with APH_2_ are shown schematically on the CC surface lattice with different colors corresponding to the CDR loops. APH residues mediating hotspot interactions are highlighted by the red squares. (*B*) In the structure of the Nb49_2_-APH_2_ complex, the Nb49 (gray) is positioned parallel to the axis of APH_2_ (red), which allows additional interactions with the nanobody framework residues (non-CDR interactions). Interactions mediated by non-CDR residues are shown in brown.

**Fig. 3.**
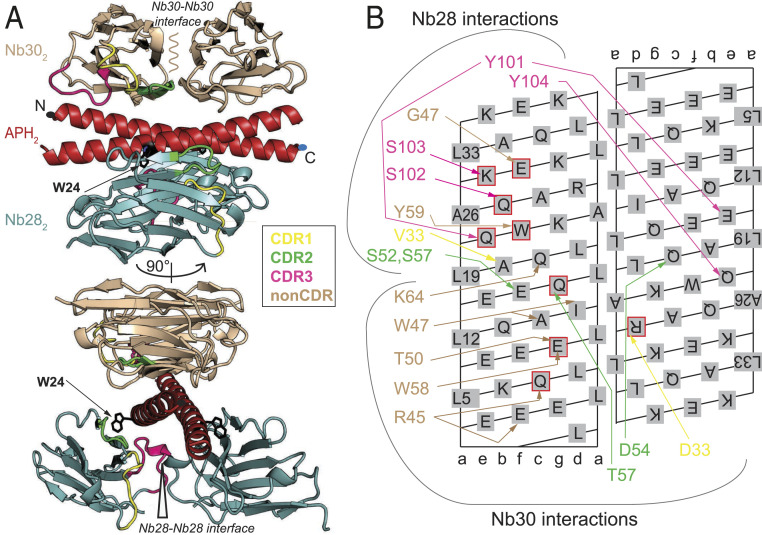
Structure of the ternary Nb28_2_-Nb30_2_-APH_2_ complex. (*A*) The crystal structure shows that Nb28 (teal) and Nb30 (light brown) bound to the APH_2_ dimer (red) at the opposite interfaces. The Nb28 epitope is centered around W24_*f*_ (represented in black sticks) and the C-terminal part of APH. The Nb30 forms extensive interactions using framework residues (non-CDR) mainly with the N-terminal part of APH. The N and C termini of the APH chain are marked with dots. (*B*) Nanobody interactions with the APH_2_ are shown schematically on the CC surface lattice with different colors corresponding to the CDR loops, while non-CDR interactions are shown in brown. APH residues mediating hotspot interactions are highlighted by the red squares.

A substantial fraction of nanobodies (five groups) recognized the designed antiparallel homodimeric APH CC (21). This may be due to the presence of a Trp residue at the exposed position *f* since the nanobodies preferentially recognize epitopes enriched with the aromatic residues (22). The APH sequence used in our study differs from the original APH sequence by having Glu residues at position *f*, as this increased the solubility of the designed tetrahedron (12). Since APH CC (APH_2_) is antiparallel, it presents an identical binding surface on both chains, resulting in a 2:2 (nanobody:CC peptide) stoichiometry of all nanobody-APH complexes (Figs. 2 and 3).

The interaction between Nb26 and APH_2_ involves all three CDR loops and buries about 1,500 Å^2^ of solvent-accessible surface area per nanobody molecule (Fig. 2*A*; crystal data collection and refinement statistics are listed in *SI Appendix*, Table S1). The CDR2 loop interacts only with one APH chain, while CDR1 and CDR3 extend over the first chain and create numerous interactions with the second APH chain. The APH interface is centered on W24 at position *f* (W24_*f*_), which is partially shielded from the solvent by CDR1 and CDR2 loops (*SI Appendix*, Fig. S4*A*). Furthermore, the residues of CDR1 form two salt bridges with the APH residues R29_*d*_ and E4_*g*’_ (CC positions on the second chain are marked with an apostrophe: *a*’ to *g*’). However, most interactions are mediated by the long CDR3 loop, which forms main-chain interactions with Q21_*c*_ and K25_*g*_. The tip of the CDR3 loop (sequence PYY) shields the hydrophobic residues in the core of CC dimer (*SI Appendix*, Fig. S4*B*). Using computational alanine scanning, we identified that energetically most important interactions (hotspots) are mediated by the APH residues W24_*f*_ and K25_*g*_ as well as Q21_*c*_ and L22_*d*_ (Fig. 2*A*). Slightly weaker interactions are mediated by the residues R29_*d*_ and I15_*d*’_ and L8_*d*’_ and Q18_*g*’_ on the second APH chain. Thermodynamic parameters for the binding of Nb26 to APH_2_ were obtained using isothermal titration calorimetry (ITC) and show that the binding affinity is very high (*K*_d_ = 39 nM), mainly due to the favorable enthalpic contribution (Δ*H* = −28 kcal ⋅ mol^−1^) (*SI Appendix*, Fig. S5*A*; all thermodynamic parameters are listed in *SI Appendix*, Table S2). When the APH segment is placed in the context of TET12SN, the Nb26-TET12SN affinity is higher compared with APH_2_ alone, *K*_d_ = 1.8 nM (*SI Appendix*, Fig. S6*A*), suggesting that APH_2_ may be more rigid in the context of the cage compared with the isolated peptide dimer.

The crystal structure of the Nb49_2_-APH_2_ complex shows a completely different binding mode in which the β-plane of the nanobody is aligned parallel to the APH_2_ axis (Fig. 2*B*). This results in a rather extended hydrophobic interface, where the aromatic residues from the β-strands C, C’, and C” completely bury the W24_*f*_ side chain (the nomenclature of the nanobody strands is based on ref. 23) (*SI Appendix*, Fig. S4*C*). In the Nb49_2_-APH_2_ complex, the hotspot interactions are mediated by the APH residue R29_*d*_, which is involved in a salt bridge with the residues from the CDR3 loop and a cation-pi interaction with the Tyr from the CDR1 loop (*SI Appendix*, Fig. S4*D*). In contrast to the epitope of Nb26 which extends over both APH chains, each of the Nb49 interacts almost exclusively with a single APH chain. ITC titrations showed that the affinity of Nb49_2_-APH_2_ is *K*_d_ = 570 nM and therefore weaker than that of Nb26 (*SI Appendix*, Fig. S5*B*). Interestingly, also in the case of Nb49, higher affinity is observed for TET12SN (*K*_d_ = 160 nM) than for the APH peptide (*SI Appendix*, Fig. S6*B*).

Investigation of both crystal structures shows the importance of residue W24_*f*_ in the APH_2_ dimer for nanobody binding. We investigated whether nanobody binding stoichiometry in the TET12SN tetrahedron can be modified from 2:1 to 1:1 or 0:1 (nanobody:TET12SN) by point mutations of W24_*f*_ in either of the APH segments. We prepared three TET12SN variants, TET12SN(W24A)_1_, TET12SN(W24A)_5_, and TET12SN(W24A)_1,5_, where a point mutation is made in first, second, or both APH segments. Amino acid substitutions did not perturb the protein secondary structure or their thermal stability compared with the original TET12SN (*SI Appendix*, Fig. S7), which is to be expected since Trp is located at position *f* distant from the CC dimer interface. Nb26 binds to TET12SN(W24A)_1_ and TET12SN(W24A)_5_ variants with the inactivated single binding epitope, but different migrations on native PAGE of these complexes relative to the original TET12SN suggest that only a single Nb26 molecule binds to these two variants (*SI Appendix*, Fig. S8 *A*–*D*). Similarly, the reduced size of the Nb49 complex with TET12SN(W24A)_1_ or TET12SN(W24A)_5_ indicates that only a single Nb49 nanobody molecule binds to the tetrahedral molecule with one inactivated binding epitope (*SI Appendix*, Fig. S8). The difference in the positions of these two complexes in the native gel likely indicates differences in the orientation of a bound single Nb49 molecule versus the tetrahedral scaffold. For a variant with inactivated both binding epitopes (TET12SN(W24A)_1,5_), no binding of nanobodies targeting APH_2_ module was observed, although binding of nanobodies targeting the GCN_2_ or P5-P6 module was maintained, demonstrating that this modification did not disrupt the fold of the cage (*SI Appendix*, Fig. S8*D*). This demonstrates that we can tune the stoichiometry of nanobody binding by single-point mutations that do not affect the tetrahedral fold.

### Structure of the Ternary Nb28_2_-Nb30_2_-APH_2_ Complex.

In contrast to other binders of APH_2_, the combination Nb28 and Nb30 is able to bind APH_2_ concomitantly (*SI Appendix*, Fig. S9), indicating that their binding epitopes do not overlap. The crystal structure shows that Nb28 and Nb30 bind to the opposite sides of APH_2_ and form a ternary Nb28_2_-Nb30_2_-APH_2_ complex (Fig. 3*A*). Interestingly, in this complex, there are no direct interactions between Nb28 and Nb30 molecules. The orientation of Nb28 is parallel to the APH_2_ axis, similar to that observed for the complex with Nb49. This creates a large interaction surface mediated by non-CDR residues burying the W24_*f*_ side chain (*SI Appendix*, Fig. S10*A*). Long CDR3 forms favorable interactions with residues at positions *e* and *f*, while it also extends over the second APH chain to shield the hydrophobic core of CC (residues at position *a*) (*SI Appendix*, Fig. S10*B*). In addition to the Nb28_2_-APH_2_ interface, the complex is stabilized by an additional interface between two Nb28 molecules, which buries about 650 Å^2^ of solvent-accessible surface and is stabilized by interactions between CDR1 from the first and CDR3 from the second Nb28 molecule.

Nb30 recognizes a unique APH_2_ epitope which does not involve the W24_*f*_ side chain (Fig. 3*B*). Two Nb30 molecules bind antiparallel to each other and perpendicular to the axis of APH_2_. The main feature of the Nb30-APH_2_ interface is an extensive use of non-CDR residues (e.g., W47 on strand C’ and W58 on strand C”) that shield the hydrophobic residues at positions *d* (e.g., I15_*d*_) in the APH_2_ core (*SI Appendix*, Fig. S10*C*). Strikingly, interactions via residues on CDR loops are almost absent; perhaps the only such interaction is the salt bridge between D33 on CDR1 and positively charged residues on the other APH chain (*SI Appendix*, Fig. S10*D*). In addition to the Nb30-APH_2_ interface, an extensive interface is formed between two Nb30 molecules, burying about 650 Å^2^ of the solvent-accessible area. This interface is stabilized by interactions between CDR2 and the C”D loop. Thus, the ternary structure of the Nb28_2_-Nb30_2_-APH_2_ complex is additionally stabilized by the Nb30-Nb30 and Nb28-Nb28 interfaces.

### The Allosteric Coupling between Nb28 and Nb30 Enhances Binding to APH CC.

**Fig. 4.**
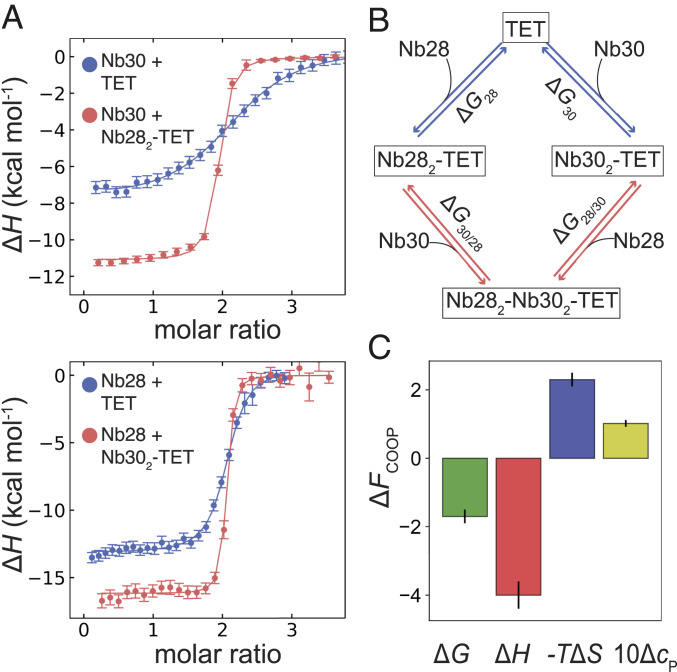
Allosteric recognition of the APH_2_ module in the tetrahedron TET12SN. (*A*) ITC titrations show that the binding affinity is increased more than 10-fold when Nb28 binds the Nb30_2_-TET12SN complex (red isotherm) compared with its binding to TET12SN alone (blue isotherm). A similar positive coupling is also observed for titrations for Nb30 binding to TET12SN alone (blue isotherm) or to the Nb28_2_-TET12SN complex (red isotherm) as shown on lower panel. (*B*) Thermodynamic cycle for formation of ternary complex Nb28_2_-Nb30_2_-TET12SN by two possible routes, where either Nb28 or Nb30 binds first. Interaction free energies (Δ*G*_28_, Δ*G*_30_, Δ*G*_28/30_, Δ*G*_30/28_) describe the formation of the various complexes. The coupling free energy can be calculated along either path of the cycle as Δ*G*_COOP_ = Δ*G*_30_ − Δ*G*_30/28_ = Δ*G*_28_ − Δ*G*_28/30_. (*C*) Thermodynamics of the allosteric coupling between Nb28 and Nb30 binding to tetrahedron TET12SN. The positive cooperativity contribution is driven by a favorable enthalpic coupling. Errors were estimated from the difference between the Δ*F*_COOP_ parameters obtained for each path leading to the ternary complex.

Nb30 alone binds relatively poorly to the TET12SN (*K*_d_ = 350 nM) (*SI Appendix*, Fig. S6*D*), but affinity increases by more than 10-fold (*K*_d_ = 26 nM) when Nb30 is titrated into the preformed Nb28_2_-TET12SN complex (Fig. 4 *A*, *Upper*). Equivalently strong positive cooperativity is observed when the ternary Nb28_2_-Nb30_2_-TET12SN complex is formed in a different order; when Nb28 is titrated into the preformed Nb30_2_-TET12SN complex (Fig. 4 *A*, *Lower*), the affinity increases from *K*_d_ = 39 nM (*SI Appendix*, Fig. S6*C*) to 2 nM.

To investigate whether positive cooperativity stems from the APH_2_ module alone or from the surrounding tetrahedron cage, we preformed titrations using the APH_2_ peptide. In this case too, we observed that Nb28 and Nb30 binding is coupled by positive cooperativity, although slightly weaker compared with that observed in the tetrahedron TET12SN (*SI Appendix*, Figs. S5 *C* and *D* and S11). Given that APH_2_ alone is sufficient to observe the cooperative effect and since no direct interactions between Nb28 and Nb30 are observed in the structure of the ternary complex (Fig. 3), the positive cooperativity appears to be mediated allosterically through the conformation or dynamics of the APH_2_ dimer. We next investigated whether the allosteric coupling between Nb28 and Nb30 persists when the segment APH is embedded in another protein origami structure. In this case, we used a tetrahedral construct TET12SN(22CC) with a different sequence of CC segments (24) (*SI Appendix*, Table S3) and observed identical positive coupling as with TETSN12 (*SI Appendix*, Fig. S12). This indicates that allosteric coupling is independent of the topological context of a tetrahedron and is mediated by the APH_2_ alone.

To investigate the origin of the cooperativity in formation of the ternary complex, we used the thermodynamic cycle shown in Fig. 4*B* and determined the coupling parameters Δ*F*_COOP_ (*F* = *G*, *H*, *S*, c_P_). The interaction of Nb28 and Nb30 with TET12SN is associated with a net positive cooperativity contribution Δ*G*_COOP_ = −1.7 kcal ⋅ mol^−1^, which is driven by a favorable enthalpy term Δ*H*_COOP_ = −4 kcal ⋅ mol^−1^ and is countered by the entropic penalty −*T*Δ*S*_COOP_ = +2.4 kcal ⋅ mol^−1 ^(Fig. 4*C*). By performing ITC titrations at different temperatures and using a global model analysis, we determined the heat capacity contributions (Δ*c*_P_) for the binding of Nb28 to APH_2_, to TET12SN, and to Nb30_2_-TET12SN (*SI Appendix*, Fig. S13). It is known that Δ*c*_P_ values correlate with the amount of buried surface area upon complex formation (25). The Δ*c*_P_ contributions for the binding of Nb28 to APH_2_ or TET12SN are very similar, indicating that the interaction surface in Nb28-TET12SN is similar to that observed in the Nb28_2_-Nb30_2_-APH_2_ complex. In other words, the Δ*c*_P_ contributions also suggest that Nb28 does not form additional contacts with TET12SN besides that with APH_2_ and confirms that cooperativity stems mainly from the interactions with the APH_2_ module. Interestingly, the allosteric coupling is accompanied by a positive contribution Δ*c*_P,COOP_ = +100 cal ⋅ mol^−1^ ⋅ K^−1^, indicating that the binding of Nb30 might influence the APH_2_ conformation or its dynamics, which may explain the observed allosteric effect.

### Recognition of the Antiparallel Homodimeric BCR CC.

In the addition to APH_2_, we characterized nanobody binding to another antiparallel homodimeric CC module BCR_2_ (26); however, a crystal structure of a complex could not be obtained. This CC module is a target for nanobodies from two nanobody groups (*SI Appendix*, Fig. S2 *C* and *D*). The binding affinity of nanobody Nb16 to BCR_2_ is high both for the isolated CC and in the context of TET12SN (*K*_d_ = 30 nM and 9.8 nM, respectively) (*SI Appendix*, Figs. S5*E* and S6*E*). Differently to APH_2_, one nanobody binds a CC dimer.

### Recognition of the Parallel Homodimeric GCN CC.

**Fig. 5.**
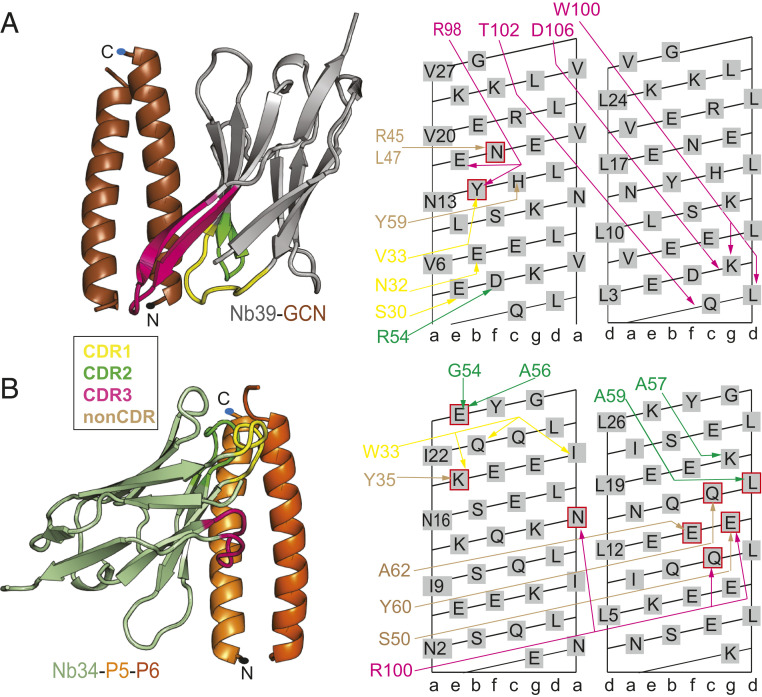
Recognition of the parallel CCs GCN_2_ and P5-P6 by nanobodies Nb39 and Nb34. (*A*) Complex between Nb39 (gray) and GCN_2_ homodimer (brown). Nb39 interacts with both GCN chains using an extended CDR3 loop in a β-hairpin conformation extending from the nanobody framework. Additional contacts with the second GCN chain are provided by CDR1 and framework residues. CC N and C termini are marked with dots. Nanobody interactions (colors correspond to different CDR loops, while non-CDR interactions are shown in brown) are shown schematically on the CC surface lattice, with CC residues mediating hotspot interactions highlighted by red squares. (*B*) Complex between Nb34 (green) and P5-P6 heterodimer (orange). The majority of interactions with P5 (bright orange) and P6 (dark orange) are formed by the CDR2 loop and nanobody framework (non-CDR), with only limited contributions from CDR1 and CDR3 residues. Nanobody interactions with P5-P6 are shown schematically on the CC surface lattice (left lattice shows P5 residues, and the right one shows P6 residues).

The homodimeric GCN CC (GCN_2_) is one of the best characterized parallel homodimeric CCs (27). With respect to the natural GCN4-p1 sequence, the sequence used in this study is five amino acids shorter and differs in one residue at position *b* to increase the solubility (28). Overall, the structure of the GCN_2_ in complex with Nb39 is essentially identical to the structure of the GCN4 leucine zipper domain (29), which shows that the binding of the nanobody does not significantly affect the structure of the peptide CC dimer (*SI Appendix*, Fig. S14). In the crystal structure of the Nb39-GCN_2_ complex, the Nb39 β-sheet plane is oriented parallel to the axis of the GCN_2_, which represents a similar binding mode as observed for Nb49 and Nb28 (Fig. 5*A*). The CDR3 loop adopts a β-hairpin conformation extending from the existing nanobody β-sheet framework. The CDR3 loop shields hydrophobic residues V6_*a*_ and L9_*d*_ in the core of GCN_2_ (*SI Appendix*, Fig. S15*A*). The energetically most important contributions are mediated by CDR3 and include two cation-pi interactions between Y14_*b*_ and the CDR3 arginine as well as K5_*g*’_ side chain and the CDR3 tryptophan side chain. The tip of the CDR3 loop extends to the second GCN chain, which interacts favorably with the residues at positions *d* and *g*. Framework β-strands C’ and C” run parallel to the GCN chain, which positions a number of non-CDR residues to interact with the GCN chain (*SI Appendix*, Fig. S15*B*). The binding affinity of Nb39 to GCN_2_ in the context of the TET12SN tetrahedron is high (*K*_d_ = 27 nM) but lower for the isolated GCN_2_ (*K*_d_ = 850 nM) (*SI Appendix*, Figs. S5*F* and S6*F*). Interestingly, both titrations revealed a second binding site for Nb39, which has much lower affinity (*K*_d_ = 2 µM). Most likely, the second GCN chain may represent a binding site with low affinity for Nb39. However, a different angle of the GCN helix relative to the nanobody β-sheet plane likely disrupts a number of interactions, reducing the overall affinity.

### Recognition of the Parallel Heterodimeric P5-P6 CC.

The P5-P6 is a representative from the designed set of orthogonal parallel heterodimeric CC peptide pairs (30). In the crystal structure of the Nb34-P5-P6 complex, the asymmetric unit contains one molecule of Nb34 in complex with P5-P6 and two additional free Nb34 molecules involved in the crystal contacts. The structure of the P5-P6 peptide dimer agrees with the proposed design (30) and comprises four heptads with an NINI pattern at position *a*, which together with the pattern LLLL at position *d* form the hydrophobic seam of the P5-P6 dimer (Fig. 5*B*). Nb34 interacts with both chains of the P5-P6 dimer, mainly with the residues closer to the C terminus of P5-P6. The CDR2 loop contains a stretch of Ala residues which, together with W33 on CDR1, create hydrophobic contacts with the core residues at positions *a* and *d* (*SI Appendix*, Fig. S15*C*). Additional interactions by CDR1 include a cation-pi interaction with K20_*e*_ and a hydrogen bond with Q23_*b*_. The arginine R100 on the CDR3 loop is involved in a network of interactions with several residues on both P5 and P6 chains (N16_*a*_, Q11_*c*’_, and E15_*g*’_) (*SI Appendix*, Fig. S15*D*). Finally, non-CDR residues on β-strands C’ and C” and the C”D loop contribute to binding (Fig. 5*B*). Compared with other nanobodies, the binding affinity of Nb34, measured for the tetrahedral protein TET12SN, is rather weak, *K*_d_ = 21 µM (*SI Appendix*, Fig. S6*G*), while the affinity for the isolated P5-P6 peptide is likely even lower and could not be reliably determined using ITC.

### Structural Analysis of Nanobody-TET12SN Tetrahedron Complexes.

**Fig. 6.**
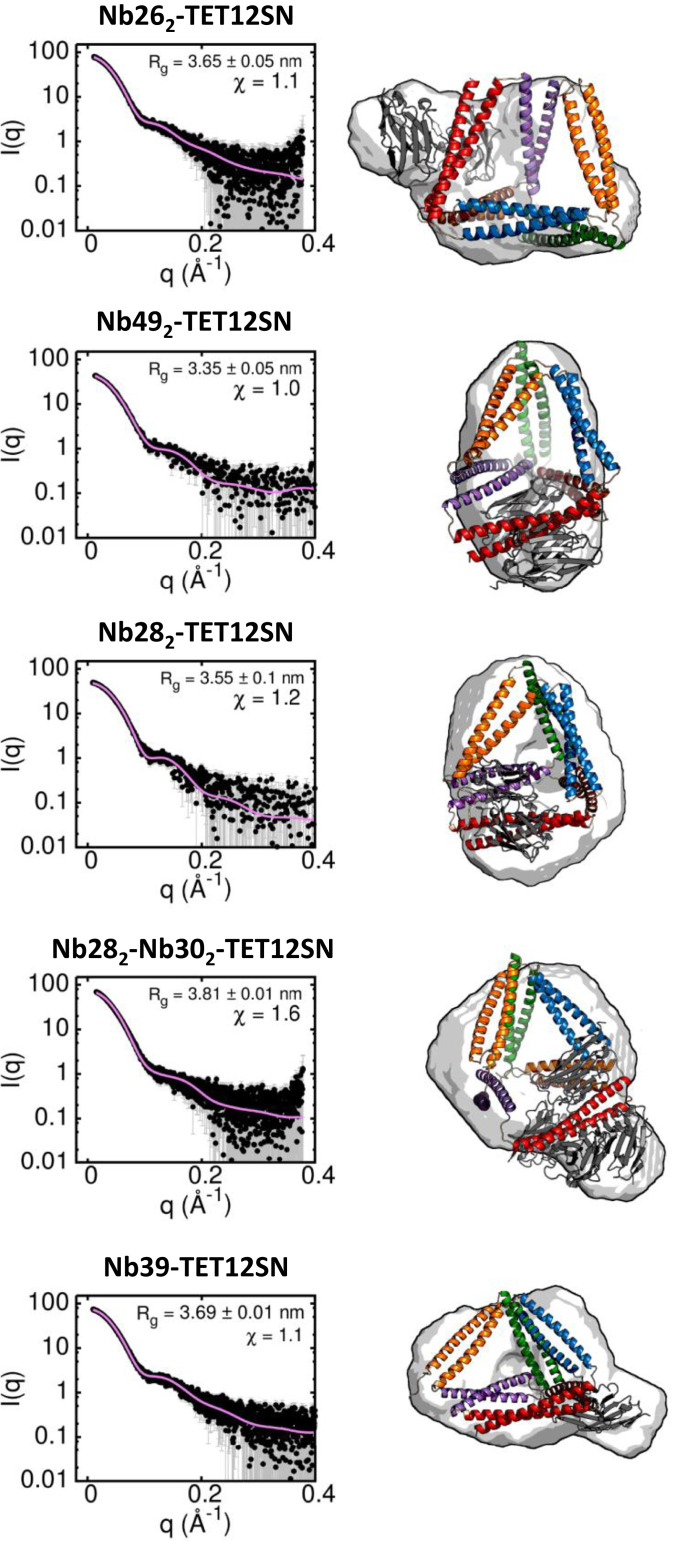
Structural analysis of the nanobody-TET12SN tetrahedron complexes using SAXS. (*Left*) Experimental SAXS profiles (black dots) together with theoretical scattering calculated from best-fitting model complex structures (pink curve). (*Right*) The best fit models of a complex with their corresponding molecular envelopes obtained by ab initio reconstruction. Nanobodies are shown in gray, and the CC segments in TET12SN are colored according to the color legend in Fig. 1*A*.

Next, we investigated how nanobodies interact with the full-length TET12SN. Although we could not crystallize the complex, recognition of CC modules by nanobodies in the tetrahedron TET12SN was reliably characterized by measurements of small-angle X-ray scattering (SAXS) for a series of nanobody-TET12SN complexes (Fig. 6). We first combined SAXS data with nanobody-CC crystal structures to obtain structural models of nanobody-TET12SN complexes. We obtained good agreement with the experimental SAXS data, since the generated molecular envelope was consistent with that obtained by the ab initio reconstruction. In the best-fitting nanobody-TET12SN models, we observed that the TET12SN molecule adopts slightly different conformations, indicating that the binding of different nanobodies affects the structure of the tetrahedral scaffold (Fig. 6). Nb49, for example, made the tetrahedral cage more compact, with Rg being lower than that observed for the free TET12SN (Rg = 3.4 nm) (12). An analysis of SAXS-validated models suggests that nanobodies Nb26, Nb49, Nb28, and Nb39 bind outside the TET12SN, which is reflected in an increased Rg compared with the free TET12SN, except in the case of Nb49. This suggests that nanobodies bind predominantly to residues on one planar side of CC peptide pairs as observed in the crystal structures, suggesting a very similar nanobody binding mode in the context of a tetrahedron. In the case of the allosteric nanobodies Nb28 and Nb30, SAXS analysis of the complex with TET12SN shows that both Nb30 molecules bind to the outer edge of the tetrahedron molecule, while Nb28 molecules partially enter the tetrahedron cavity (Fig. 6).

### Nanobodies Recognize Specific CC Modules in Different Protein Origami Designs.

**Fig. 7.**
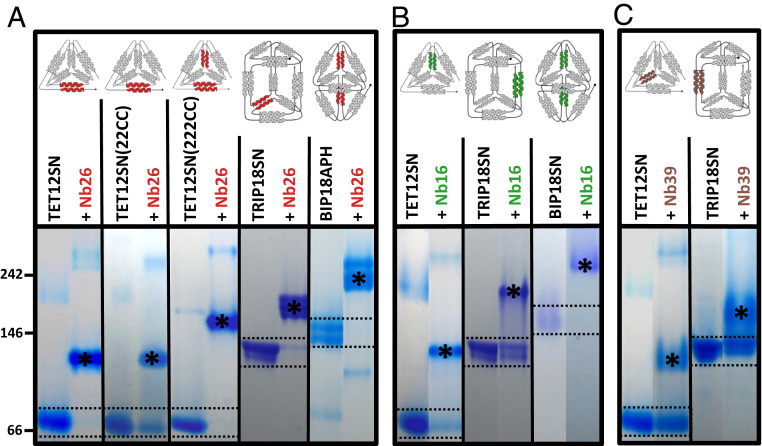
Nanobodies generated against TET12SN can specifically recognize the CC modules in different protein origami structures. Target proteins (tetrahedrons TET12SN, TET12SN(22CC), or TET12SN(222CC), triprism TRIP18SN, and bipyramids BIP18APH or BIP18SN) were incubated overnight with 5- or 10-fold molar excess of nanobodies Nb26 (*A*), Nb16 (*B*), or Nb39 (*C*) that recognize specific CC modules highlighted in the origami schemes above. The migration of protein standard (in kDa) is marked on the left side. The positions of TET12SN (53.4 kDa, pI = 4.70), TET12SN(22CC) (54.8 kDa, pI = 4.58), TET12SN(22CC) (55.2 kDa, pI = 4.56), TRIP18SN (81.9 kDa, pI = 4.69), BIP18APH (80.5 kDa, pI = 4.49), or BIP18SN (80.0 kDa, pI = 4.53) are marked by two dashed lines. The positions of complexes polyhedron-nanobody are marked with asterisks. The data are representative of at least two independent experiments.

Since CC modules APH_2_, GCN_2_, P5-P6, or BCR_2_ can be used to design various polyhedral structures, we expected that the nanobodies would bind in a modular way to these polyhedra. First, we characterized the binding of Nb26 targeting the APH_2_ module to two variants of tetrahedron with a different set and order of CC segments: the TET12SN(22CC), containing one APH_2_ module, and to TET12SN(222CC), containing two copies of the APH_2_ module. Nb26 binds with high affinity to both tetrahedral variants and, as expected, forms a larger complex with TET12SN(222CC), which has two APH_2_ modules (Fig. 7*A* and *SI Appendix*, Fig. S16). This shows that Nb26 can bind to the APH_2_ module irrespective of its exact position in the tetrahedron fold.

Next, we investigated whether nanobodies originally targeting TETSN12 would also recognize CC modules embedded in other polyhedral structures (overview of different polyhedral designs is shown in *SI Appendix*, Table S3). To this end, we used triprism TRIP18SN and two variants of bipyramid (BIP18SN and BIP18APH). The newly designed bipyramid BIP18APH harbors two APH modules and has similar bipyramidal shape, determined by SAXS, as the original BIP18SN (31) (*SI Appendix*, Fig. S17). Nb26 specifically recognizes the APH_2_ module in all these polyhedral contexts (Fig. 7*A*). In a similar manner, Nb16 can recognize BCR_2_ modules in the tetrahedron, triangular prism, and bipyramid, while Nb39 recognizes GCN_2_ modules both in the tetrahedron and triangular prism (the bipyramid lacks the GCN_2_ module) (Fig. 7 *B* and *C*). Other characterized nanobodies can specifically bind CC modules present in the triangular prism or bipyramid just as efficiently as in tetrahedron TETSN12 (*SI Appendix*, Figs. S18 and S19)

Finally, we investigated to what extent can nanobodies targeting different CC modules be combined on the same protein origami scaffold. Due to the observed binding mode on the planar sides of CC dimers (Fig. 6), we expected that different combinations of nanobodies could be used to obtain high molecular weight complexes in which the nanobodies are bound to distinct edges of the polyhedra. In fact, the analysis with native PAGE showed that different combinations of two, three, or even four nanobodies targeting different CC segments can concurrently bind tetrahedra, triangular prisms, or bipyramids (*SI Appendix*, Figs. S18–S20). These results demonstrated that in cases where nanobodies bind to different CC modules in the polyhedron, there is no steric interference between the nanobodies, resulting in large multimeric assemblies. Collectively, these results show that nanobodies can be used in a modular fashion either to target several CC segments on the same scaffold or to target one CC segment on different protein origami scaffolds.

## Discussion

**Fig. 8.**
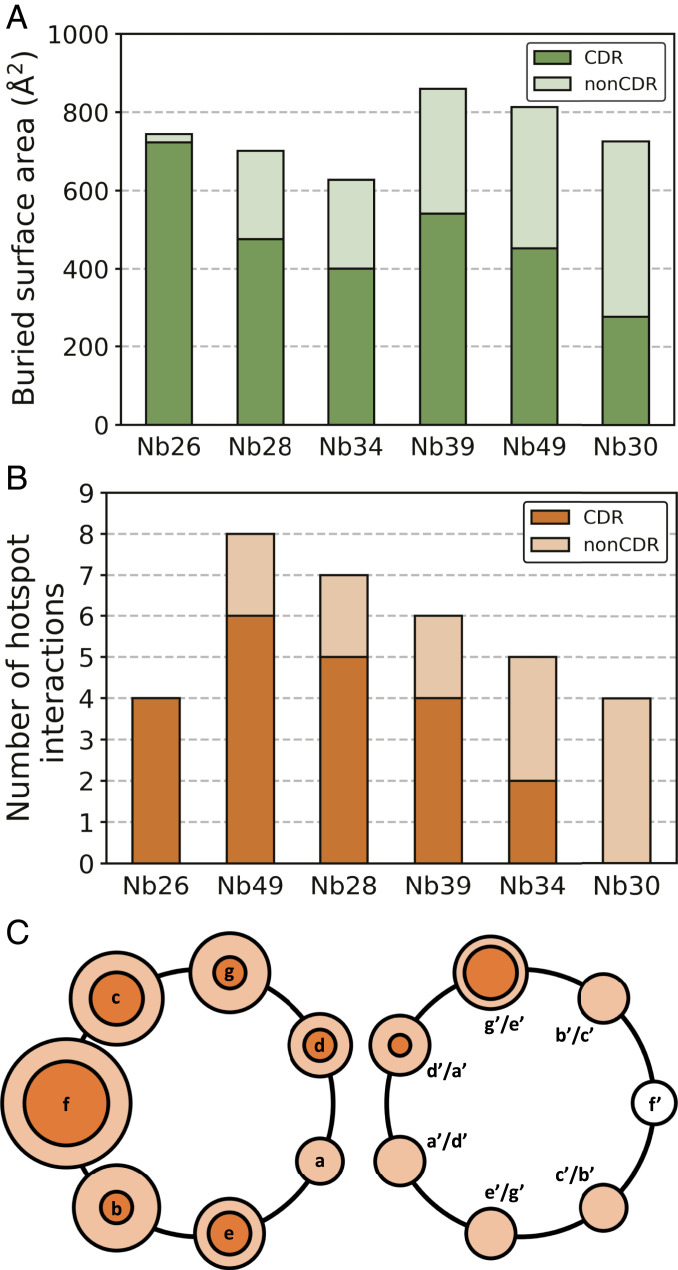
Recognition of CC dimers by nanobodies. (*A*) The amounts of solvent-buried nanobody surface for different structures show that non-CDR surfaces make a significant contribution in the stabilization of nanobody-CC complexes. (*B*) Number of hotspot interactions formed by CDR and non-CDR residues shows that CDR loops anchor the nanobodies to the CCs by forming the majority of hotspots, while non-CDR residues facilitate binding by less strong interactions. (*C*) Positions of CC interactions on the helical wheel. The radius of the bright orange circle corresponds to the cumulative number of nanobody interactions with this position for all complexes. Inter dark orange circle corresponds to the cumulative number of nanobody hotspot interactions (strong interactions). The results are combined for parallel and antiparallel CCs by considering the positions *d*’/*a*’, *g*’/*e*’, or *b*’/*c*’ as structurally equivalent.

The elongated, periodical structure of a CC dimer differs in many respects from that of globular proteins, which raises the question whether there are some characteristic patterns in the recognition of CC dimers by antibodies. This is particularly relevant for expanding the field of protein design where CCs are being used as versatile building blocks for novel protein structures. The unique feature of the nanobody-CC complexes presented here is an unusually high proportion of interactions mediated by the nanobody framework (non-CDR residues). A previous study involving a large dataset of nanobody-antigen structures showed that the average fraction of non-CDR interactions is about 15% and that these non-CDR residues are clustered at four sites of the nanobody framework (22). In contrast, we observed that the proportion of non-CDR surface area relative to whole paratope surface is even higher, between 32% (Nb28) and 62% (Nb30) (Fig. 8*A*). This is also reflected in the high proportion of interactions mediated by non-CDR residues, ranging from 32 to 67% (*SI Appendix*, Fig. S21). A notable exception is the Nb26_2_-APH_2_ complex, where the paratope is formed exclusively by CDR loops. Unprecedently, the Nb30_2_-APH_2_ interface does not involve any interaction with CDR3 and is formed almost entirely by the framework residues. Still, using computational alanine scanning, we observed that CDR loops generally mediate the majority of hotspot interactions, while non-CDR residues appear to facilitate binding via energetically weaker interactions (Fig. 8*B*). In our set of structures, the most common interaction site on the CC dimer is the position *f*, which is most solvent exposed, followed by positions *b*, *c*, *g*, and *g*’ (or a structurally equivalent position *e*’ in the case of parallel CC) on the other CC monomer (Fig. 8*C*). The epitope is centered on the position *f* and includes positions from 7 to 1 o’clock on the helical wheel. Thus, the recognition of CCs by nanobodies is mediated by CDR loops forming an anchor point by interacting with the residues at positions *f*, *c*, and *g*’, while non-CDR residues reinforce the binding via additional, mainly hydrophobic interactions. A Protein Data Bank (PDB) search identified four entries containing a nanobody bound to the CC epitope (PDB 5TD8, 6EY0, 5C3L, and 5VXM). In contrast to the complexes involving dimeric CCs presented here, the previously identified nanobody complexes involve rather large CC tetrameric helical bundles. As such, our crystal structures offer unique insights into the recognition of dimeric CCs by antibodies and highlight the importance of non-CDR residues, which are crucial for establishing a diverse and high-affinity set of nanobodies. This is important, since in the synthetic antibody libraries only residues from CDR loops are varied, while framework residues are constant.

In the CC protein origami design strategy, each CC module can be exchanged with another CC module in the same orientation, and, as we show here, the binding site of the nanobody can be inactivated by point mutation(s) without affecting the overall structure. Therefore, the data obtained from our crystal structures will be important for precise positioning of nanobody binding sites on protein origami cages. An important feature of the described set of nanobodies is their ability to recognize specific CC modules in the context of different CC protein origami designs; in other words, the polyhedral shape does not affect nanobody binding. Indeed, the nanobodies were able to recognize CC modules not only in a tetrahedron but also in a triangular prism and trigonal bipyramid, which contain these CC modules in a different topological context (Fig. 7). In a similar manner, different nanobodies can also be combined together to recognize several modules in given origami design at the same time. These results highlight the modularity of the CC recognition, which is likely related to the predominant binding of nanobodies to the outer, planar side of CC dimers in the polyhedral cages. Furthermore, we observed that the binding of a pair of nanobodies (Nb28 and Nb30) to the APH_2_ dimer is highly cooperative and that allosteric linkage is propagated through the CC alone. The comparison of APH_2_ structures as observed in complexes with different nanobodies shows small differences in the helix crossing and supercoil angles (*SI Appendix*, Fig. S22), indicating some flexibility of the APH molecule. Given that positive allosteric coupling for Nb28 and Nb30 binding is even enhanced in the context of protein origami compared with isolated APH_2_ dimer, we envision that this module could be used to engineer allosterically responsive protein origami cages. To our knowledge, the APH_2_ CC dimer represents the smallest model system in which the allosteric coupling has been described.

CC module specificity of nanobodies has the potential for the modular and context-independent introduction of selected structural domains or functionalities via nanobodies (*SI Appendix*, Fig. S23). The availability of nanobodies targeting CC modules represents a unique opportunity to address defined sites within the protein origami cages or other structures with CCs enables introduction of new functions, such as targeted delivery (32, 33) or virus-like particles for vaccines. According to the model, the volume of the cavity of the TETSN12 tetrahedron measures about 15 nm^3^, which is sufficient for the capture of small proteins or larger organic molecules. Compared with the symmetry-based designed protein self-assemblies (34, 35), where the cavity represents only a small fraction of the volume, protein origami cages can provide a much larger proportion of the cavity exposed to the solvent. Upon the binding of a nanobody, the shape of the tetrahedral molecule changed slightly. This shows that the designed CC edges are not completely rigid, most likely due to flexible linkers connecting the CC modules. The designed CC protein origami structures were shown to be biocompatible in vivo (12), while nanobodies are considered hypoimmunogenic due to their high similarity to human VH sequences and can also be humanized (36). As such, CC-specific nanobodies can be used as a tool for monitoring protein origami folding in vivo. Finally, nanobodies specific for CCs, connected with linkers or trimerization domains, and specific CC modules could be combined in different ways to build higher order two- or three-dimensional structures, not possible with the so far available tools in protein origami design. Overall, the presented set of nanobodies opens numerous possibilities for the development of advanced applications, where we can guide the design and the introduction of new functions with CC dimers and CC-specific nanobodies as modular building elements.

## Materials and Methods

Detailed materials and methods are described in *SI Appendix*, *Materials and Methods*. In short, the nanobodies specific for tetrahedron TET12SN were generated, produced, and isolated according to standard procedures (20). The identification of the specificity of the nanobodies for CC modules was performed with native PAGE. The crystals of nanobody-CC dimer complexes were grown using various commercial screens, and crystal structures were solved by molecular replacement with the relevant statistics given in *SI Appendix*, Table S1. The nanobody-TET12SN complexes were characterized by SAXS. Binding constants and stoichiometry were determined by ITC and are listed in *SI Appendix*, Table S2.

## Supplementary Material

Supplementary File

Supplementary File

Supplementary File

Supplementary File

Supplementary File

Supplementary File

Supplementary File

Supplementary File

Supplementary File

Supplementary File

## Data Availability

The atomic coordinates and structure factors have been deposited at the PDB under codes 7A50, 7A48, 7A4T, 7A4Y, and 7A4D. Uncropped scans of the native PAGE gels from Figs. 1 and 7 and *SI Appendix*, Figs. S2, S8, S9, S16, and S18–S20 are included in other supplementary material. The plasmids used in this study are available on request from R.J.
