# Supplementary material for: A nanobody toolbox targeting dimeric coiled-coil modules for functionalization of designed protein origami structures
Source: Proc Natl Acad Sci U S A. 2021 Apr 23;118(17):e2021899118. doi: 10.1073/pnas.2021899118 (PMC8092592; doi:10.1073/pnas.2021899118)
Supplement: Supplementary File [file pnas.2021899118.sapp.pdf]

PNAS
www.pnas.org

Supplementary Information for

A nanobody toolbox targeting dimeric coiled-coil modules for functionalization of designed protein origami structures

Andreja Majerle[a,1], San Hadži[a,b,1], Jana Aupič[a], Tadej Satler[a,c], Fabio Lapenta[a], Žiga Strmšek[a], Jurij Lah[b], Remy Loris[d], and Roman Jerala[a,2]

[a] Department of Synthetic Biology and Immunology, National Institute of Chemistry, SI-1000 Ljubljana, Slovenia
[b] Chair of Physical Chemistry, Faculty of Chemistry and Chemical Technology, University of Ljubljana, SI-1000 Ljubljana, Slovenia
[c] Graduate Study Programme, Faculty of Chemistry and Chemical Technology, University of Ljubljana, SI-1000 Ljubljana, Slovenia
[d] VIB-VUB Center for Structural Biology, Vrije University Brussels, B-1050 Brussels, Belgium

[1] Shared equal contribution
[2] Corresponding Author
E-mail: roman.jerala@ki.si

This PDF file includes:

SI Materials and Methods
Figures S1 to S23
Tables S1 to S3
SI References

Other supplementary materials for this manuscript include the following:

Datasets S1 to S9

SI Materials and Methods

Materials. Molecular biology reagents were from New England Biolabs (MA USA), Thermo Fisher Scientific (MA USA), Sigma-Aldrich (MO USA), or Merck (Germany), unless otherwise stated. Synthetic peptides APH (full name: APHshSN) with a sequence LEEELKQLEEELQAIEEQLAQLQWKAQARKEKLAQLKEKL), BCR (full name: BCRSN) with a sequence DIEQELERAKQSIRRLEQEVNQERSRMQYLQTLLSK, which differs from the original in the TET12SN tetrahedron in two amino acids (Q11$_f$ instead of E11$_f$ and S35$_b$ instead of E35$_b$), GCN (full name: GCNshSN) with a sequence QLEDKVEELLSKNYHLENEVERLKKLVG, P3 (full name: P3SN) with a sequence EIQQLEEEISQLEQKNSELKEKNQELKYG, P4 (full name: P4SN) with a sequence KISQLKEKIQQLKQENQQLEEENSQLEYG, P5 (full name: P5SN) with a sequence ENSQLEEKISQLKQKNSELKEEIQQLEYG, P6 (full name: P6SN) with a sequence KNSELKEEIQQLEEENQQLEEKISELKYG, P7 (full name: P7SN) with a sequence EIQQLEEKNSQLKQEISQLEEKNQELKYG, and P8 (full name: P8SN) with a sequence KISELKEENQQLEQKIQQLKEENSQLEYG were purchased from ProteoGenix (France). The N-termini of the peptides were protected by acetylation and the C-termini by amidation. The peptides were > 70 % pure and were dissolved in deionized water up to a stock concentration of about 4 mg/mL. The peptide concentrations were determined from the measured absorbance at 280 nm with extinction coefficients calculated with the PepCalc tool.

Immunization of llama for the generation of nanobodies specific for the CC protein origami tetrahedron TET12SN. All work involving animal experiments at VIB Nanobody Service Facility (Belgium) complies with the relevant ethical regulations for animal experiments and research. A llama was injected subcutaneously on days 0, 7, 14, 21, 28 and 35, each time with approximately 165 µg of purified recombinant TET12SN. Gerbu LQ#3000 was used as adjuvant. On day 40, anticoagulated blood was collected for lymphocyte preparation and library generation.

Construction of a VHH library. Total RNA from peripheral blood lymphocytes from a llama immunized with recombinant TET12SN protein was used as a template for first strand cDNA synthesis with oligo(dT) primer. Using this cDNA, the VHH encoding sequences were amplified by PCR, digested with *Pst*I and *Not*I, and cloned into the *Pst*I and *Not*I sites of the phagemid vector pHEN4. A VHH library (designated as core 65 library) of about 2×10^8 independent transformants was obtained. About 88 % of the transformants contained the vector with the correct insert size.

Isolation of antigen-specific nanobodies. For phage elution, we incubated the bound phages with 100 mM Triethylamine (Sigma-Aldrich, MO USA) for 10 minutes and then neutralize the phage elute sample with 1M Tris-HCl pH 7.4. The library was subjected to three consecutive rounds of panning on solid-phase coated recombinant TET12SN (200 µg/mL, 20 µg/well). The antigen used for panning was the same as that used for immunization. The enrichment for antigen-specific phages was evaluated after each round of panning by comparing the number of phagemid particles eluted from the antigen-coated wells with the number of phagemid particles eluted from only-blocked wells (negative control wells). These experiments indicated that the phage population was enriched approximately 30-fold and 2×10^3-fold for antigen-specific phages after 2nd and 3rd rounds of panning, respectively. After the 1st panning round there was no enrichment. 95 colonies from the 2nd round were randomly selected and analyzed by ELISA for the presence of antigen-specific nanobodies in their periplasmic extracts (ELISA using raw periplasmic extracts with soluble nanobodies). The antigen used for ELISA screening was the same as that used for immunization and panning. Of these 95 colonies, 89 colonies gave a positive result in this assay. Based on sequence

data, the 89 ELISA-positive colonies represented 29 different nanobodies belonging to 14 groups with a unique CDR3 sequence.

Recloning nanobody genes from pHEN4 to pHEN6c vector. For the recloning of nanobody genes from pHEN4 to a pHEN6c plasmid vector containing a sequence for the pelB secretion signal and the hexahistidine tag, we amplified each nanobody gene by polymerase chain reaction (PCR) using the recombinant pHEN4 plasmid containing the nanobody gene as template and primers A6E (5'-GATGTGCAGCTGCAGGAGTCTGGGGGAGG-3') and 38 (5'-GGACTAGTGCGGCCGCTGGAGACGGTGACCTGGGT-3'). The purified PCR product was digested overnight with *Pst*I, purified again and digested overnight with *BstE*II. The pHEN6c vector was digested with the same enzymes (one after the other) for 3 hours and loaded onto a 1 % (w/v) agarose gel. The cut vector was purified away from the gel and used for the ligation of the purified digested PCR product. Competent *E. coli* WK6 cells obtained from VIB Nanobody Service Facility were transformed with ligation reaction and transformants were selected using LB/Agar/Ampicillin (100 µg/mL)/Glucose (1 %) plates. Screening for positive clones was performed by PCR using universal reverse (5'-TCACACAGGAAACAGCTATGAC-3') and universal forward (5´-CGCCAGGGTTTTCCCAGTCACGAC-3') primers.

Construction of plasmids for the variants of CC protein origami tetrahedron. Plasmids for the variants of the TET12SN (TET12SN(W24A)$_1$, TET12SN(W24A)$_5$, and TET12SN(W24A)$_{1,5}$) were constructed using the Golden Gate method. For the assembly we used pTwist plasmids with CC segments present in TET12SN, where we introduced a modified sequence for the APH segment. Each final sequence for the tetrahedron construct was divided into 12 fragments, each corresponding to a CC segment. Custom Golden Gate overhangs flanking CC peptide segments on both sides were designed so that when digested with *Bsa*I overhangs allow a hierarchical and scarless assembly of basic building blocks into final constructs. Nucleotide sequence for the modified APH segment on the first position of CC building segments in the TET12SN(W24A)$_1$ or the TET12SN(W24A)$_{1,5}$ variant was 5´-atgcttgaggaagaactgaagcagttggaagaagagttgcaagcgatcgaagagcagttggcgcagctgcaggcgaaggcacaggcgcgcaaagagaagttagcgcagttaaaagaaaagttgg-3´. Nucleotide sequence for the modified APH segment on the fifth position of CC building segments in the TET12SN(W24A)$_5$ or the TET12SN(W24A)$_{1,5}$ variant was 5´-ctggaggaggaactgaagcagctggaagaagaactgcaggcgattgaagagcaactggcacagttacaggcgaaggcccaggcgcgtaaagagaaattagcgcagttgaaagagaagctgg-3´. All primers were purchased from Integrated DNA technology (IA USA). All the genes coding the proteins of interest were cloned into the expression vector pET41a+ (Genscript, NJ USA) between the restriction sites *Nde*I and *Xho*I. The design and construction of the plasmids for the tetrahedra TET12SN(22CC) and TET12SN(222CC) are described in Reference 24 in the main text.

Construction of plasmids for the fusion protein MBP-BIP18APH. Plasmids for the BIP18APH were constructed using the Golden Gate method (1). For the assembly we used pTwist plasmids with CC segments, present in the BIP18APH. Each final sequence for the bipyramid construct was divided into 18 fragments, each corresponding to a CC segment. Gibson assembly (2) was later used to introduce the DNA sequence for the maltose binding protein (MBP) tag into the gene. The genetic construct was prepared in the expression vector pET41a+ (Genscript, NJ USA) by cloning between the restriction sites *Nde*I and *Xho*I.

Expression and purification of nanobodies. Chemically competent *E. coli* WK6 cells were transformed with plasmids containing genes for representatives of the nanobody groups,

purified from *E. coli* DH5α cells by a standard plasmid DNA isolation procedure. Expression cultures were inoculated with a pre-culture at OD_{600} of 0.1 and cells were grown at 37 °C and 160 rpm in Terrific Broth (TB) medium containing 100 µg/mL Ampicillin, 0.1 % (w/v) Glucose and 2 mM $MgCl_2$ until an OD_{600} of 0.6 was reached. The temperature was lowered to 28 °C and protein expression was induced with 1 mM Isopropyl β-D-1-thiogalactopyranoside (IPTG) (GoldBio, MO USA) overnight (16-18 hours). Cells were harvested by centrifugation for 5 min at 5,500 rpm at 4 °C and the nanobodies were extracted from the periplasm. Therefore, cells were resuspended in ice-cold TES buffer composed of 0.2 M Tris-HCl pH 8.0, 0.5 mM EDTA and 0.5 M Sucrose (12 mL TES for the cell pellet from 1 L culture), and incubated with shaking on a rotating wheel for 1 hour on ice. The cell suspension was diluted with one and half volume of the ice-cold TES/4 (TES buffer diluted 4-times in deionized water), incubated with shaking for another hour on ice and centrifuged at 8000 rpm at 4 °C to isolate the supernatant containing the periplasmic extract. The his-tagged nanobodies in the periplasmic extract were filtered through 0.22 µm filter units (Sartorius, Germany) and incubated in an plastic column for 1 h at 4 °C with Ni^{2+}-NTA Sepharose (Golden Biotechnology, MO USA) previously equilibrated with Phosphate Buffer Saline (PBS). After extensive washing with PBS the bound fraction was eluted with PBS and 250 or 500 mM Imidazole. Afterwards, the samples were dialyzed in the dialysis membrane Spectra/Por® 3.5 MWCO (Spectrum Laboratories, CA USA) overnight at 4 °C against PBS or 20 mM Tris-HCl pH 7.5, 150 mM NaCl and 10 % (v/v) Glycerol, concentrated with the centrifugal unit Amicon™ 3 MWCO (Merck, Germany), shock-frozen in liquid nitrogen and stored at -80 °C. Some nanobodies were additionally purified with size exclusion chromatography (SEC). For this purification step we used the protocol and the column for the purification of CC protein origami tetrahedra. Protein concentrations were determined from the measured absorbance at 280 nm, whereby the extinction coefficients were calculated with the ProtParam tool. Prepared nanobodies were before experiments dialized against the buffer used in the particular method.

Expression and purification of the CC protein origami tetrahedra. *E. coli* NiCo21(DE3) strain (New England Biolabs, MA USA) was transformed with the construct for the tetrahedron TET12SN, TET12SN(W24A)$_1$, TET12SN(W24A)$_5$, TET12SN(W24A)$_{1,5}$, TET12SN(22CC) or TET12SN(222CC) and cultured at 37 °C overnight (160 rpm) in Luria-Bertani (LB) medium containing Kanamycin (50 µg/mL). Bacterial cultures were transferred in LB medium at OD_{600} from 0.1, grown at 37 °C until OD_{600} reached 0.6 and induced with 1 mM IPTG. After induction, the cultures were cultivated for 4 additional hours at 30 °C. The bacteria were then harvested by centrifugation (5,500 rpm at 4 °C for 5 min) and frozen. The harvested cells were resuspended in 10 mL (per liter culture) of a cold lysis buffer: 50 mM Tris-HCl pH 8.0, 150 mM NaCl, 10 mM Imidazole, 1 mM $MgCl_2$, 0.5 mg/mL Lysozyme, 0.06 µl/mL Benzonase (250 U/mL) and 2 µL/mL CPI (Protease Inhibitor Coctail). Cell lysis was completed by ultrasonication with a Vibra-cell VCX (Sonics, CT USA) on ice for maximum of seven cycles of 1 minute total pulse, at intervals of 1 s pulse and 3 s pause (50 % amplitude). Subsequently, cellular lysates were centrifuged at 16,000 x g at 4 °C for 30 min, the respective soluble fractions were filtered through 0.2 µm filter units (Sartorius, Germany) and incubated in an plastic column for 1 h at 4 °C with Ni^{2+}-NTA resin (Golden Biotechnology, MO USA), which had previously been equilibrated with buffer A (50 mM Tris-HCl pH 8.0, 150 mM NaCl and 10 mM Imidazole). After extensive washing with buffer A or buffer B (50 mM Tris-HCl pH 8.0, 150 mM NaCl and 20 mM Imidazole) the bound fraction was eluted with buffer C (50 mM Tris-HCl pH 8.0, 150 mM NaCl and 250 mM Imidazole). After filtration in 0.22 µm syringe filters (Sigma-Aldrich, MO USA) the eluted fractions were injected onto a size exclusion column with HiLoad 16/600 Superdex 200 prep grade resin (GE Healthcare, IL USA), packed into a 26/600 XK column (GE Healthcare, IL USA) and separated at 2.6 mL/min in a degassed SEC buffer (20 mM Tris-HCl pH 7.5, 150 mM NaCl and 10 % (v/v) Glycerol). Chromatography was performed with the FPLC system ÄKTA pure (GE Healthcare, IL USA) in SEC buffer at a

linear flow rate of 2.6 mL/min and the eluted protein fractions were collected separately. The respective monomer peaks were pooled, concentrated with the centrifugal filters Amicon^{TM} Ultra 10 MWCO (Sigma-Aldrich, MO USA), shock-frozen in liquid nitrogen and stored at −80 °C. The protein concentrations were determined from the measured absorbance at 280 nm with extinction coefficients calculated with the ProtParam tool.

Expression and purification of the triangular prism TRIP18SN. TRIP18SN was produced and isolated by the procedure described in Reference 12 cited in the main text.

Expression and purification of the trigonal bipyramid BIP18APH. BIP18APH was expressed in *E. coli* NiCo21(DE3) strain as a fusion protein with MBP tag on N-terminus after the induction with 1 mM IPTG and cultivation for 4 hours at 30 °C. Harvested cells were resuspended on ice in lysis buffer (50 mM Tris-HCl pH 8.0, 150 mM NaCl, 10 mM Imidazole, 1 mM $MgCl_2$, 0.5 mg/mL Lysozyme, 15 U/mL Benzonase and CPI protease inhibitor mix) and lysed with ultrasonication for 15 min with intervals of 1 s pulse and 3 s pause (50 % amplitude). Afterwards, cellular lysates were centrifuged at 16,000 g and at 4 °C for 30 min, respective soluble fractions were filtered through 0.2 μm filter (Sartorius, Germany) and applied to Ni^{2+}-NTA resin (Golden Biotechnology, MO USA). After washing with buffer A (50 mM Tris-HCl pH 8.0, 150 mM NaCl and 10 mM Imidazole) or buffer B (50 mM Tris-HCl pH 8.0, 150 mM NaCl and 20 mM Imidazole) the bound fraction was eluted with buffer C (50 mM Tris-HCl pH 8.0, 150 mM NaCl and 250 mM Imidazole) and processed with TEV (Tobacco Etch Virus) protease 2 hours at room temperature and then overnight at 4 °C (the concentration of proteins from the eluted fraction was 0.1 to 0.5 mg/mL, while the concentration of TEV protease was 125 μg/mL). Subsequently, protein sample was filtered through 0.2 μm filter and injected onto an ion exchange column with anionic exchanger DEAE-Sepharose (GE Healthcare, IL USA). After washing with IEX buffer A (50 mM Tris-HCl pH 8.0 and 150 mM NaCl) we started a gradient elution with IEX buffer B (50 mM Tris-HCl pH 8.0, 2 M NaCl) to a final concentration of 650 mM NaCl in 50 minutes. Eluted protein was collected and again applied to the Ni^{2+}-NTA resin. Flow-through sample was collected, concentrated, injected onto a size exclusion column (HiLoad 16/600 Superdex 200 prep grade) and separated in SEC buffer (20 mM Tris-HCl pH 7.5, 150 mM NaCl and 10 % (v/v) Glycerol) at a flow rate of 2.6 mL/min. Protein sample from monomeric peak was collected, concentrated with the centrifugal filters Amicon^{TM} Ultra 10 MWCO, shock-frozen in liquid nitrogen and stored at -80 °C.

Expression and purification of the trigonal bipyramid BIP18SN. Protein was produced and isolated by the procedure described in Reference 31, cited in the main text.

Characterization of isolated proteins with sodium dodecyl sulphate polyacrylamide gel electrophoresis (SDS PAGE). The isolated proteins were analysed with SDS PAGE in the apparatus mini-PROTEAN™ (Bio-Rad, CA USA) in 12 or 15 % (w/v) discontinuous polyacrylamide gels pH 8.8 with sodium dodecyl sulphate (SDS), loaded next to a pre-stained molecular ruler (Thermo Fisher Scientific, MA USA). SDS PAGE was performed in the electrophoresis buffer (25 mM Tris-HCl pH 8.3, 0.1 % (w/v) SDS and 192 mM Gylcine) at 200 V for 50 minutes. For loading the samples we used 4x stock solution (250 mM Tris-HCl pH 6.8, 8 % (v/v) SDS, 40 % (v/v) Glycerol, 20 % (v/v) β-Mercaptoethanol, 0.2 % (v/v) Bromphenol Blue). The gels were stained with Coomassie staining solution (80 % Ethanol, 20 % Acetic acid and 4 % Coomassie Brilliant Blue R250) for 30 min and then destained overnight in a destaining solution (20 % Ethanol, 10 % Acetic acid) or the staining agent InstantBlue^{TM} (Sigma-Aldrich, MO USA) according to the manufacturer´s instructions and scanned for analysis.

Native PAGE. To identify nanobody binding to CC protein origami cages, proteins at a concentration of 5 µM were incubated with nanobodies in five or tenfold molar excess overnight at 4 °C in 20 mM Tris-HCl pH 7.5 and 150 mM NaCl. To identify nanobody specificity for CC modules in the CC protein origami tetrahedron TET12SN, this protein was incubated overnight at 5 µM concentration with nanobodies in five or tenfold molar excess and a CC peptide pair in 12.5-, 25- or 50-fold molar excess (dimer concentration) in 20 mM Tris-HCl pH 7.5 and 150 mM NaCl at 4 °C. Native PAGE was run in the apparatus mini-PROTEAN™ (Bio-Rad, CA USA) on an 8 % (w/v) discontinuous polyacrylamide gel (pH 8.8) in cold electrophoresis buffer (25 mM Tris-HCl pH 8.3 and 192 mM Gylcine) at 130 V for 2 hours. The loading buffer was without SDS and β-Mercaptoethanol. The samples were loaded next to the unstained protein standard NativeMark™ (Thermo Fisher Scientific, MA USA). The gels were stained in Coomassie staining solution for 30 minutes and then destained overnight in destaining solution or with the staining agent InstantBlue™ according to the manufacturer´s instructions and scanned for analysis. Uncropped scans of native gels are included in the Dataset.

Circular dichroism (CD) spectrometry. CD spectra and thermal denaturation curves were recorded with the instrument ChiraScan (Applied Photophysics, UK), equipped with a Peltier thermal control block (Melcor, NJ, now part of Laird Technologies). CD spectra were recorded in a 1 mm quartz cuvette (Hellma, Germany) in far-UV, from 200 to 280 nm, with a step size of 1 nm, a bandwidth of 1 nm and an integration time of 1 s. The spectra and denaturation curves of the individual TET12SN variants were measured at 5 µM concentration. All measurements were performed in triplicates and then averaged. Temperature denaturation experiments were performed by heating the samples from 10 to 90 °C at a rate of 1 °C/min, and the CD signal was measured at 222 nm in 1 °C steps. At the end of the thermal denaturation scan another CD spectrum was measured at the final temperature. Finally, the sample was quickly cooled down to 20 °C, and another CD spectrum was measured. The effective sample temperature was monitored by a temperature probe inserted into the cuvette. The melting temperatures were determined by fitting a thermodynamic model to experimental CD data as described in reference 3.

Size-exclusion chromatography coupled with multi-angle light scattering (SEC-MALS). SEC-MALS experiment with BIP18APH was performed on the HPLC system e2695 coupled with the UV detector 2489 (Waters, MA USA), the multiple-angle light scattering detector Dawn8+ (Wyatt, CA USA) and the refractive index (RI) detector RI500 (Shodex, Japan). The protein sample was filtered with the centrifugal filter Durapore™ 0.1 µm (Sigma-Aldrich, MO USA), after which 100 µL was injected onto a Superdex 200 Increase 10/300 column (GE Healthcare, IL USA) previously equilibrated with SEC buffer (20 mM Tris-HCl pH 7.5 and 150 mM NaCl). Chromatography was performed at a flow rate of 0.5 mL/min. Data analysis was carried out with the software Astra 7.0 (Wyatt, CA, USA) utilizing the RI signal as concentration source.

Dynamic light scattering (DLS). The size of the isolated BIP18APH was measured on a Zetasizer Nano (Malvern Panalytical, UK) at 20 °C using an angle of 173° and a 633 nm laser. The size distribution of the particles was recorded and a hydrodynamic diameter was calculated with the software provided by the manufacturer.

Small-angle X-ray scattering (SAXS). Scattering curves were measured at the P12 beamline of PETRA III - DESY (Hamburg, Germany) (4) and the SIBYLS beamline (5) at ALS (Berkeley, CA USA). Before each measurement, unbound nanobodies were separated from the TET12SN-nanobody complex by performing online SEC the with a Superdex 200 Increase 10/300 column (GE Healthcare, IL USA) in SEC buffer (20 mM Tris-HCl pH 7.5, 150

mM NaCl and 2 or 10 % (v/v) Glycerol). The mobile phase was flown into the column at a flow rate of 0.5 mL/min. At PETRA-III synchrotron X-ray wavelength was 1.24 Å, the Pilatus 6M detector was positioned 3 m from the sample and the scattering vector ranged from 0.028 - 7.3 nm^{-1}. 3000 - 3600 scattering frames were collected with an exposure time of 0.995 s. SAXS experiments at SIBYLS beamline were performed at an X-ray wavelength of 1.03 Å with a distance between sample and detector (Pilatus3 2M pixel array detector) of 1.5 m. The scattering vector ranged from 0.13 to 5 nm^{-1}. 800 scattering frames were collected, each resulting from a 3 s exposure. Frames belonging to the peak of interest were averaged. The contribution of the mobile phase was removed from the scattering curve by subtracting the averaged buffer scattering. The scattering curves were analyzed with the software PRIMUS (6). *Ab initio* modelling was performed with DAMMIF/DAMAVER programs (7). SAXS profiles of molecular models were calculated and compared with experimental data using Pepsi-SAXS (8). The models were fitted to the experimental SAXS profiles and scored according to the χ values, with the lowest values being the best.

Isothermal titration calorimetry (ITC). Before the experiment, all samples were dialyzed three times against phosphate buffer (20 mM sodium phosphate buffer pH 7.0 and 150 mM NaCl), centrifuged for 10 minutes and degassed. The experiments were performed with a MicroCal VP-ITC instrument (Malvern Panalytical, UK) at 25 °C, unless otherwise specified. In most cases, the nanobody solution was titrated into the cell containing the CC protein origami tetrahedron at 2-5 μM or CC-forming synthetic peptides at 5-10 μM concentration. Raw thermograms were integrated with the software NITPIC (9, 10) , and the heat changes were then analyzed with SEDPHAT (11). Parameter and error analysis was performed using the Monte-Carlo approach for nonlinear regression with 1000 iterations as implemented in SEDPHAT. The heat capacity was determined from the titrations carried out at 18, 25 and 32 °C, which were fit globally assuming a temperature-independent heat capacity increment in the studied temperature interval.

Crystallization and structure determination. Protein complexes in 20 mM Tris-HCl pH 7.5 and 150 mM NaCl were prepared by mixing a peptide dimer with the 1.2 molar excess of the corresponding nanobody and crystallized using the sitting drop method. Samples were concentrated to 10 mg/mL and dispensed to crystal plates using the Phoenix protein crystallization robot (Art Robbins Instruments). Drops consisting of 0.2 μL of the protein and precipitant solution were equilibrated against 60 μL of participant solution. Various commercial screens were used: Crystal Screen and Crystal Screen 2 (Hampton Research), Morpheus, PACT premier and ProPlex (Molecular Dimensions, UK) and Jena Classic (Jena Bioscience, Germany). All final crystallization conditions are listed in Table S1. Crystals were cryoprotected and frozen in liquid nitrogen. All data were measured at the SOLEIL synchrotron (Gif- sur-Yvette, France) at 100K on beamline PROXIMA1 using a PILATUS 6M detector. All data were indexed, integrated and scaled with XDS (12). Structures were solved by molecular replacement using PHASER-MR (13) using nanobody and coiled-coil as search models. Homology models of nanobody were obtained using MODELER (14) and the search model had truncated CDR loops. Coiled-coil models were built using the ISAMBARD Python package (an open source) (15). In all cases, the structures were manually rebuilt with Coot (16) and refined with phenix.refine (17). The final refinement cycles included TLS refinement (one TLS group per chain). Data collection and refinement statistics are given in Table S1.

Analysis of crystal structures. The solvent accessible surface area was calculated with the NACCESS using default parameters (18). Protein-protein interactions were analyzed with PISA (19). In addition, a home-written script was used to analyze interchain close-contacts, which are pairs of residues where any heavy atom is closer or equal to 4 Å to that of another residue. Hotspot interactions were identified using computational alanine scanning using

Robbeta (20). Residues that mediate a hotspot interaction were defined as those where the substitution to alanine disrupts the complex by >1kcal/mol. The results from Robbeta were also consistent with those obtained with BUDDE and FoldX (21). The ISAMBARD Python package (an open source) was used to extract geometric parameters from the APH crystal structures and to build the unbound APH model structure based on Crick's generalized parametric equations for describing coiled-coil backbone structures (22). The structural parameters (superhelix radius, pitch, and Crick angle) were optimized using a genetic algorithm. The internal energy was evaluated using the BUDE force field (23).

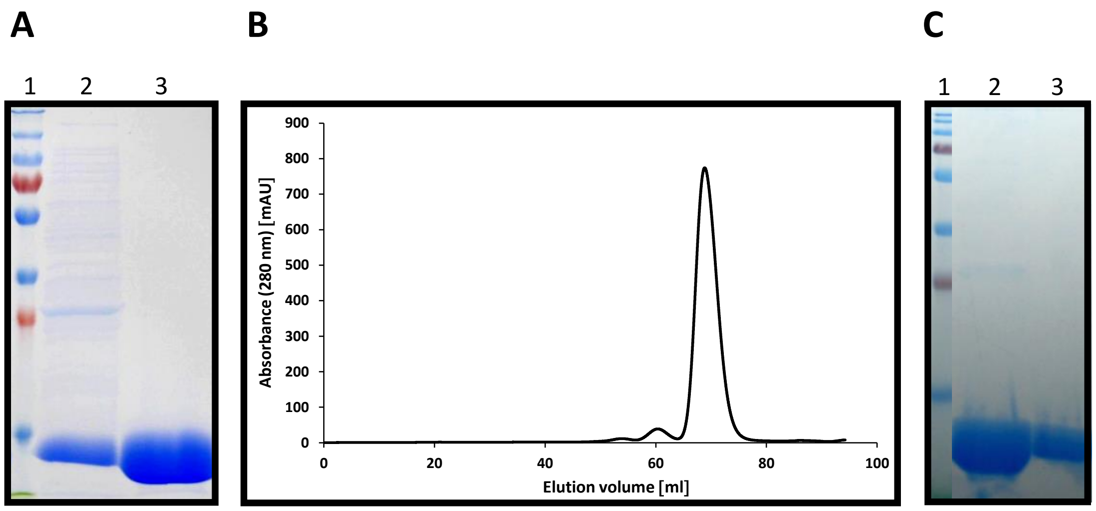

Fig. S1. Expression and purification of nanobodies specific for the CC protein origami tetrahedron TET12SN. Nanobodies as soluble C-terminal His_6 tagged proteins with a molecular weight around 14 kDa were produced in *E. coli* strain WK6, extracted from the periplasm by osmotic shock and purified by Ni^{2+}-affinity chromatography (***A***). Up to 30 milligrams of nanobodies using only Ni^{2+}-affinity chromatography were obtained from one liter of bacterial culture. Some nanobodies were additionally purified by size exclusion chromatography (SEC) (***B***). Figure in panel ***A*** shows SDS PAGE analysis of the isolation of a nanobody (Nb49) produced in *E. coli* on a 12 % (w/v) SDS gel (lane 1, sizes of proteins (in kDa) in the protein standard: from the top 250, 130, 100, 75, 55, 35, 25, 15 and 10; lane 2, proteins in the periplasmic extract; lane 3, sample eluted with 500 mM Imidazole from Ni^{2+}-NTA resin and dialysed against PBS). The gel was stained in Coomassie protein staining solution. Figure in panel ***B*** shows a SEC profile with a symmetrical peak for nanobody Nb30, reflecting the good solubility and homogeneity of the nanobodies. Figure in panel ***C*** shows SDS PAGE analysis of additional isolation of nanobody Nb30 with SEC on a 15 % (w/v) SDS gel (lane 1, proteins in the protein standard with the same sizes (in kDa) as in panel ***A***, lane 2, sample eluted with 500 mM Imidazole from Ni^{2+}-NTA resin and dialysed against PBS; lane 3, eluted fraction from SEC column). The gel was stained with the staining agent InstantBlue^{TM}.

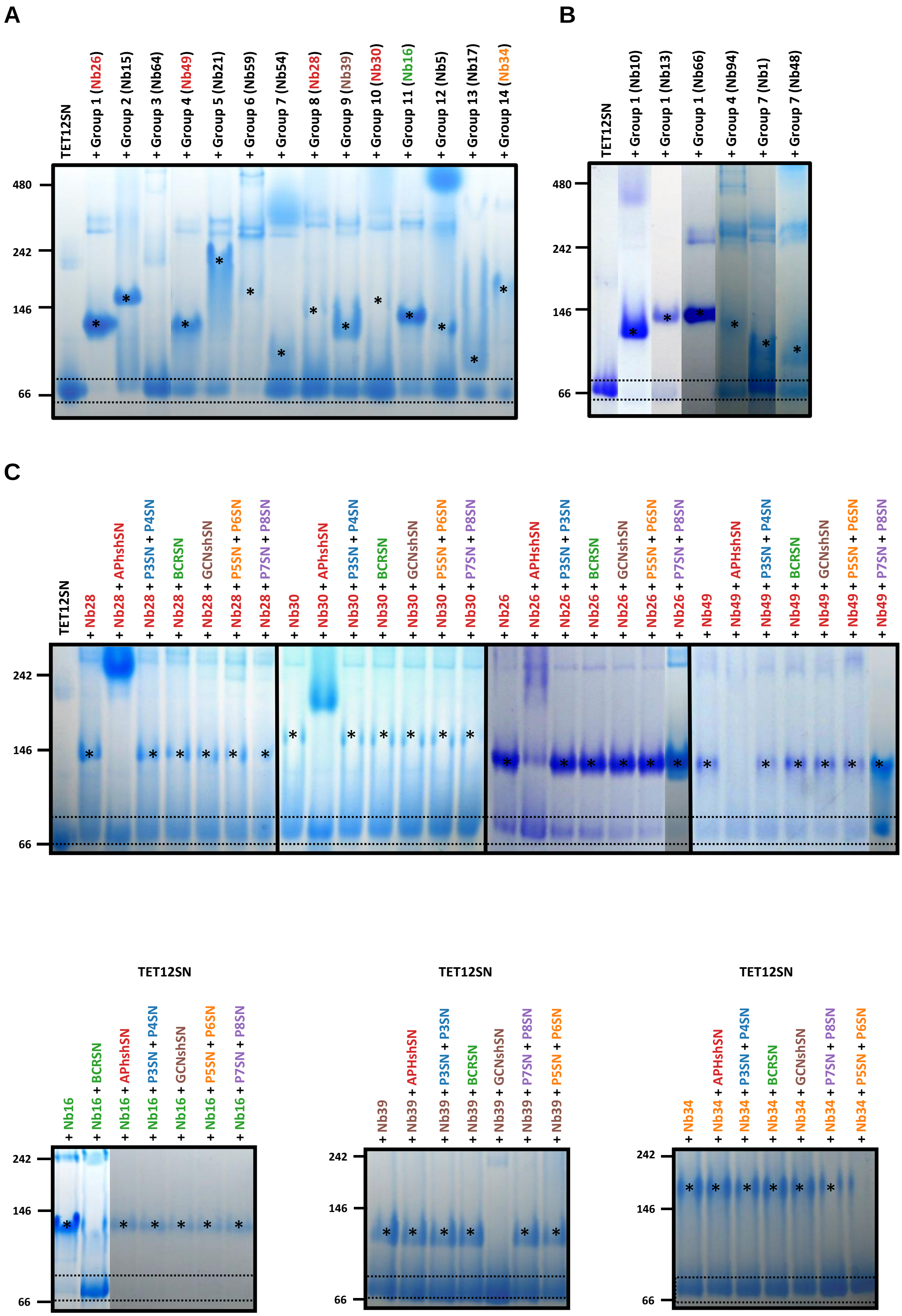
A
B
C
TET12SN
+ Group 1 (Nb26)
+ Group 2 (Nb15)
+ Group 3 (Nb64)
+ Group 4 (Nb49)
+ Group 5 (Nb21)
+ Group 6 (Nb59)
+ Group 7 (Nb54)
+ Group 8 (Nb28)
+ Group 9 (Nb39)
+ Group 10 (Nb30)
+ Group 11 (Nb16)
+ Group 12 (Nb5)
+ Group 13 (Nb17)
+ Group 14 (Nb34)
+ Group 1 (Nb10)
+ Group 1 (Nb13)
+ Group 1 (Nb66)
+ Group 4 (Nb94)
+ Group 7 (Nb1)
+ Group 7 (Nb48)
480
242
146
66
+ Nb28
+ Nb28 + APhshSN
+ Nb28 + P3SN + P4SN
+ Nb28 + BCRSN
+ Nb28 + GCNshSN
+ Nb28 + P5SN + P6SN
+ Nb28 + P7SN + P8SN
+ Nb30
+ Nb30 + APhshSN
+ Nb30 + P3SN + P4SN
+ Nb30 + BCRSN
+ Nb30 + GCNshSN
+ Nb30 + P5SN + P6SN
+ Nb30 + P7SN + P8SN
+ Nb26
+ Nb26 + APHshSN
+ Nb26 + P3SN + P3SN
+ Nb26 + BCRSN
+ Nb26 + GCNshSN
+ Nb26 + P5SN + P6SN
+ Nb26 + P7SN + P8SN
+ Nb49
+ Nb49 + APHshSN
+ Nb49 + P3SN + P4SN
+ Nb49 + BCRSN
+ Nb49 + GCNshSN
+ Nb49 + P5SN + P6SN
+ Nb49 + P7SN + P8SN
+ Nb16
+ Nb16 + BCRSN
+ Nb16 + APhshSN
+ Nb16 + P3SN + P4SN
+ Nb16 + GCNshSN
+ Nb16 + P5SN + P6SN
+ Nb16 + P7SN + P8SN
+ Nb39
+ Nb39 + APHshSN
+ Nb39 + P3SN + P3SN
+ Nb39 + BCRSN
+ Nb39 + GCNshSN
+ Nb39 + P7SN + P8SN
+ Nb39 + P5SN + P6SN
+ Nb34
+ Nb34 + APHshSN
+ Nb34 + P3SN + P4SN
+ Nb34 + BCRSN
+ Nb34 + GCNshSN
+ Nb34 + P7SN + P8SN
+ Nb34 + P5SN + P6SN

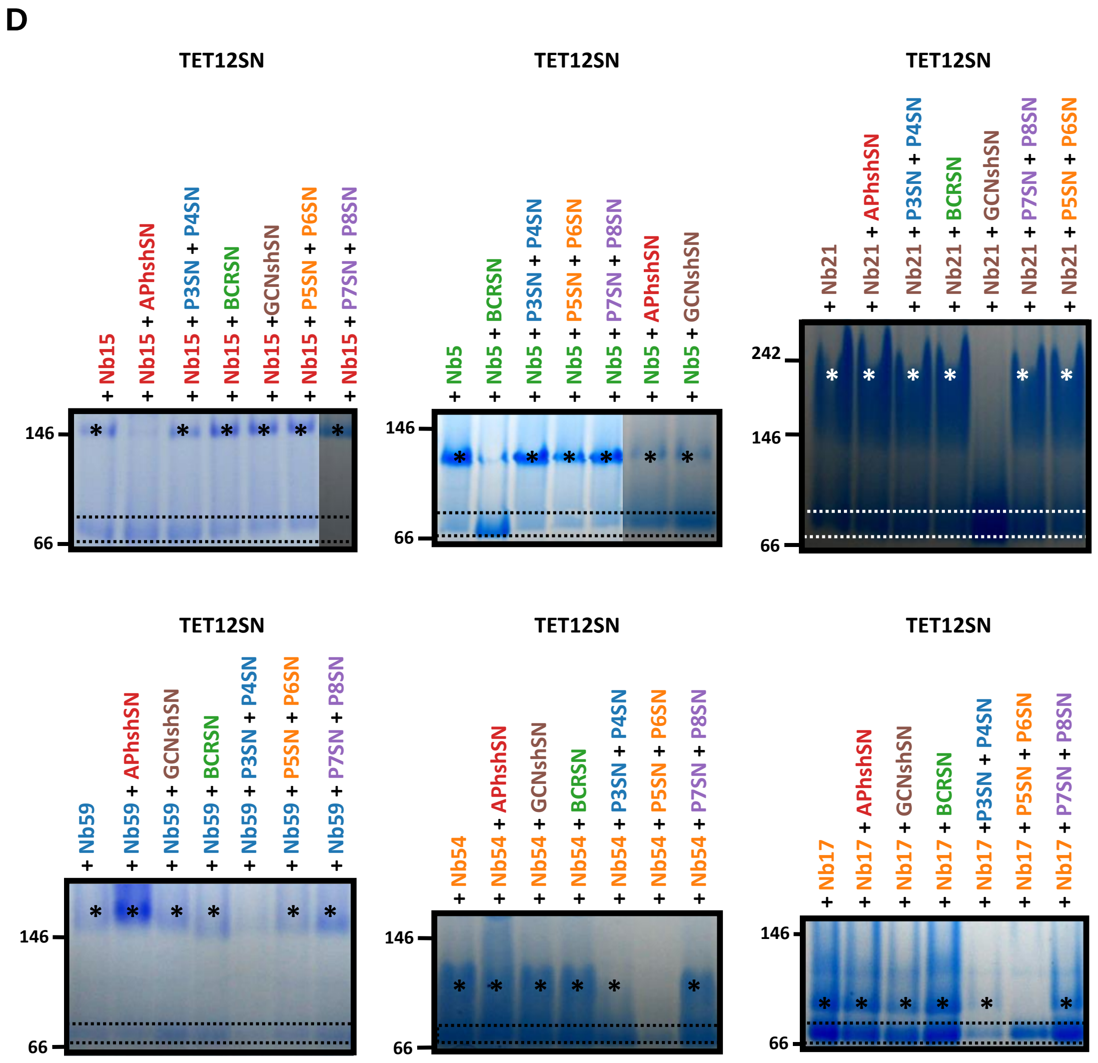

Fig. S2. Identification of the specificity of nanobodies to CC modules in the tetrahedron TET12SN. (***A*** and ***B***) The target protein (TET12SN) (5 µM) was incubated with individual nanobodies in five or tenfold molar excess overnight in 20 mM Tris-HCl pH 7.5 and 150 mM NaCl at 4 °C. Binding of nanobodies to TET12SN was analyzed on 8 % (w/v) native gels (pH 8.8) at 130 V for 2 hours. Gels were stained with the staining agent InstantBlue^{TM} or in Coomassie protein staining solution. The sizes of the proteins (in kDa) in the unstained protein standard NativeMark^{TM} are marked on the left side. The positions of TET12SN itself (53.4 kDa, pI = 4.70) are marked by two dashed lines. The positions of the complexes consisting of TET12SN and nanobody are marked with asterisks. In panel ***A*** are shown the results of experiments for binding of one member of each nanobody group to TET12SN. Bands for free nanobodies Nb54 (14.5 kDa, pI = 7.18) and Nb5 (14.3 kDa, pI = 7.99) are on positions between 146 and 480 kDa. Free nanobodies Nb26 (14.5 kDa, pI = 8.58), Nb28 (13.6 kDa, pI = 8.40) and Nb30 (13.5 kDa, pI = 8.02) migrate slowly, their bands are above 480 kDa and are not shown in the figure. Owing to their high isoelectric point free Nb15 (14.2 kDa, pI = 8.98), Nb16 (13.7 kDa, pI = 9.01), Nb17 (13.5 kDa, pI = 9.51), Nb21 (14.0 kDa, pI = 9.26), Nb34 (13.0 kDa, pI = 9.51), Nb39 (14.0 kDa, pI = 9.01), Nb49 (13.8 kDa, pI = 9.39) and Nb59 (13.8 kDa, pI = 9.04) are to positively charged to run into the native gel. In panel ***B*** are shown the results of experiments for binding of other members of nanobody groups 1, 4 and 7 to TET12SN. Bands for free nanobody Nb1 (13.8 kDa, pI = 7.17) is on position between 242 and 480 kDa. Free nanobodies Nb10 (14.5 kDa, pI = 7.97) and Nb48 (14.4 kDa, pI = 8.00) are on positions around 480 kDa. Free nanobodies Nb13 (14.5 kDa, pI = 8.58) and Nb66 (14.4 kDa, pI = 8.58) migrate slowly, their bands are above 480 kDa and are not shown in the figure. Owing to its high isoelectric point free Nb94 (13.8 kDa, pI = 9.39) is to positively charged to

run into the native gel. The characterized nanobodies Nb26, Nb28, Nb30, Nb34, Nb39 and Nb49 are highlighted with the color of the target CC module. (***C** and **D***) Competition experiments for the determination of nanobodies targeting CC modules in TET12SN. Target protein (TET12SN) (5 µM) was incubated overnight with an individual nanobody in five or tenfold molar excess and a pair of synthetic peptides (a peptide APHshSN, BCRSN or GCNshSN forming a homodimer (APH_2, BCR_2, or GCN_2) or a pair of peptides P3SN and P4SN, P5SN and P6SN or P7SN and P8SN forming a heterodimer (P3-P4, P5-P6 or P7-P8)) in 25- or 50-fold molar excess in 20 mM Tris-HCl pH 7.5 and 150 mM NaCl at 4 °C. Binding of nanobodies to TET12SN was analyzed on 8 % (w/v) native gels (pH 8.8) at 130 V for 2 hours. Gels were stained in Coomassie staining solution or with the staining agent InstantBlue^{TM}. Sizes of proteins (in kDa) in the unstained protein standard NativeMark^{TM} are marked on the left side of each separate picture. The positions of TET12SN (53.4 kDa, pI = 4.70) itself are marked by two dashed lines. The positions of the complexes composed of TET12SN and nanobodies are marked with asterisks. In panel ***C*** are presented the results of experiments with characterized nanobodies targeting APH_2, BCR_2, GCN_2 or P5-P6 module (Nb26, Nb28, Nb30, Nb49, Nb16, Nb39 and Nb34). In panel ***D*** are the results of competition experiments with other nanobodies shown in panel ***A***, except of Nb64 (Nb15 targeting APH_2, Nb5 targeting BCR_2, Nb21 targeting GCN_2, Nb59 targeting P3-P4, Nb54 targeting P5-P6 and Nb17 targeting P5-P6 module). All experiments were performed at least twice.

```
                                                           CDR1                            CDR2
Nb26 (Group 1)      QVQLQESGGGLVQAGDSLRLSCAASGRTFSTYPMGWFRQAPGKEREFVAASS---SRAYY      57
Nb49 (Group 4)      QVQLQESGGGLVQPGGSLRLSCIASGRTFNPYGMGWFRQVPGKERTFVSGITWIGGTTYY      60
Nb28 (Group 8)      QVQLQESGGGLVQPGGSLRLSCAAPGFRLDNYVIGWFRQAPGKEREGVSCISSSAGSTYY      60
Nb39 (Group 9)      QVQLQESGGGLVQAGGSLRLSCAASGSIFSINVMGWYRQAPGKQRELLASITSR-GSTNY      59
Nb30 (Group 10)     QVQLQESGGGLVQPGGSLRLSCAASGSIFSDNDMGWYRQPPGKQREWVATITYD-HVTWY      59
Nb34 (Group 14)     QVQLQESGGGLVQPGGSLRLSCAASQFTFSSDWMYWVRQAPGKGLEWVSSISPGGAATAY      60

                                                                   CDR3
Nb26 (Group 1)      ADSVKGRFTISRNNAKNTVYLQMNSLKPEDTAVYYCVADSSPYYRRYDAAQDYDYWGQGT     117
Nb49 (Group 4)      VNSVKGRFTISRDRAKNTVYLQMNSLKPEDTAVYYCAADK--------DNTGYNYWGQGT     112
Nb28 (Group 8)      ADSVKGRFTISRDNAKNTVYLQMNSLKPEDTAVYYCATA--------CYSSYVTYWGQGT     112
Nb39 (Group 9)      ADSVKDRFTISRDNAKNTVYLQINSLKPEDTAVYYCNSRGW-----TTTRGDYDYWGQGT     114
Nb30 (Group 10)     ADSVKGRFAISRDNAKNTVYLQMNDLKPEDTAVYYCNAV----------PGRRGSWGQGT     109
Nb34 (Group 14)     AASVKGRFTISRDNAKNTLYLQMNSLKSEDTAVYYCSKTRA---------G--TGRGQGT     109

Nb26 (Group 1)      QVTVSSHHHHHH                                                    129
Nb49 (Group 4)      QVTVSSHHHHHH                                                    124
Nb28 (Group 8)      QVTVSSHHHHHH                                                    124
Nb39 (Group 9)      QVTVSSHHHHHH                                                    126
Nb30 (Group 10)     QVTVSSHHHHHH                                                    121
Nb34 (Group 14)     QVTVSSHHHHHH                                                    121
```

Fig. S3. Characterized nanobodies interact with coiled-coil dimers with high proportion of amino acids in non-CDR regions. Amino acid sequence alignment of characterized nanobodies Nb26, Nb49, Nb28, Nb39, Nb30 and Nb34. Amino acids in CDRs or non-CDRs which in the crystal structure (Nb26$_2$-APH$_2$, Nb49$_2$-APH$_2$, Nb28$_2$-Nb30$_2$-APH$_2$, Nb39-GCN$_2$ or Nb34-P5-P6) interact with amino acids in coiled-coil dimer are colored (residues in CDR loops with dark orange and non-CDR residues with bright orange). Multiple sequence alignment was done with Clustal Omega tool.

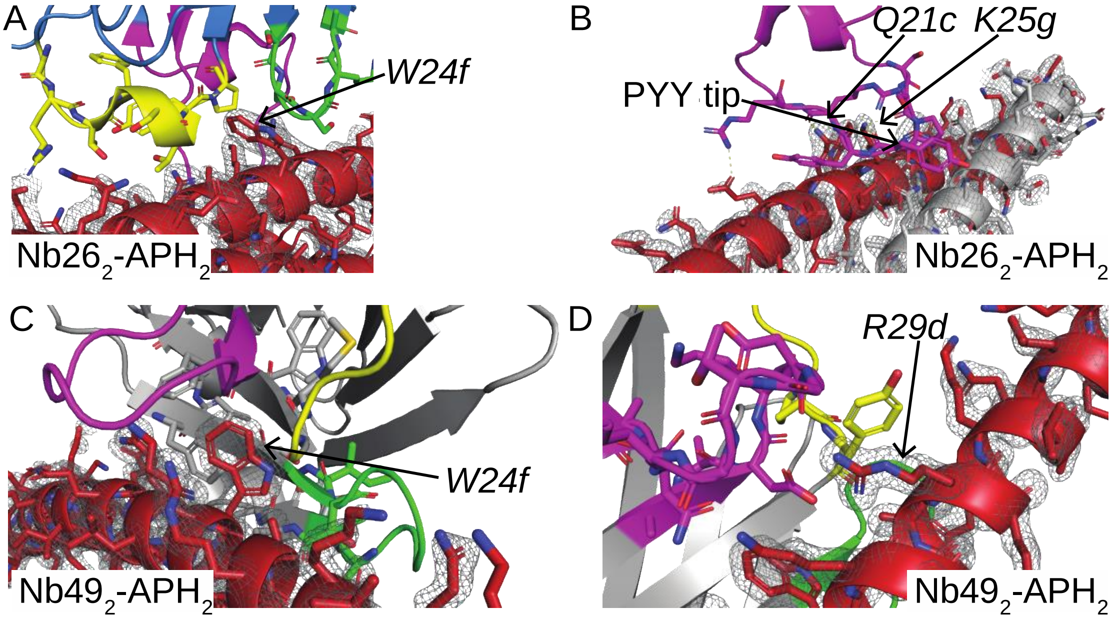

Fig. S4. Interactions between nanobody Nb26 (blue) or nanobody Nb49 (gray) and the antiparallel APH coiled-coil (chain A- red, chain B-gray) in the Nb26$_2$-APH$_2$ or Nb49$_2$-APH$_2$ complex. Nanobody residues are labeled in regular font, APH residues are labeled in italic, according to the sequence number and its position of the heptad repeat (*a-f* for chain A or *a'-f'* for chain B). Electron density (2Fo-Fc map are contoured at 1 sigma) is shown only for coiled-coil for clarity. CDR loops are shown in yellow (CDR1), green (CDR2) and pink (CDR3). (***A***) Nb26 CDR loops CDR1 (yellow) and CDR2 (green) shield the solvent exposed W24 at position *f* on the APH coiled-coil. (***B***) Interactions between Nb26 CDR3 loop and APH coiled-coil. The sequence PYY at the tip of CDR3 shields the hydrophobic interface between two APH chains. Energetically strong interactions are mediated by APH sidechain of residues Q21 and K25 at positions *c* and *g*, respectively, which interact with main chain CDR3 atoms. (***C***) The non-CDR residues of Nb49 harbored on the beta-strands C, C' and C'' completely bury the $W24_f$ side chain of APH. (***D***) The Nb49-APH hotspot interaction is mediated by the APH residue $R29_d$ which is involved in a salt bridge with the residues from the CDR3 loop and a cation-pi interaction with the Tyr from the CDR1 loop.

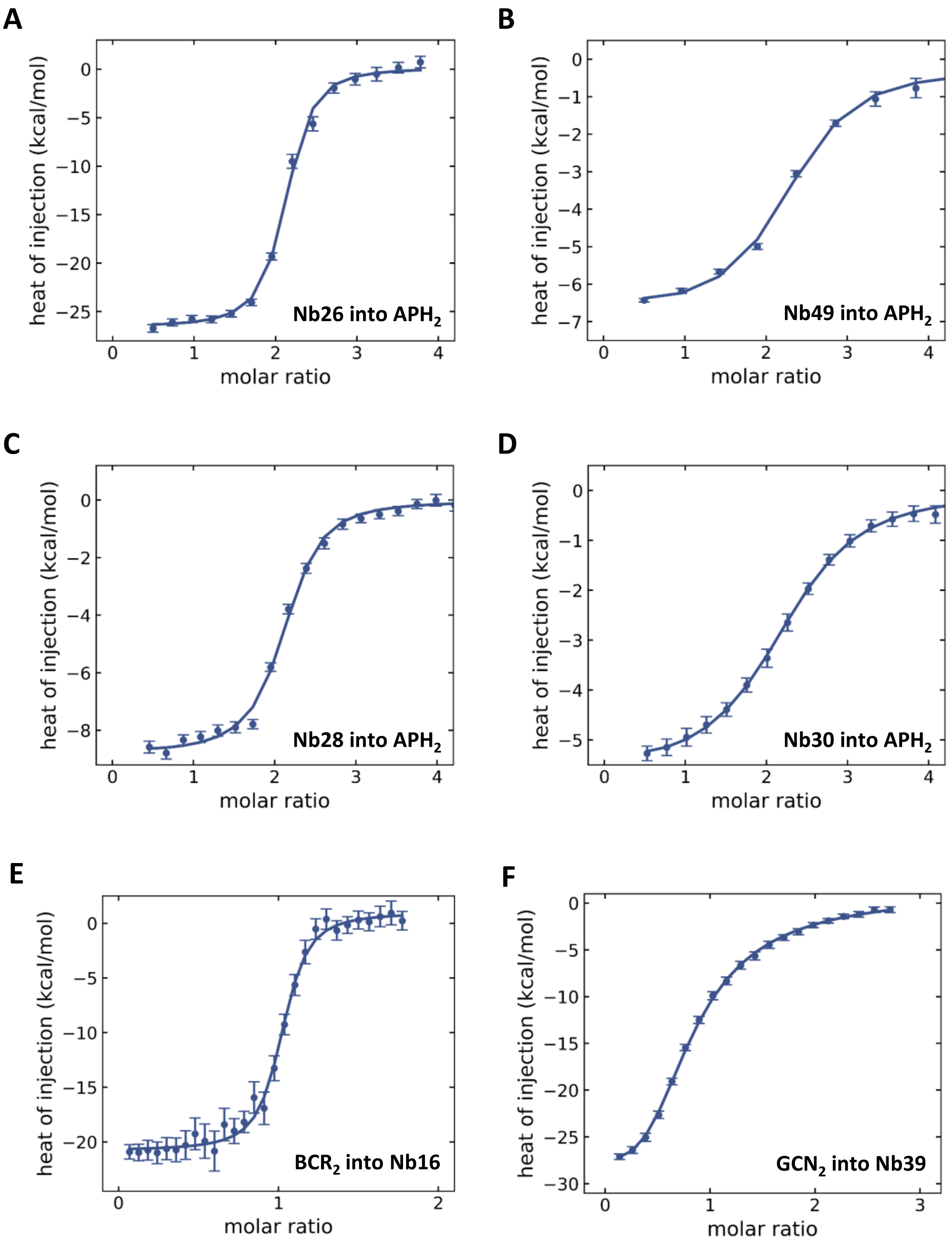

Fig. S5. Interactions between nanobodies and coiled-coils measured by ITC. The cell contained a solution of peptide pairs that assembles into coiled-coils APH_2 (panel ***A***-***D***) or GCN_2 (panel ***E***) at concentrations around 10 µM. This solution was titrated with the nanobodies Nb26 (***A***), Nb49 (***B***), Nb28 (***C***), Nb30 (***D***), Nb16 (***E***) or Nb39 (***F***). The data (circles) were described using an independent binding sites model (solid line), which yielded binding parameters listed in Table S2. All titrations were performed at 25 °C as described in *SI Materials and Methods*. Bars indicate measurement errors of the data as obtained by integrating thermograms using NITPIC. At these concentrations no binding of Nb34 to P5-P6 CC was observed.

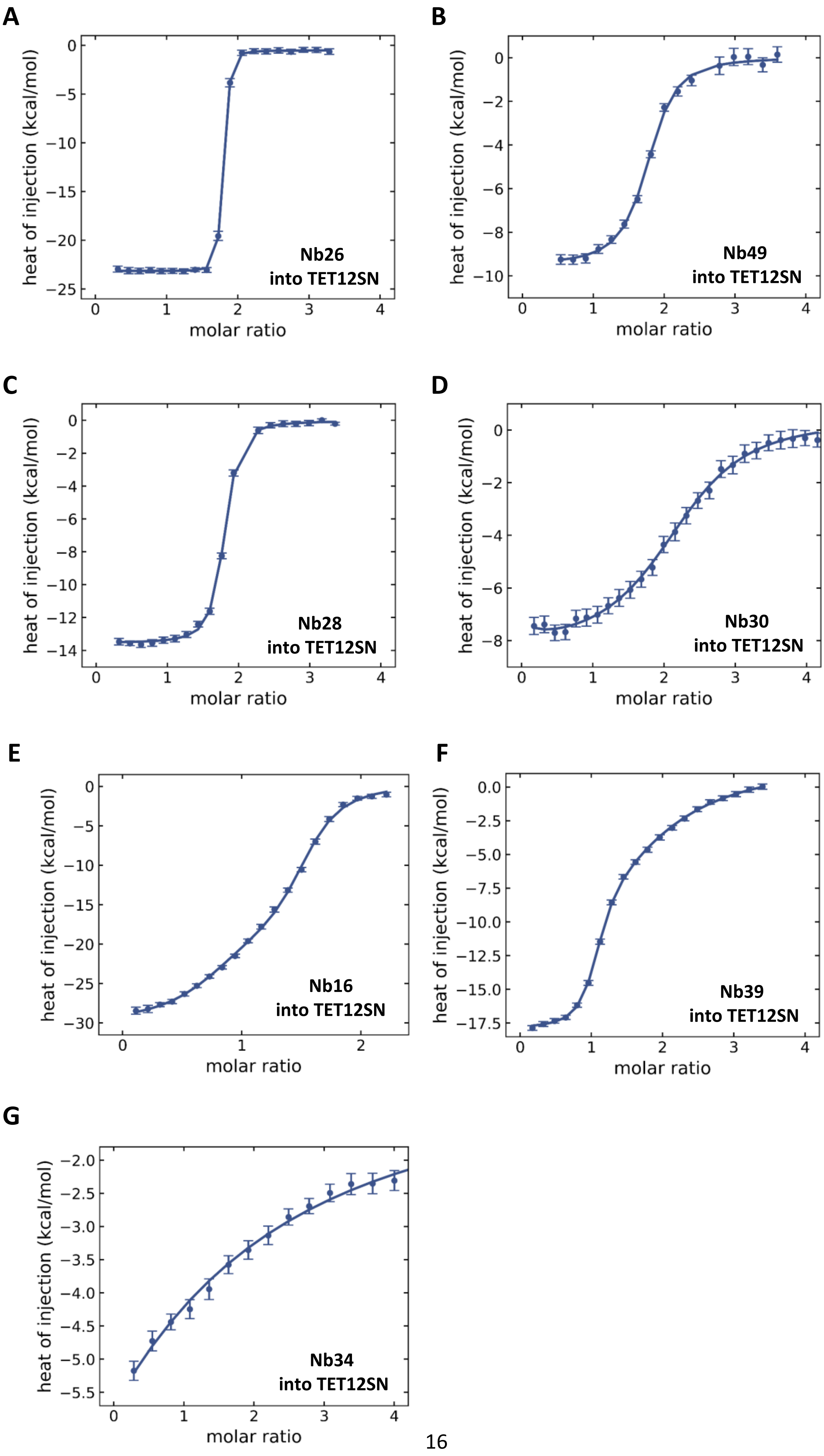

A
heat of injection (kcal/mol)
molar ratio
Nb26
into TET12SN
B
heat of injection (kcal/mol)
molar ratio
Nb49
into TET12SN
C
heat of injection (kcal/mol)
molar ratio
Nb28
into TET12SN
D
heat of injection (kcal/mol)
molar ratio
Nb30
into TET12SN
E
heat of injection (kcal/mol)
molar ratio
Nb16
into TET12SN
F
heat of injection (kcal/mol)
molar ratio
Nb39
into TET12SN
G
heat of injection (kcal/mol)
molar ratio
Nb34
into TET12SN

Fig. S6. Interactions between nanobodies and TET12SN tetrahedron measured by ITC. The cell contained a solution of TET12SN at 5 μM concentration, which was titrated with the nanobodies Nb26 (***A***), Nb49 (***B***), Nb28 (***C***), Nb30 (***D***), Nb16 (***E***), Nb39 (***F***) or Nb34 (***G***). The data (circles) were described using an independent binding sites model (solid line) yielding binding parameters which are reported in Table S2. Bars indicate data measurement errors as obtained from integration of thermograms using NITPIC. All titrations were performed at 25 °C as described at *SI Materials and Methods*.

A

TET12SN(W24A)$_1$:

APHshSN(W24A)-P3SN-BCRSN-GCNshSN-APHshSN-P7SN-GCNshSN-P4SN-P5SN-P8SN-BCRSN-P6SN

TET12SN(W24A)$_5$:

APHshSN-P3SN-BCRSN-GCNshSN-APHshSN(W24A)-P7SN-GCNshSN-P4SN-P5SN-P8SN-BCRSN-P6SN

TET12SN(W24A)$_{1,5}$:

APHshSN(W24A)-P3SN-BCRSN-GCNshSN-APHshSN(W24A)-P7SN-GCNshSN-P4SN-P5SN-P8SN-BCRSN-P6SN

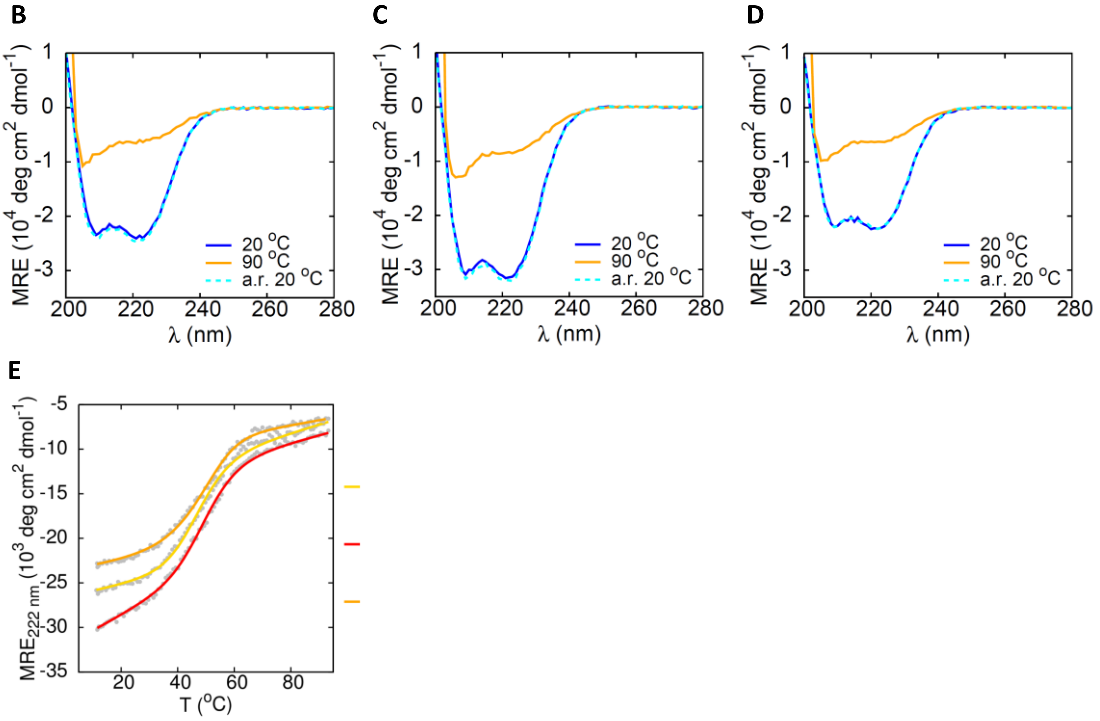

Fig. S7. Secondary structure and thermal stability of the tetrahedron TET12SN variants partially or completely inactivated nanobody binding site in the APH$_2$ module. (***A***) Segments (segment full name/segment name: APHshSN/APH, BCRSN/BCR, GCNshSN/GCN, P3SN/P3, P4SN/P4, P5SN/P5, P6SN/P6, P7SN/P7, P8SN/P8) in TET12SN variants TET12SN(W24A)$_{1,}$ TET12SN(W24A)$_5$ and TET12SN(W24A)$_{1,5}$. (***B-D***) Circular dichroism (CD) spectra at 20 °C, 90 °C and after rapid cooling from 90 °C to 20 °C (refolded protein) of the proteins TET12SN(W24A)$_1$ (***B***), TET12SN(W24A)$_5$ (***C***) and TET12SN(W24A)$_{1,5}$ (***D***). (***E***) Temperature traces of the CD signal at 222 nm, together with their thermodynamic model fits: yellow curve for TET12SN(W24A)$_1$ (T_m = 47 °C), red curve for TET12SN(W24A)$_5$ (T_m = 50 °C), orange curve for TET12SN(W24A)$_{1,5}$ (T_m = 49 °C). All experiments were performed in 20 mM Tris-HCl pH 7.5 and 150 mM NaCl.

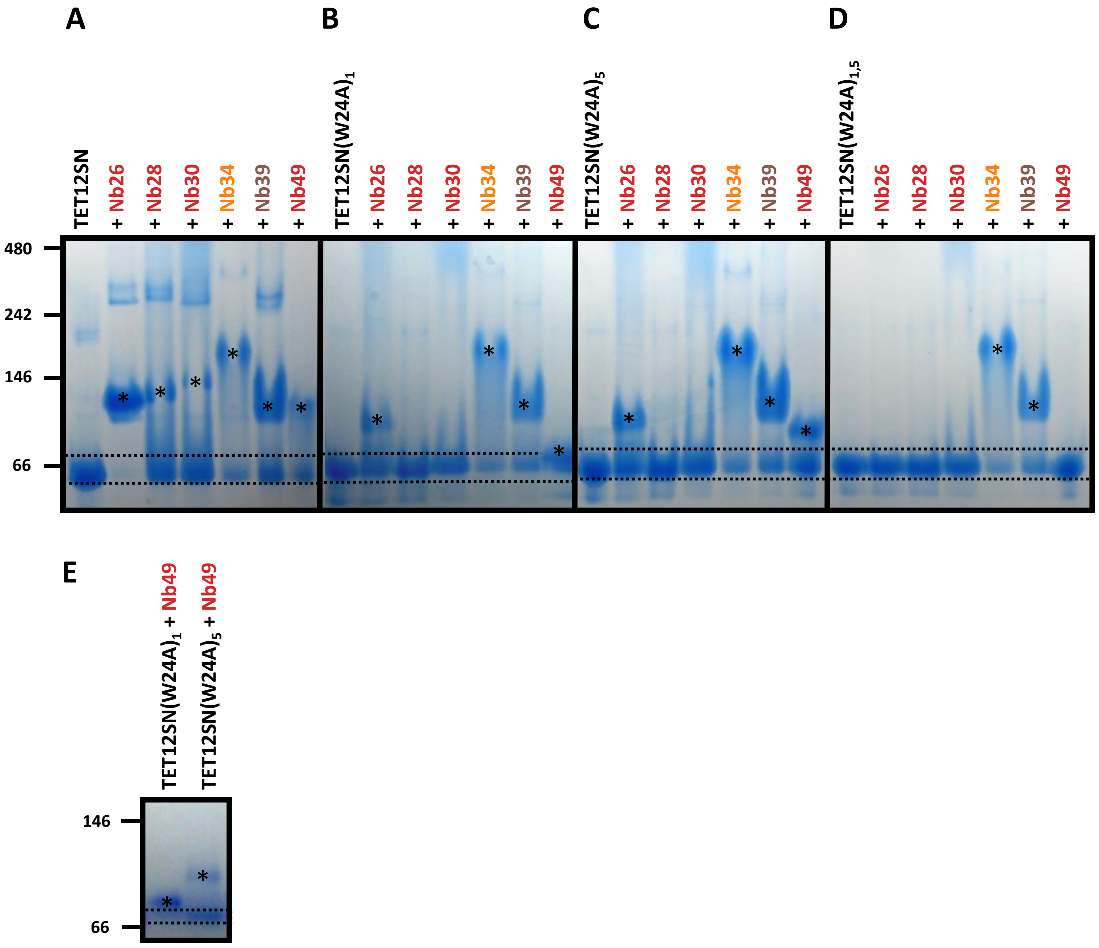

Fig. S8. Analysis of nanobody binding to the tetrahedron TET12SN variants with partially or completely inactivated nanobody binding site in the APH$_2$ module. The target proteins (5 µM) were incubated with nanobodies in tenfold molar excess overnight in 20 mM Tris-HCl pH 7.5 and 150 mM NaCl at 4 °C. Binding of nanobodies Nb26, Nb49, Nb28, Nb30, Nb39 or Nb34 to TET12SN (***A***), TET12SN(W24)$_1$ (***B***), TET12SN(W24A)$_5$ (***C***) or TET12SN(W24A)$_{1,5}$ (***D***) was analyzed on 8 % (w/v) native gels (pH 8.8) at 130 V for 2 hours and stained with InstantBlue^{TM}. (***E***) Additional presentation of the position of a complex composed of Nb49 and TET12SN(W24A)$_1$ or Nb49 and TET12SN(W24A)$_5$ on an 8 % (w/v) native gel (pH 8.8). The sizes of the proteins (in kDa) in the unstained protein standard NativeMark^{TM} are shown on the left side. The positions of the CC protein origami tetrahedra themselves (TET12SN (53.4 kDa, pI = 4.70), TET12SN(W24A)$_1$ (53.7 kDa, pI = 4.70), TET12SN(W24A)$_5$ (53.7 kDa, pI = 4.70), TET12SN(W24A)$_{1,5}$ (53.4 kDa, pI = 4.70)) are marked by two dashed lines. Positions of the complexes nanobody-tetrahedron are marked with asterisks. The free nanobodies Nb26 (14.5 kDa, pI = 8.58), Nb28 (13.6 kDa, pI = 8.40) and Nb30 (13.5 kDa, pI = 8.02) migrate slowly, their bands are above 480 kDa. Due to their high isoelectric point, the free Nb49 (13.8 kDa, pI = 9.39), Nb39 (14.0 kDa, pI = 9.01) and Nb34 (13.0 kDa, pI = 9.51) are too positively charged to run into the native gel. All experiments were performed at least twice.

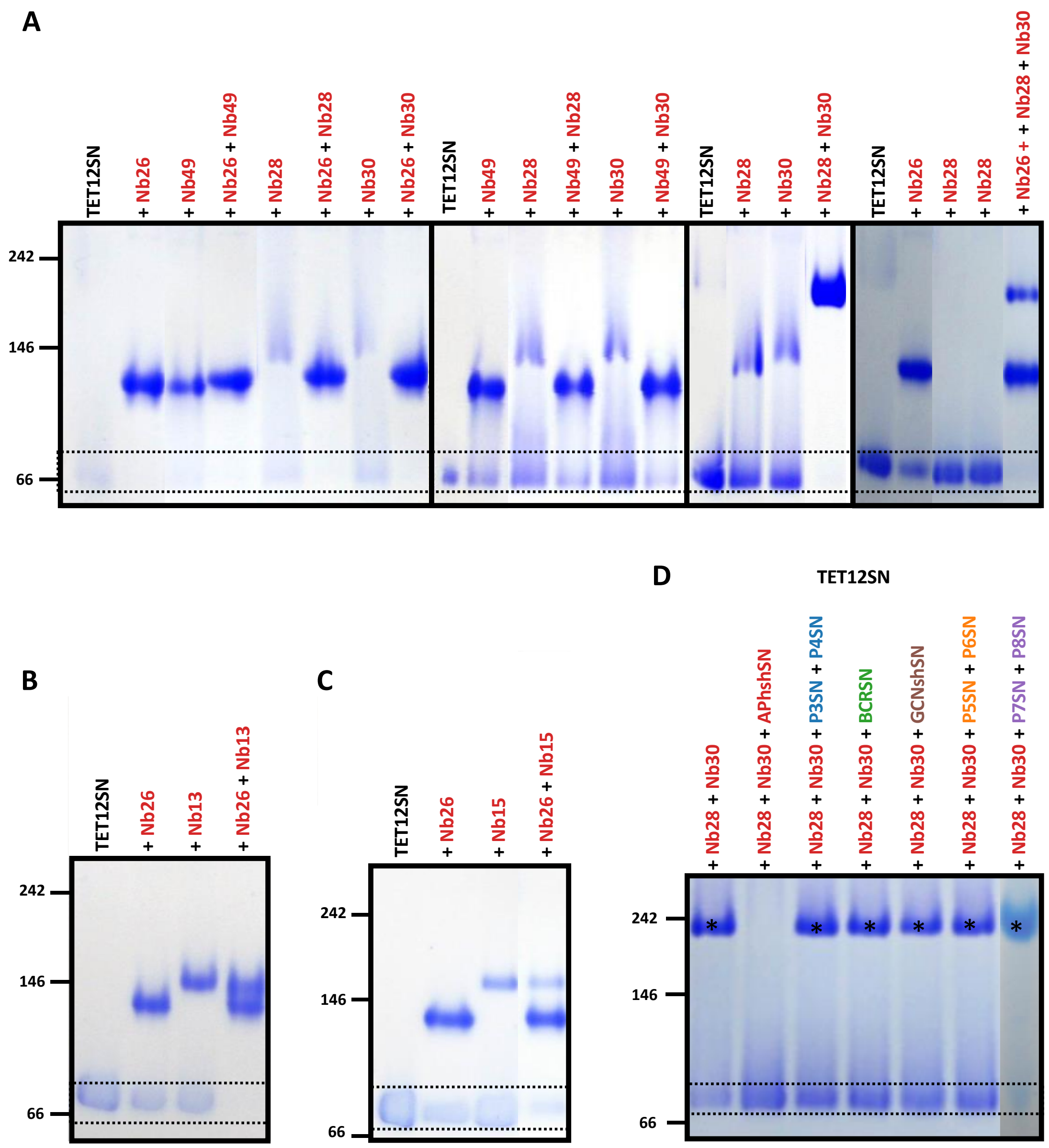

Fig. S9. **Identification of concomitant nanobody binding to the APH_2 module in the tetrahedron TET12SN.** (***A-C***) We incubated TET12SN (5 µM) with different combinations of characterized nanobodies targeting APH_2 module in tenfold molar excess each overnight in 20 mM Tris-HCl pH 7.5 and 150 mM NaCl at 4 °C. In panel ***A*** are shown results of the experiments with the combinations of characterized nanobodies Nb26, Nb28, Nb30 and Nb49. In panel ***B*** is shown a result of the experiment, where we added to the tetrahedron concomitantly two nanobodies belonging to the same nanobody group (Nb26 and Nb13 from Group 1). In panel ***C*** is shown a result of the experiment, where we added to the tetrahedron concomitantly Nb26 from Group 1 and Nb15 from Group 2 which also targets APH_2 module. (***D***) TET12SN (5 µM) was incubated with the nanobodies Nb28 and Nb30 in tenfold molar excess each and a CC peptide pair (a peptide APHshSN, BCRSN or GCNshSN forming a homodimer (APH_2, BCR_2, or GCN_2) or a pair of peptides P3SN and P4SN, P5SN and P6SN or P7SN and P8SN forming a heterodimer (P3-P4, P5-P6 or P7-P8)) in 25-fold molar excess overnight in 20 mM Tris-HCl pH 7.5 and 150 mM NaCl at 4 °C. Binding of nanobodies to TET12SN was for all described experiments analyzed on 8 % (w/v) native gels (pH 8.8) at 130 V for 2 hours. Gels were stained in Coomassie staining solution or with InstantBlue™.

The sizes of the proteins (in kDa) in the unstained protein standard NativeMark^{TM} are marked on the left side. The positions of the TET12SN itself (53.8 kDa, pI = 4.70) are marked by two dashed lines. The positions of the complexes consisting of TET12SN and nanobodies are marked with asterisks. The free nanobodies Nb26 (14.5 kDa, pI = 8.58), Nb28 (13.6 kDa, pI = 8.40) and Nb30 (13.5 kDa, pI = 8.02) migrate slowly, their bands are not shown in the figure. Due to their high isoelectric point, the free Nb49 (13.8 kDa, pI = 9.39) and Nb15 (14.2 kDa, pI = 8.98) are too positively charged to run into the native gel. Data are representative of two independent experiments.

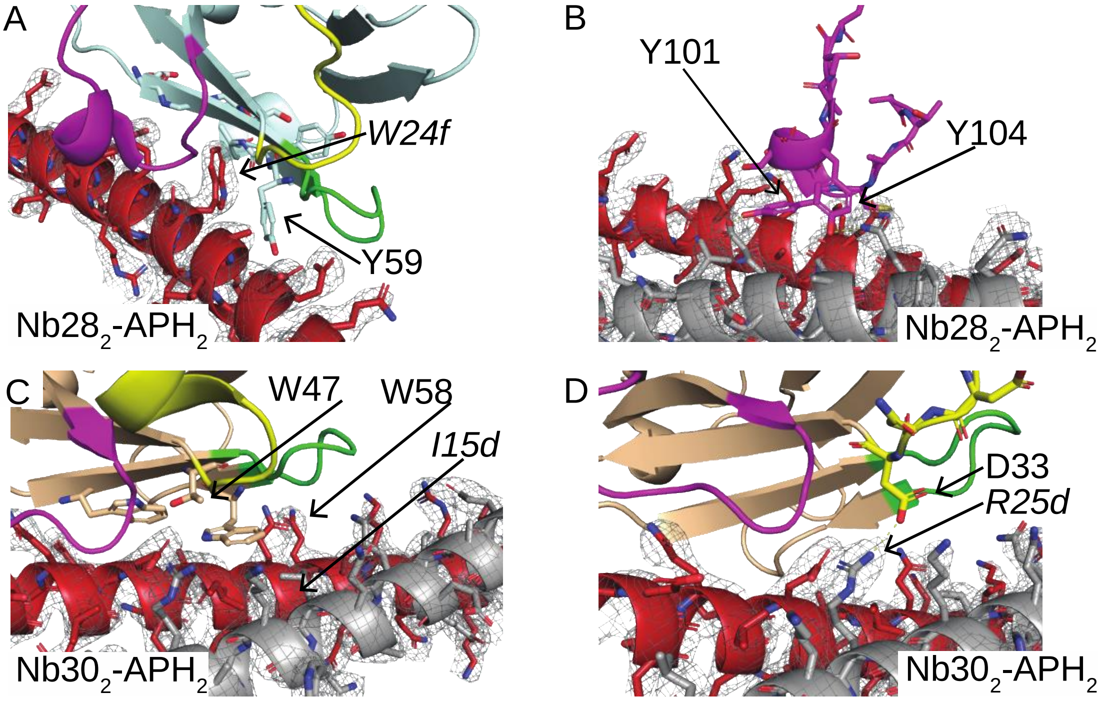

Fig. S10. Interactions between nanobody Nb28 (cyan) or nanobody Nb30 (light brown) and the antiparallel APH coiled-coil (chain A- red, chain B-gray) in the $Nb28_2$-$Nb30_2$-APH_2 complex. Nanobody residues are labeled in regular font, APH residues are labeled in italic according to the sequence number and its position of the heptad repeat (*a-f* for chain A or *a'-f'* for chain B). Electron density (2Fo-Fc map are contoured at 1 sigma) is shown only for coiled-coil for clarity. CDR loops are shown in yellow (CDR1), green (CDR2) and pink (CDR3). (***A***) The Y59 from the nanobody forms a stacking interaction with the APH $W24_f$ side chain thereby shielding it from the solvent. Second APH chain is omitted for clarity. (***B***) Long CDR3 forms favorable interactions with residues at positions *e* and *f*, while it also extends over the second APH chain to shield the hydrophobic core of CC (residues at position *a*) using Y101 and Y105, similarly as observed for the tip of CDR3 from Nb26. Only CDR3 residues are shown for clarity. (***C***) The non-CDR residues of Nb49 particularly W47 on strand C' and W58 on strand C'' shield the hydrophobic residues at positions *d* ($I15_d$ is shown) in the APH_2 core. (***D***) The interactions between CDR loops of Nb30 and APH are very scarce. One such interaction is the salt bridge between D33 on CDR1 and R25 at position *d'* on the other APH chain.

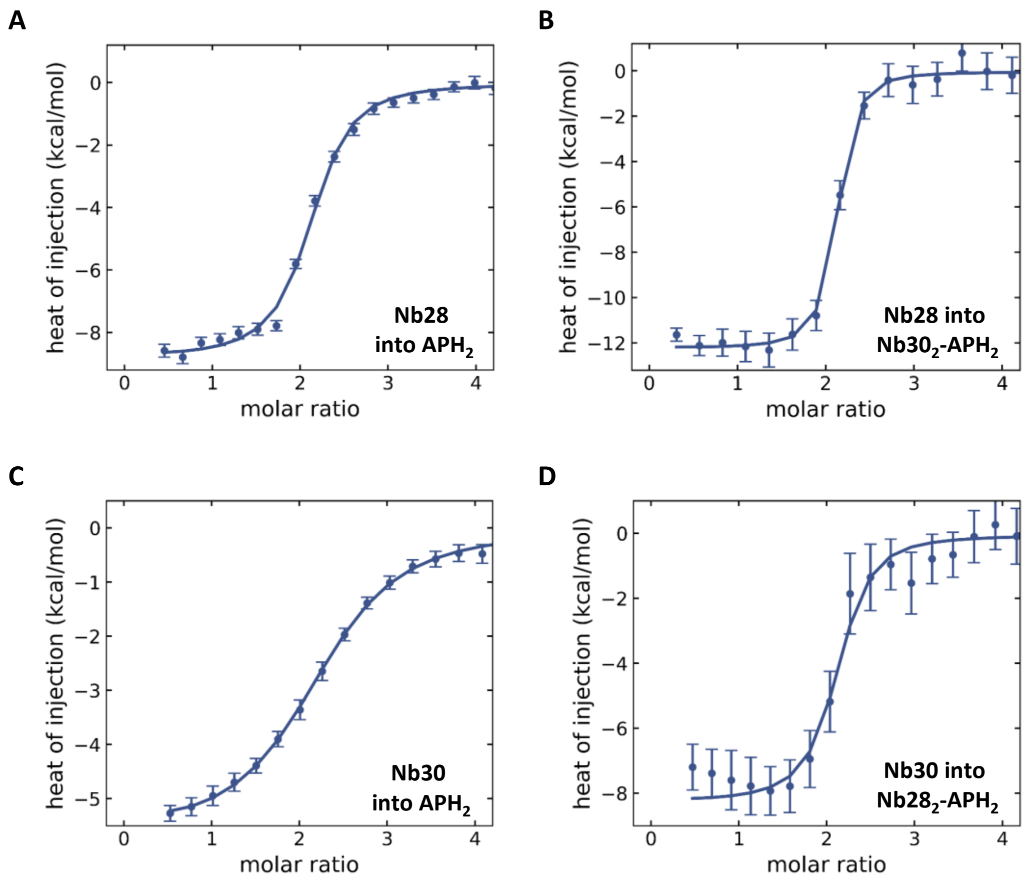

Fig. S11. Allosteric coupling between Nb28 and Nb30 binding to the isolated APH peptide pair. ITC titrations show that the binding affinity of Nb28 to APH_2 (panel ***A***) increases significantly, when Nb28 is titrated into the $Nb30_2$-APH_2 complex (panel ***B***). The same effect is observed, when the ternary $Nb28_2$-$Nb30_2$-APH_2 complex is formed in a different order. The affinity of Nb30 to APH_2 (panel ***C***) is increased, when Nb30 is titrated into $Nb28_2$-APH_2 complex (panel ***D***). All preformed nanobody-TET12SN complexes were obtained by performing a titration of the nanobody into TET12SN, followed by a second titration to produce a ternary complex. The data (circles) were described using an independent binding sites model (solid line) yielding binding parameters which are reported in Table S2. Bars indicate the data measurement errors as obtained from integration of thermograms using NITPIC. All titrations were performed at 25 °C as described at *SI Materials and Methods*.

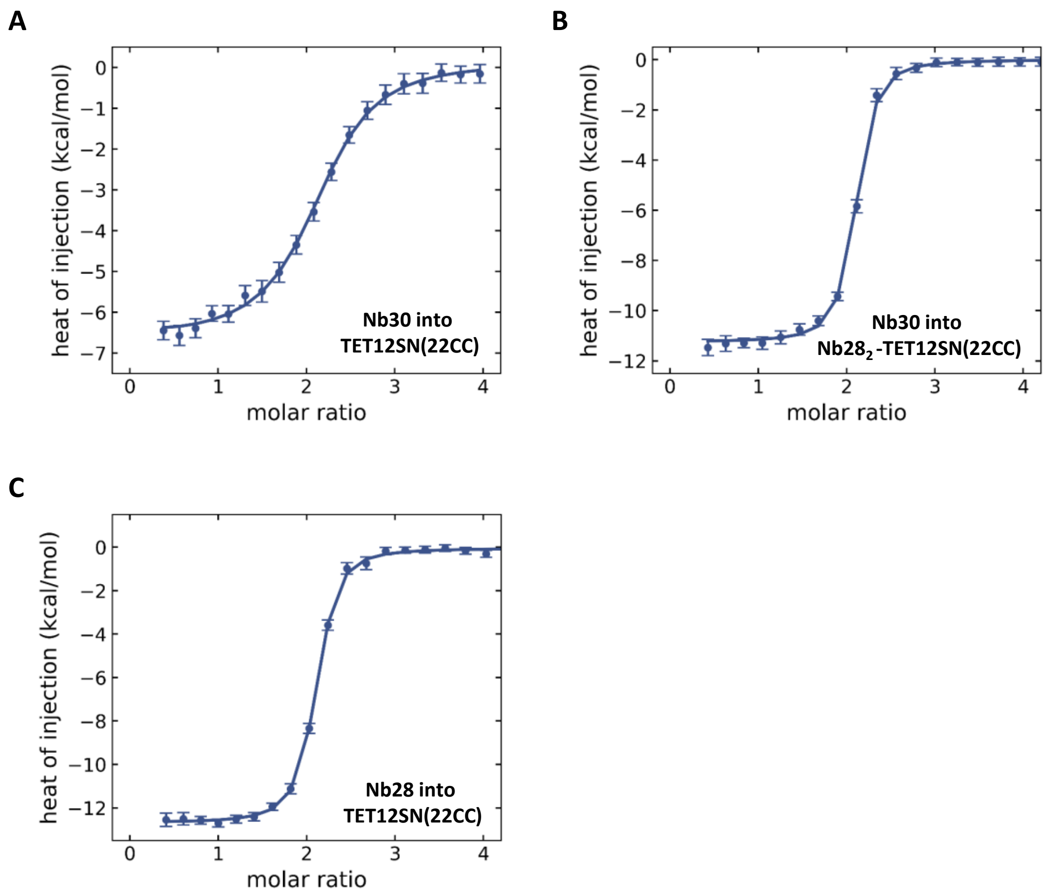

Fig. S12. Allosteric coupling between Nb28 and Nb30 binding to the TET12SN(22CC) tetrahedron variant. ITC titrations show that the binding affinity of Nb30 to TET12SN(22CC) (***A***) increases significantly, when Nb30 is titrated into the preformed Nb28$_2$-TET12SN(22CC) complex (***B***). The Nb28$_2$-TET12SN(22CC) complex was formed by titration of Nb28 into TET12SN(22CC) to molar ratio = 4, followed by a titration shown in panel B. Panel ***C*** shows the ITC titration of Nb28 into TET12SN(22CC). The data (circles) were described using an independent binding sites model (solid line) yielding binding parameters which are reported in Table S2. Bars indicate data measurement errors as obtained from integrating thermograms using NITPIC. All titrations were performed at 25 °C as described at *SI Materials and Methods*.

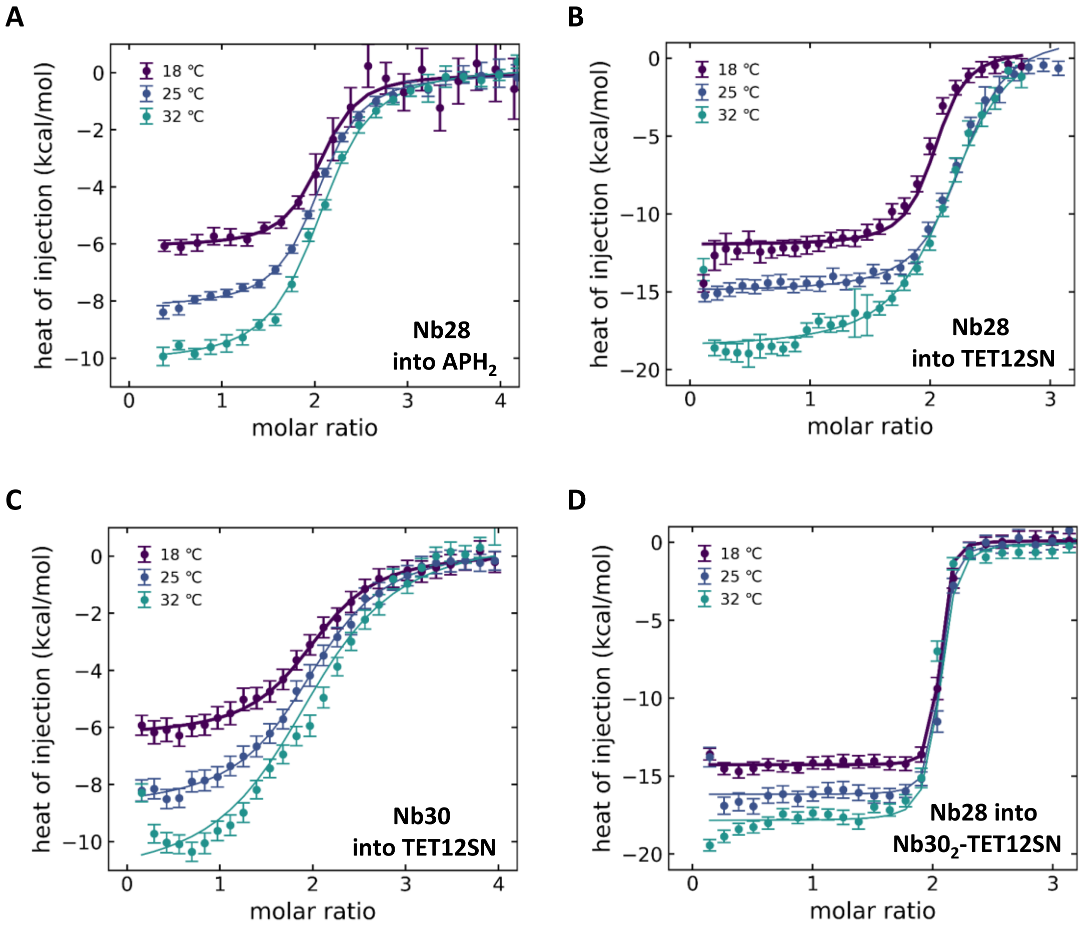

Fig. S13. Determination of the heat capacity contributions from temperature-dependent ITC measurements. ITC titrations performed at 18, 25 and 32 °C were used to determine the heat capacity contributions of Nb28 binding to APH (***A***), Nb28 binding to TET12SN (***B***), Nb30 binding to TETSN12 (***C***) and Nb28 binding to Nb30$_2$-TET12SN complex (***D***). The data (circles) were described using a global model with the assumption of independent binding sites model and a temperature independent heat capacity contribution (sloid lines). The model parameters are reported in Table S2.

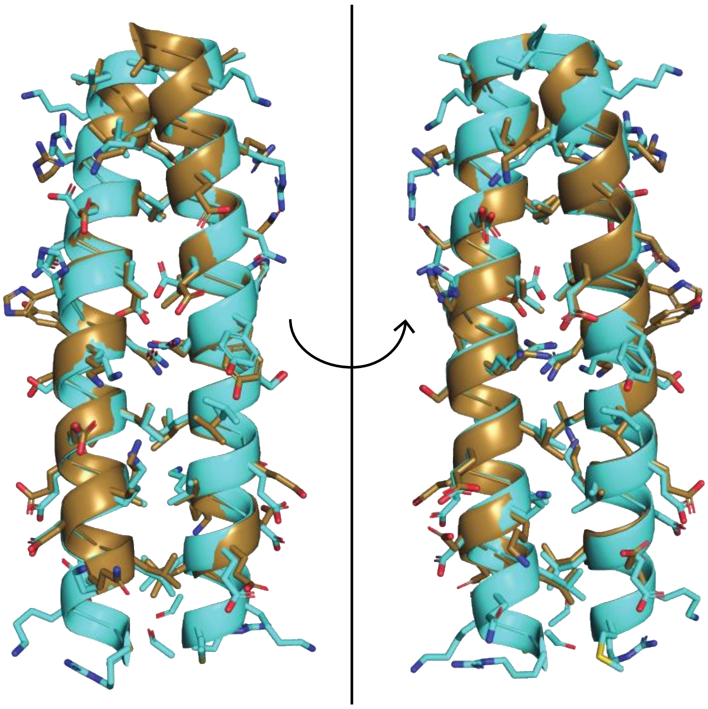

Fig. S14. Superposition of the GCN_2 in the complex Nb39-GCN_2 with the isolated GCN coiled-coil. Two sides of GCN_2 molecule as observed in the Nb39-GCN_2 co-crystal structure (gold color) or as isolated GCN coiled-coil (PDB: 2zta, shown in blue). The isolated GCN is five residues longer compared to one used for the co-crystallization with Nb39. It contains three additional residues at the N-terminus (sequence RMK) and two additional residues at C-terminus (sequence VG). Globally the structures of both GCN coiled-coils are essentially identical, indicating that Nb39 binding does not perturb the structure of GCN.

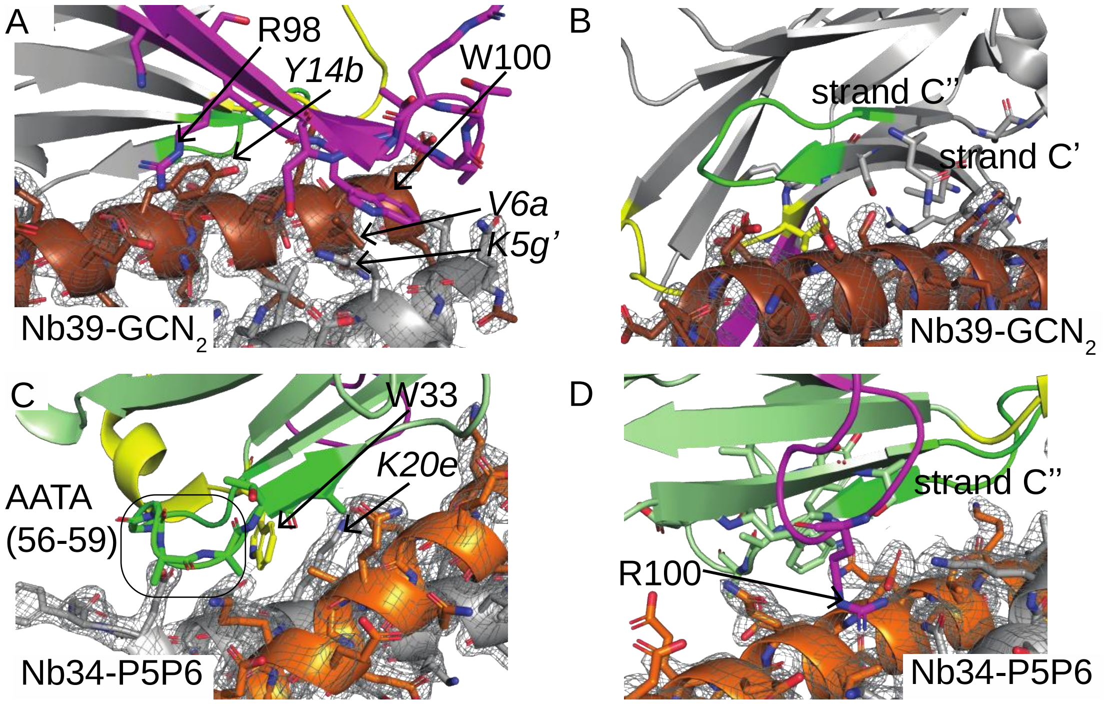

Fig. S15. Interactions between nanobody Nb39 (gray) and parallel GCN coiled-coil (chain A-brown, chain B-gray) and those between nanobody Nb34 (pale green) and parallel P5-P6 coiled-coil (chain A-orange, chain B-gray) in the Nb39-GCN$_2$ or Nb34-P5-P6 complex. Nanobody residues are labeled in regular font, APH residues are labeled in italic according to the sequence number and its position of the heptad repeat (*a-f* for chain A or *a'-f'* for chain B). Electron density (2Fo-Fc map are contoured at 1 sigma) is shown only for coiled-coil for clarity. CDR loops are shown in yellow (CDR1), green (CDR2) and pink (CDR3). (***A***) The CDR3 loop of Nb39 extends over both GCN chains and shields hydrophobic residues V6$_a$ and L9$_d$ in the core of GCN$_2$. Furthermore, two cation-pi interactions stabilize the complex: the interaction between CDR3 R98 and Y14$_b$ and that between CDR3 W100 and K5$_{g'}$ side chain on the other GCN molecule. (***B***) Framework beta-strands C' and C'' are shown which run parallel to the GCN chain and a number of interactions between GCN and Nb39 non-CDR residues. (***C***) The tip of CDR2 loop from Nb34 contains a stretch of alanine residues (56-AATA-59) that shield the hydrophobic core between both GCN chains. Additionally, the W33 on CDR1 form a cation-pi interaction with the GCN K20 at position *e*. (***D***) The CDR3 loop from Nb34 harbors R100, which is involved in a network of interactions with residues on both P5 and P6 chains (N16$_a$, Q11$_{c'}$ and E15$_{g'}$). Residues from the nanobody framework (strands C' and C'') are positioned parallel to the GCN chain and establish several polar interactions.

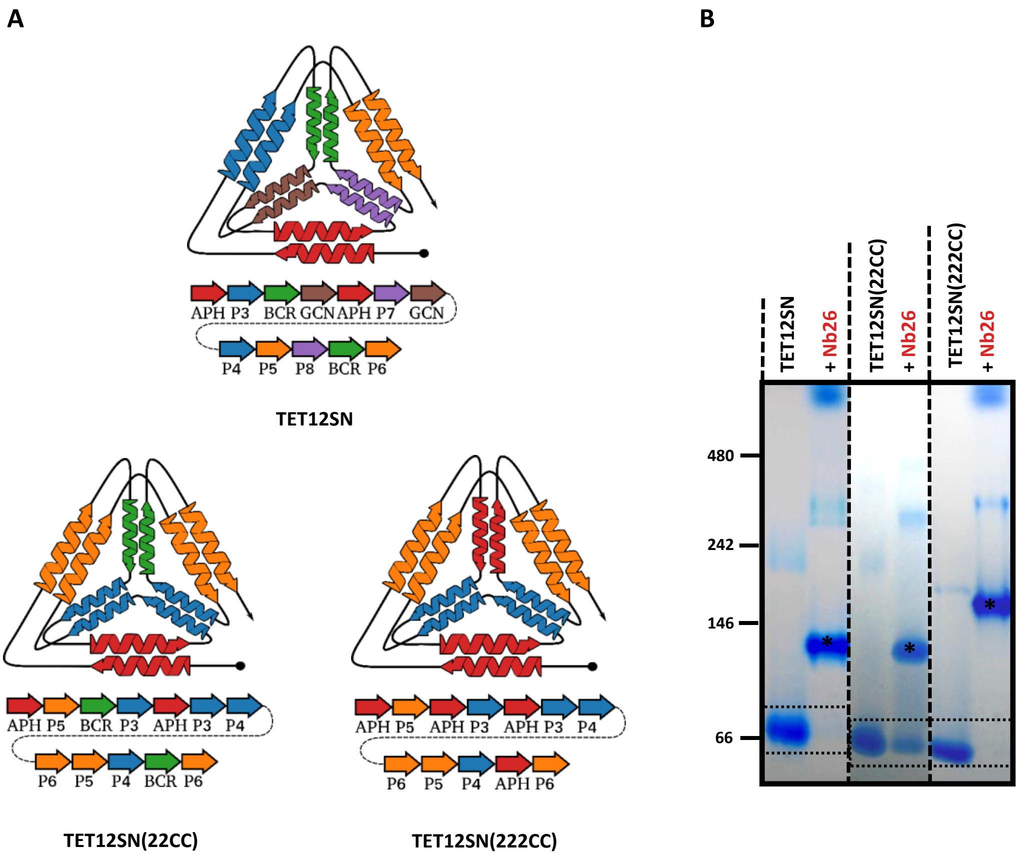

Fig. S16. Analysis of nanobody binding to the tetrahedron TET12SN variants with different repetitions of the target module APH$_2$. The target protein (TET12SN, TET12SN(22CC) or TET12SN(222CC)) (***A***) at 5 µM concentration was incubated with the nanobody Nb26 in fivefold molar excess overnight in 20 mM Tris-HCl pH 7.5 and 150 mM NaCl at 4 °C. (***B***) The binding of Nb26 to the proteins was analyzed on 8 % (w/v) native gels (pH 8.8) at 130 V for 2 hours. Gels were stained with InstantBlue^{TM}. The sizes of the proteins (in kDa) in the unstained protein standard NativeMark^{TM} are marked on the left side of the gel. The positions of the TET12SN (53.4 kDa, pI = 4.70), the TET12SN(22CC) (54.8 kDa, pI = 4.58) and the TET12SN(22CC) (55.2 kDa, pI = 4.56) themselves are indicated by two dashed lines. Positions of the complexes composed of Nb26 and a tetrahedron are marked with asterisks. The free nanobody Nb26 (14.5 kDa, pI = 8.58) migrates slowly, its bands are above 480 kDa. The data are representative of two independent experiments.

A

MBP-BIP18APH

MBP-P1SN-APHshSN-P7SN-P2SN-P3SN-P9SN-APHshSN-P4SN-P5SN-P1SN-APHshSN-P8SN-P2SN-P3SN-P10SN-APHshSN-P4SN-P6SN

**MG*HHHHHHHH*MKIEEGKLVIWINGDKGYNGLAEVGKKFEKDTGIKVTVEHPDKLEEKFPQVAATGDGPDIIFWAHDRFGGYAQSGL
LAEITPDKAFQDKLYPFTWDAVRYNGKLIAYPIAVEALSLIYNKDLLPNPPKTWEEIPALDKELKAKGKSALMFNLQEPYFTWPLIAA
DGGYAFKYENGKYDIKDVGVDNAGAKAGLTFLVDLIKNKHMNADTDYSIAEAAFNKGETAMTINGPWAWSNIDTSKVNYGVTVLPT
FKGQPSKPFVGVLSAGINAASPNKELAKEFLENYLLTDEGLEAVNKDKPLGAVALKSYEEELAKDPRIAATMENAQKGEIMPNIPQM
SAFWYAVRTAVINAASGRQTVDEALKDAQTRITKGGSGG<u>ENLYFQG</u>SGGHMSPEDEIRQLEQENSQLERENQRLEQEIYQLERGS
GPGLEEELKQLEEELQAIEEQLAQLQWKAQARKEKLAQLKEKLGSGPGSPEDEIQQLEEKNSQLKQEISQLEEKNQELKYGSGPG
SPEDKIEELKEKNSQLKEKNEELKQKIYELKEGSGPGSPEDEIQQLEEEISQLEQKNSELKEKNQELKYGSGPGSPEDENQSLEQK
NSQLKQEISQLEQEIQQLEYGSGPGLEEELKQLEEELQAIEEQLAQLQWKAQARKEKLAQLKEKLGSGPGSPEDKISQLKEKIQQL
KQENQQLEEENSQLEYGSGPGSPEDENSQLEEKISQLKQKNSELKEEIQQLEYGSGPGSPEDEIRQLEQENSQLERENQRLEQEIY
QLERGSGPGLEEELKQLEEELQAIEEQLAQLQWKAQARKEKLAQLKEKLGSGPGSPEDKISELKEENQQLEQKIQQLKEENSQLE
YGSGPGSPEDKIEELKEKNSQLKEKNEELKQKIYELKEGSGPGSPEDEIQQLEEEISQLEQKNSELKEKNQELKYGSGPGSPEDKN
SQLKEENSQLEEKIEQLKEKIQELKYGSGPGLEEELKQLEEELQAIEEQLAQLQWKAQARKEKLAQLKEKLGSGPGSPEDKISQLK
EKIQQLKQENQQLEEENSQLEYGSGPGSPEDKNSELKEEIQQLEEENQQLEEKISELKYLELE**

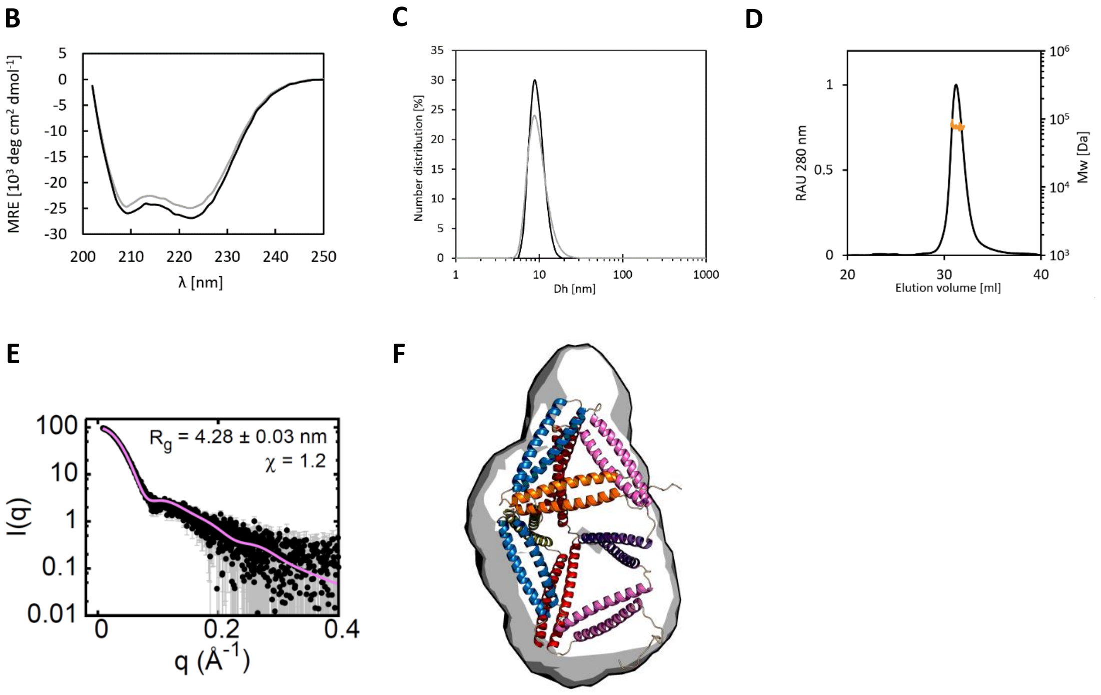

Fig. S17. **Biophysical characterization of the trigonal bipyramid BIP18APH.** (***A***) Amino acid sequence of the fusion protein MBP-BIP18APH, where *HHHHHHHH* means octahistidine tag, a sequence <u>ENLYFQG</u> TEV protease cleavage site and GSGPG linkers between segments. Cleavage with TEV protease yields MPB (maltose binding protein) and BIP18APH. (***B***) CD spectra of BIP18APH at 20 °C (black) and 20 °C after denaturation and rapid refolding (gray). (***C***) Numeric distribution of hydrodynamic diameter (Dh) of BIP18APH calculated from batch DLS measurement before (black) and after (gray) temperature denaturation. (***D***) Monodispersity of BIP18APH was analyzed by SEC coupled to MALS. The panel represents the size-exclusion chromatogram (normalized so that major peak maximum is set to 1) with an overlay of molar masses calculated from the SEC-MALS measurement (red). (***E***) Solution SAXS analysis of the size and shape of the protein showed a good agreement between the observed scattering curve depicted with black dots with a theoretical curve, calculated from molecular models of the designed BIP18APH, represented with pink line. (***F***) *Ab initio* reconstruction leads to the bipyramidal molecular envelope.

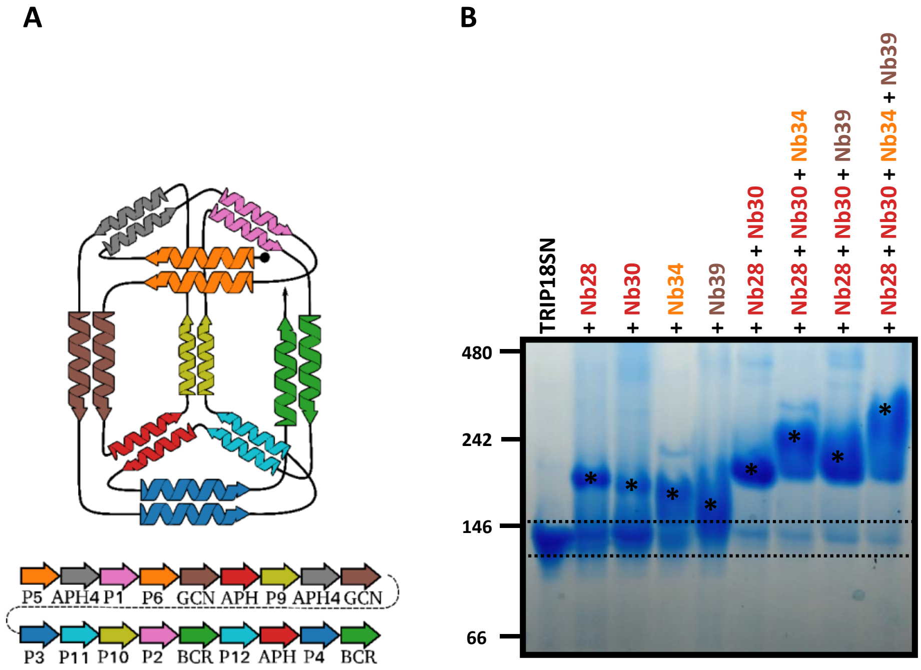

Fig. S18. Analysis of nanobody binding to the triangular prism TRIP18SN. The target protein TRIP18SN (***A***) at 5 µM concentration was incubated with nanobodies in ten or twentyfold molar excess each overnight in 20 mM Tris-HCl pH 7.5 and 150 mM NaCl at 4 °C. (***B***) The binding of nanobodies was analyzed on an 8 % (w/v) native gel (pH 8.8) at 130 V for 2 hours. Gel was stained with InstantBlue™. The sizes of the proteins (in kDa) in the unstained protein standard NativeMark™ are marked on the left side of the gel. The positions of the TRIP18SN (81.9 kDa, pI = 4.69) itself are marked by two dashed lines. The positions of the complexes of nanobodies and prism are marked with asterisks. The free nanobodies Nb28 (13.6 kDa, pI = 8.40) and Nb30 (13.5 kDa, pI = 8.02) migrate slowly, their bands are above 480 kDa. Owing to their high isoelectric point the free Nb34 (13.0 kDa, pI = 9.51) and Nb39 (14.0 kDa, pI = 9.01) are to positively charged to run into the native gel. The data are representative of two independent experiments.

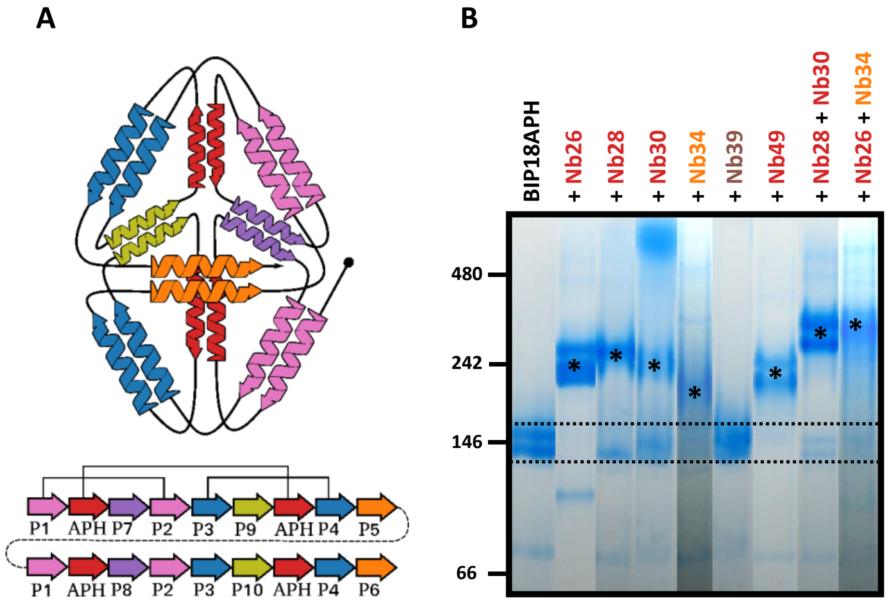

Fig. S19. Analysis of nanobody binding to the trigonal bipyramid BIP18APH. Target protein BIP18APH (***A***) at 5 µM concentration was incubated with nanobodies in five or tenfold molar excess each overnight in 20 mM Tris-HCl pH 7.5 and 150 mM NaCl at 4 °C. (***B***) Binding of nanobodies was analyzed on an 8 % (w/v) native gel (pH 8.8) at 130 V for 2 hours and stained with the stain InstantBlue™. Sizes of proteins (in kDa) in the unstained protein standard NativeMark™ are marked on the left side of the gel. The positions of the BIP18APH (80.5 kDa, pI = 4.49) itself are marked by two dashed lines. The positions of the complexes composed of nanobodies and bipyramid are marked with asterisks. The free nanobodies Nb26 (14.5 kDa, pI = 8.58), Nb28 (13.6 kDa, pI = 8.40) and Nb30 (13.5 kDa, pI = 8.02) migrate slowly, their bands are above 480 kDa. Owing to their high isoelectric point the free Nb34 (13.0 kDa, pI = 9.51), Nb39 (14.0 kDa, pI = 9.01) or Nb49 (13.8 kDa, pI = 9.39) are to positively charged to run into the native gel. The data are representative of two independent experiments.

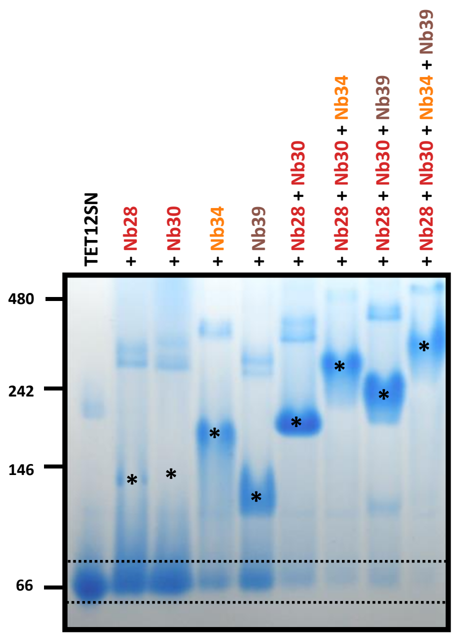

Fig. S20. Analysis of concomitant nanobody binding to the tetrahedron TET12SN. The target protein (5 µM) was incubated with nanobodies in fivefold molar excess each overnight at 4 °C in 20 mM Tris-HCl pH 7.5 and 150 mM NaCl. Concomitant binding of nanobodies to TET12SN was analyzed on an 8 % (w/v) native gel (pH 8.8) at 130 V for 2 hours. Gel was stained with InstantBlue^{TM}. The sizes of the proteins (in kDa) in the unstained protein standard NativeMark^{TM} are marked on the left side of the gel. The positions of the TET12SN itself (53.4 kDa, pI = 4.70) are indicated by two dashed lines. The positions of the complexes composed of nanobodies and TET12SN are marked with asterisks. The free nanobodies Nb26 (14.5 kDa, pI = 8.58), Nb28 (13.6 kDa, pI = 8.40) and Nb30 (13.5 kDa, pI = 8.02) migrate slowly, their bands are above 480 kDa. Owing to their high isoelectric point free Nb34 (13.0 kDa, pI = 9.51) or Nb39 (14.0 kDa, pI = 9.01) are to positively charged to run into the native gel. All experiments were performed at least two times. Binding of Nb30 as a single nanobody is invisible on this figure, but is clearly visible on e.g. Fig. S2*C*.

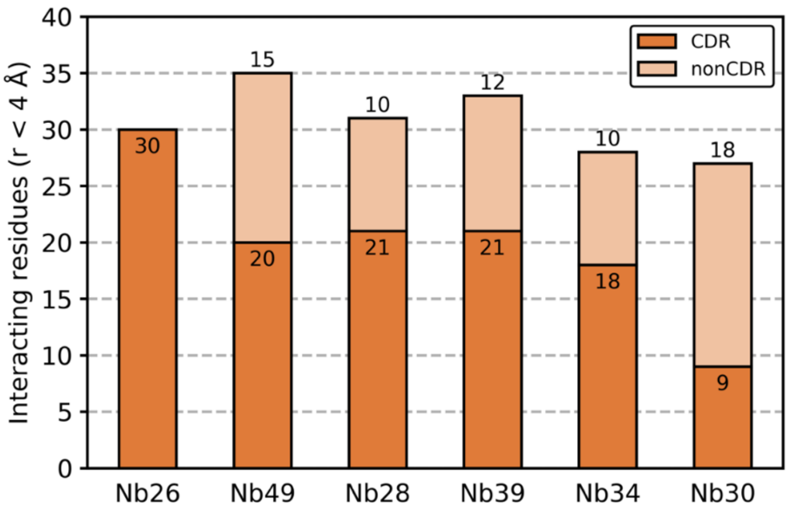

Fig. S21. Total number of interactions between nanobodies and coiled-coil dimers. The bars show number of interactions between nanobody and coiled-coil in a given complex (per epitope bases) and are further separated between interactions mediated by nanobody residues from CDR loops and other residues (non-CDR). Interaction is defined in cases, where a pair of residues (from nanobody and coiled-coil) has its atoms (excluding hydrogen atoms) closer than 4 Å.

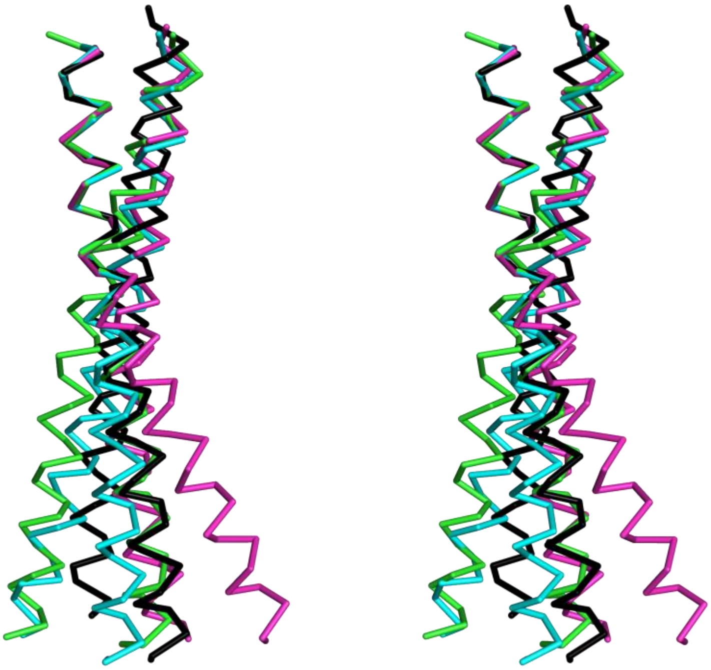

Model	Radius (Å)	Pitch (Å)	Crick´s angle	
			Chain a (deg)	Chain b (deg)
Nb26$_2$-APH$_2$	5.2	126	19	20.3
Nb49$_2$-APH$_2$	5.1	179.4	11	11
Nb28$_2$-Nb30$_2$-APH$_2$	5.1	173.3	13.1	10.8
APH$_2$ (model)	5.1	179	7.6	7.6

Fig. S22. Conformation of the APH$_2$ coiled-coil in different complexes. The stereo image shows the positions of the APH Cα atoms (ribbons) in complex with different nanobodies: Nb26 (green), Nb49 (pink) and Nb28 and Nb30 (cyan). The conformation of the unbound APH$_2$ model structure is shown as black ribbon. The backbone was built using Crick's generalized parametric equations. The side chains were subsequently packed onto the backbone using SCWRL4 (24). In order to quantitatively compare the APH$_2$ crystal structures obtained, geometric parameters of the APH$_2$ coiled-coil backbone in different conformations were calculated and are given in the table below.

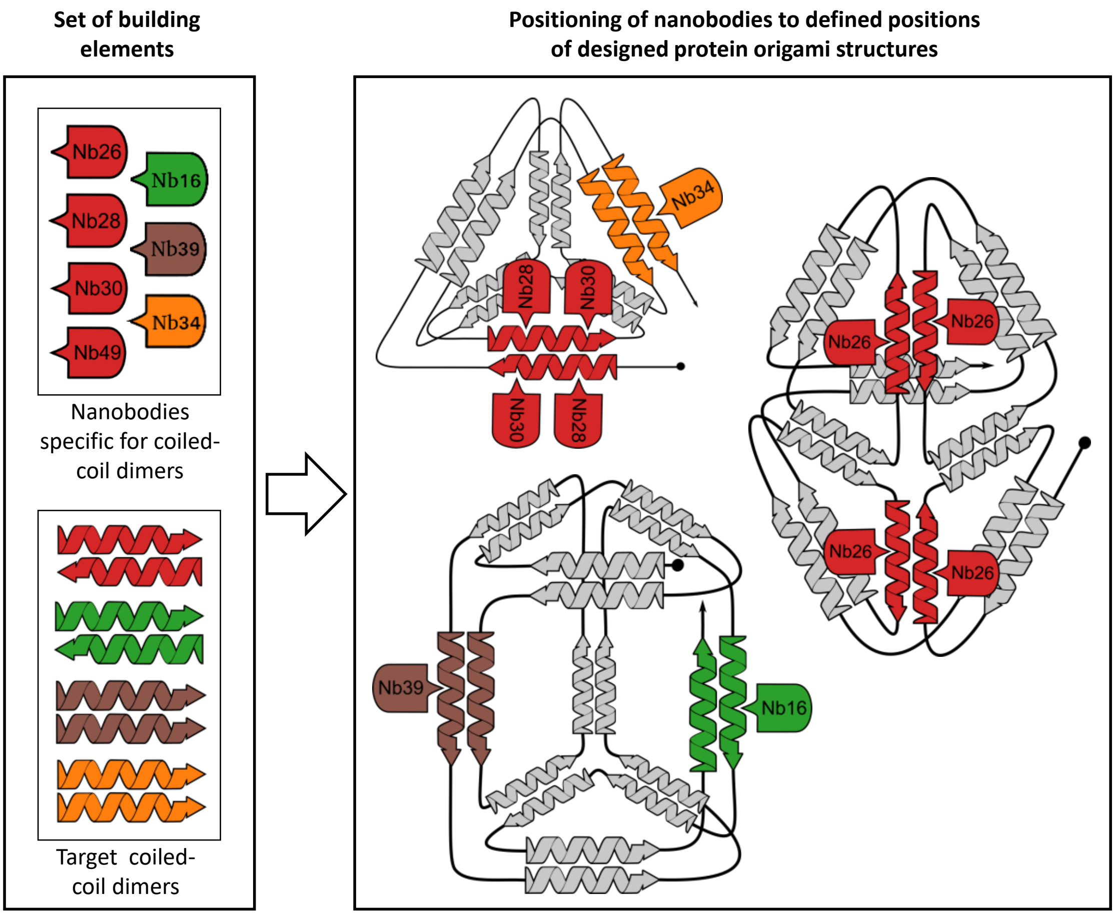

Fig. S23. A set of nanobodies specific for dimeric CC modules and target CC dimers as a new synthetic biology toolkit for functionalization of designed protein origami structures. Characterized nanobodies and specific two antiparallel and two parallel CC dimers as building elements can be combined in different ways to build diverse nanostructures, where nanobodes will be positioned at defined positions of designed protein origami structures.

Table S1. Crystal data collection and refinement statistics. Values in parenthesis refer to outer resolution shell.

Complex	*Nb26₂-APH₂*	*Nb49₂-APH₂*	*Nb39-GCN₂*	*Nb34-P5-P6*	*Nb28₂-Nb30₂-APH₂*
**PDB entry**	**7A50**	**7A48**	**7A4T**	**7A4Y**	**7A4D**
**Diffraction source**	Soleil PX1	Soleil PX1	Soleil PX2	Soleil PX2	Soleil PX1
			Data collection		
**Wavelength (Å)**	0.978570	0.978570	0.980127	0.980126	0.978570
**Resolution range (Å)[a]**	37.46 - 2.00 (2.07 - 2.00)	40.93 - 1.55 (1.60 - 1.55)	47.69 - 2.12 (2.20 - 2.12)	38.35 - 2.16 (2.23 - 2.16)	45.26 - 2.69 (2.79 - 2.69)
**Space group**	R32	C2	$P4_232$	P 3_221	$P2_12_12_1$
**Unit cell**	a, b = 198.2 Å c = 58.2 Å α, β = 90° γ = 120°	a = 94.6 Å b = 26.1 Å c = 54.3 Å α, γ = 90° β = 108.9°	a, b, c = 106.6 Å α, β, γ = 90°	a, b = 72.5 Å c = 193.8 Å α, β = 90° γ = 120°	a = 61.3 Å b = 127.5 Å c = 192.9 Å α, β, γ = 90°
**Total reflections**	60722 (9089)	56327 (8357)	459850 (68235)	357815 (57078)	502992 (77525)
**Unique reflections**	29399 (2821)	18085 (1644)	12227 (1177)	32518 (3060)	42539 (4077)
**Multiplicity**	2.07 (3.22)	3.11 (5.08)	37.61 (57.97)	11.0 (18.65)	11.82 (19.02)
**Completeness (%)**	99.52 (95.85)	96.87 (89.83)	99.93 (99.75)	99.39 (95.40)	99.48 (95.74)
**Mean I/sigma (I)**	8.07 (1.39)	7.61 (1.26)	27.45 (2.99)	14.99 (1.52)	15.52 (1.59)
**Wilson B-factor ($Å^2$)**	51.1	23.5	41.6	44.7	69.6
**R-rim**	0.222 (1.470)	0.086 (0.672)	0.121 (1.204)	0.122 (1.218)	0.144 (1.516)
**CC1/2**	99.1 (71.8)	99.6 (72.2)	100.0 (85.9)	99.9 (85.3)	99.8 (65.4)
			Model refinement		
**R-work**	0.183 (0.350)	0.188 (0.260)	0.186 (0.236)	0.198 (0.298)	0.178 (0.292)
**R-free**	0.225 (0.379)	0.216 (0.293)	0.245 (0.337)	0.228 (0.334)	0.222 (0.325)
			Number of non-hydrogen atoms		
**Total**	2766	1391	1492	3190	8395
**Protein**	2631	1295	1366	3023	8312
**Water**	123	96	99	129	61
**Other**	12	/	27	38	22
			RMSD		
**Bonds (Å)**	0.012	0.010	0.009	0.012	0.010
**Angles (°)**	1.08	1.14	0.89	1.05	1.18
			Ramachandran		
**Favored (%)**	98.8	99.4	98.2	99.5	96.4
**Outliers (%)**	0	0.00	1.8	0.5	0.1
**Clashscore**	4.6	2.7	3.3	3.0	13.1
			Average B-factor		
**Total**	70.0	32.9	53.4	56.6	75.8
**Protein**	70.3	32.3	52.5	56.3	75.8
**Ligands**	89.2	/	98.7	92.8	94.2
**Solvent**	62.3	40.7	52.6	54.1	63.4
			Crystallization		
**Condition**	0.2 M calcium chloride, 0.1 M sodium acetate pH 5, 20 % (w/v) PEG 6000	2 M ammonium sulphate, 0.1 M sodium acetate pH 4.6	0.2 M ammonium sulphate, 0.1 M sodium acetate pH 4.5, 25 % (w/v) PEG 4000	0.2 M ammonium sulphate, Bis-Tris pH 5.5, 25 % PEG 3350	0.2 M sodium chloride, 2.0 M ammonium sulphate, 0.1 M sodium cacodylate, 6.5 M
**Cryo protection**	Addition of 25 % glycerol	Addition of 25 % glycerol	3.5 M ammonium sulphate	Addition of 19 % glycerol	Addition of 25 % glycerol

Table S2. Summary of the results of ITC measurements. All parameters are reported at standard temperature 25 °C.

**Nanobody-tetrahedron TET12SN titrations**						
**Cell**	**Syringe**	**Kd (nM)**		**ΔH (kcal/mol)**		**Stoichiometry (cell:syringe)**
**TET12SN**	**Nb26**	1.8 ± 0.18		-23 ± 0.86		1:2
**TET12SN**	**Nb49**	160 ± 11		-9.6 ± 0.33		1:2
**TET12SN**	**Nb28**	39 ± 2.8		-14 ± 0.49		1:2
**TET12SN**	**Nb30**	350 ± 35		-8.8 ± 0.42		1:2
**TET12SN**	**Nb16**	9.8 ± 0.2	95 ± 6.8	-30 ± 0.04	-18 ± 0.65	1:2
**TET12SN**	**Nb39**	27 ± 4.0	2300 ± 79	-19 ± 0.69	-16 ± 0.83	1:2
**TET12SN**	**Nb34**	21000 ± 16000		- 20 ± 5.6		1:1
**Nanobody-CC peptide pair titrations**						
**Cell**	**Syringe**	**Kd (nM)**		**ΔH (kcal/mol)**		**Stoichiometry (cell:syringe)**
**APH$_2$**	**Nb26**	39 ± 2.9		-28 ± 1.0		1:2
**APH$_2$**	**Nb49**	570 ± 57		-7.0 ± 0.34		1:2
**APH$_2$**	**Nb28**	140 ± 10		-8.8 ± 0.33		1:2
**APH$_2$**	**Nb30**	710 ± 58		-6.1 ± 0.27		1:2
**Nb16**	**BCR$_2$**	30 ± 2.2		-22 ± 0.84		1:1
**Nb39***	**GCN$_2$**	850 ± 140	1700 ± 150	- 86 ± 8.9	63 ± 3.3	2:1
**Cooperative interactions between Nb28, Nb30 and tetrahedron TET12SN**						
**Cell**	**Syringe**	**Kd (nM)**		**ΔH (kcal/mol)**		**Stoichiometry (cell:syringe)**
**TET12SN**	**Nb28**	39 ± 2.8		-14 ± 0.49		1:2
**TET12SN**	**Nb30**	350 ± 35		-8.8 ± 0.42		1:2
**Nb30$_2$-TET12SN**	**Nb28**	2.3 ± 0.16		-15 ± 0.54		1:2
**Nb28$_2$-TET12SN**	**Nb30**	26 ± 1.8		-11 ± 0.41		1:2
**Cooperative interactions between Nb28, Nb30 and APH peptide pair**						
**Cell**	**Syringe**	**Kd (nM)**		**ΔH (kcal/mol)**		**Stoichiometry (cell:syringe)**
**APH$_2$**	**Nb28**	140 ± 10		-8.8 ± 0.33		1:2
**APH$_2$**	**Nb30**	710 ± 58		-6.1 ± 0.27		1:2
**Nb30$_2$-APH$_2$**	**Nb28**	23 ± 1.7		-12 ± 0.44		1:2
**Nb28$_2$-APH$_2$**	**Nb30**	55 ± 4.2		-7.3 ± 0.29		1:2
**Cooperative interactions between Nb28, Nb30 and tetrahedron TET12SN(22CC)**						
**Cell**	**Syringe**	**Kd (nM)**		**ΔH (kcal/mol)**		**Stoichiometry (cell:syringe)**
**TET12SN(22CC)**	**Nb28**	27 ± 1.9		-13 ± 0.46		1:2
**TET12SN(22CC)**	**Nb30**	270 ± 21		-6.7 ± 0.27		1:2
**Nb28$_2$-TET12SN(22CC)**	**Nb30**	24 ± 1.7		-12 ± 0.43		1:2
**Titrations at different temperatures**						
**Cell**	**Syringe**	**Kd (nM)**	**ΔH (kcal/mol)**		**ΔCp (kcal/molK)**	**Stoichiometry (cell:syringe)**
**APH$_2$**	**Nb28**	94 ± 7.5	-8.3 ± 0.30		-0.29 ± 0.012	1:2
**TET12SN**	**Nb28**	19 ± 1.8	-15 ± 0.47		-0.35 ± 0.014	1:2
**TET12SN**	**Nb30**	160 ± 12	-9.2 ± 0.35		-0.40 ± 0.017	1:2
**Nb30$_2$-TET12SN**	**Nb28**	1.6 ± 0.14	-16 ± 0.60		-0.25 ± 0.0099	1:2

Legend: *, Due to low affinity of the second binding site enthalpies could not be estimated reliably.

Table S3. Coiled-coil modules in tested CC protein origami cages

CC module	CC module (full name)	CC protein origami tetrahedron			CC protein origami prism	CC protein origami bipyramid	
		TET12SN[a]	TET12SN(22CC)[b]	TET12SN(222CC)[b]	TRIP18SN[c]	BIP18APH	BIP18SN[e]
APH_2	APHshSN-APHshSN	1	1	2	2[d]	2	0
BCR_2	BCRSN-BCRSN	1	1	0	1	0	2
GCN_2	GCNshSN-GCNshSN	1	0	0	1	0	0
P3-P4	P3SN-P4SN	1	2	2	1	2	2
P5-P6	P5SN-P6SN	1	2	2	1	1	1
P7-P8	P7SN-P8SN	1	0	0	0	1	1
P1-P2	P1SN-P2SN	0	0	0	1	2	2
P9-P10	P9SN-P10SN	0	0	0	1	1	1
P11-P12	P11SN-P12SN	0	0	0	1	0	0

Legend: [a], the same CC modules are also present also in TET12SN(W24A)$_1$, TET12SN(W24A)$_5$ and TET12SN(W24A)$_{1,5}$; [b], Reference 24 in the main text; [c], Reference 12 in the main text; [d], two different APH_2 modules (APHshSN-APHshSN and APH4SN-APH4SN); [e], Reference 31 in the main text; numbers 0, 1 or 2 in columns 3-8, repetitions of the CC module in the CC protein origami polyhedron.

SI References

1. C. Engler, R. Kandzia, S. Marillonnet, A one pot, one step, precision cloning method with high throughput capability. *PLoS One* **3**, e3647 (2008).
2. D. G. Gibson, L. Young, R. Y. Chuang, J. C. Venter, C. A. Hutchison, H. O. Smith, Enzymatic assembly of DNA molecules up to several hundred kilobases. *Nat. Methods* **6**, 343–345 (2009).
3. I. Drobnak, H. Gradišar, A. Ljubetič, E. Merljak, R. Jerala, Modulation of Coiled-Coil Dimer Stability through Surface Residues while Preserving Pairing Specificity. *J. Am. Chem. Soc.* **139**, 8229–8236 (2017).
4. C. E. Blanchet, A. Spilotros, F. Schwemmer, M. A. Graewert, A. Kikhney, C. M. Jeffries, D. Franke, D. Mark, R. Zengerle, F. Cipriani, S. Fiedler, M. Roessle, D. I. Svergun, Versatile sample environments and automation for biological solution X-ray scattering experiments at the P12 beamline (PETRA III, DESY). *J. Appl. Crystallogr.* **48**, 431–443 (2015).
5. K. Dyer, M. Hammel, R. Rambo, S. Tsutakawa, I. Rodic, S. Classen, J. Tainer, L. Hura, High-Throughput SAXS for the Characterization of Biomolecules in Solution: A Practical Approach. *Methods Mol Biol* **1091**, 245–258 (2014).
6. P. V. Konarev, V. V. Volkov, A. V. Sokolova, M. H. J. Koch, D. I. Svergun, IUCr, *PRIMUS* : a Windows PC-based system for small-angle scattering data analysis. *J. Appl. Crystallogr.* **36**, 1277–1282 (2003).
7. D. Franke, D. I. Svergun, DAMMIF, a program for rapid ab-initio shape determination in small-angle scattering. *J. Appl. Crystallogr.* **42**, 342–346 (2009).
8. S. Grudinin, M. Garkavenko, A. Kazennov, Pepsi-SAXS: an adaptive method for rapid and accurate computation of small-angle X-ray scattering profiles. *Acta Crystallogr. Sect. D, Struct. Biol.* **73**, 449–464 (2017).
9. P. Keller, Sandro; Vargas, Carolyn; Zhao, Huaying; Piszchek, Grzegorz; Brautigam, Chad; A. Schuck, High-Precision Isothermal Titration Calorimetry with Automated Peak Shape Analysis. *Anal. Chem.* **84**, 5066–5073 (2012).
10. T. H. Scheuermann, C. A. Brautigam, High-precision, automated integration of multiple isothermal titration calorimetric thermograms: New features of NITPIC. *Methods* **76**, 87–98 (2015).
11. H. Zhao, G. Piszczek, P. Schuck, SEDPHAT - A platform for global ITC analysis and global multi-method analysis of molecular interactions. *Methods* **76**, 137–148 (2015).
12. W. Kabsch, B. A. T., D. K., K. P. A., D. K., M. S., R. R. B. G., E. P., F. S., W. K., K. W., K. W., K. W., K. W., K. P., W. M. S., *XDS*. *Acta Crystallogr. Sect. D Biol. Crystallogr.* **66**, 125–132 (2010).
13. A. J. McCoy, R. W. Grosse-Kunstleve, P. D. Adams, M. D. Winn, L. C. Storoni, R. J. Read, Phaser crystallographic software. *J. Appl. Crystallogr.* **40**, 658–674 (2007).
14. B. Webb, A. Sali, Comparative Protein Structure Modeling Using MODELLER. *Curr. Protoc. Bioinformatics* **54**, 5.6.1–5.6.37 (2016).
15. C. W. Wood, J. W. Heal, A. R. Thomson, G. J. Bartlett, A. Ibarra, R. L. Brady, R. B. Sessions, D. N. Woolfson, ISAMBARD: An open-source computational environment for biomolecular analysis, modelling and design. *Bioinformatics* **33**, 3043–3050 (2017).
16. P. D. Adams, P. V. Afonine, G. Bunkóczi, V. B. Chen, I. W. Davis, N. Echols, J. J. Headd, L. W. Hung, G. J. Kapral, R. W. Grosse-Kunstleve, A. J. McCoy, N. W. Moriarty, R. Oeffner, R. J. Read, D. C. Richardson, J. S. Richardson, T. C. Terwilliger, P. H. Zwart, PHENIX: A comprehensive Python-based system for macromolecular structure solution. *Acta Crystallogr. Sect. D Biol. Crystallogr.* **66**, 213–221 (2010).
17. P. Emsley, B. Lohkamp, W. G. Scott, K. Cowtan, Features and development of Coot. *Acta Crystallogr. Sect. D Biol. Crystallogr.* **66**, 486–501 (2010).
18. S. J. Hubbard, J. M. Thornton, ´NACCESS´, Computer Program. Department of Biochemistry and Molecular Biology, University College, London (1993).
19. E. Krissinel, K. Henrick, Inference of Macromolecular Assemblies from Crystalline State. *J. Mol. Biol.* **372**, 774–797 (2007).
20. T. Kortemme, D. Baker, A simple physical model for binding energy hot spots in protein-protein complexes. *Proc. Natl. Acad. Sci. U. S. A.* **99**, 14116–14121 (2002).
21. J. Schymkowitz, J. Borg, F. Stricher, R. Nys, F. Rousseau, L. Serrano, The FoldX web server: An online force field. *Nucleic Acids Res.* **33**, 382–388 (2005).

22. F. H. C. Crick, The Fourier transform of a coiled-coil. *Acta Crystallogr.* **6**, 685–689 (1953).
23. S. McIntosh-Smith, J. Price, R. B. Sessions, A. A. Ibarra, High performance in silico virtual drug screening on many-core processors. *Int. J. High Perform. Comput. Appl.* **29**, 119–134 (2015).
24. G. Krivov, M. Shapovalov, R. Dunbrack Jr., Improved prediction of protein side-chain conforma-tions with SCWRL4. *Proteins* **77**, 778–795 (2009).
