# Supplementary figures and images for: A nanobody toolbox targeting dimeric coiled-coil modules for functionalization of designed protein origami structures
Source: Proc Natl Acad Sci U S A. 2021 Apr 23;118(17):e2021899118. doi: 10.1073/pnas.2021899118 (PMC8092592; doi:10.1073/pnas.2021899118)

# Uncropped scan of the native PAGE gel for Fig. 1

Fig. 1B\*

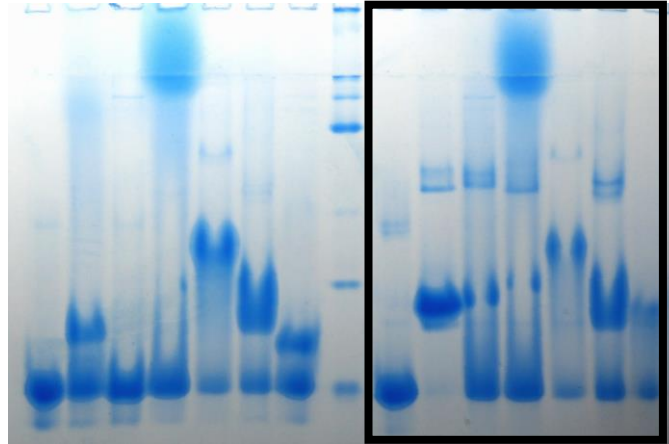

\*The same scan was used also for Fig. S8A.

Supplement: Supplementary File [file pnas.2021899118.sd01.pdf]

# Uncropped scans of the native PAGE gels from Fig. S16\*

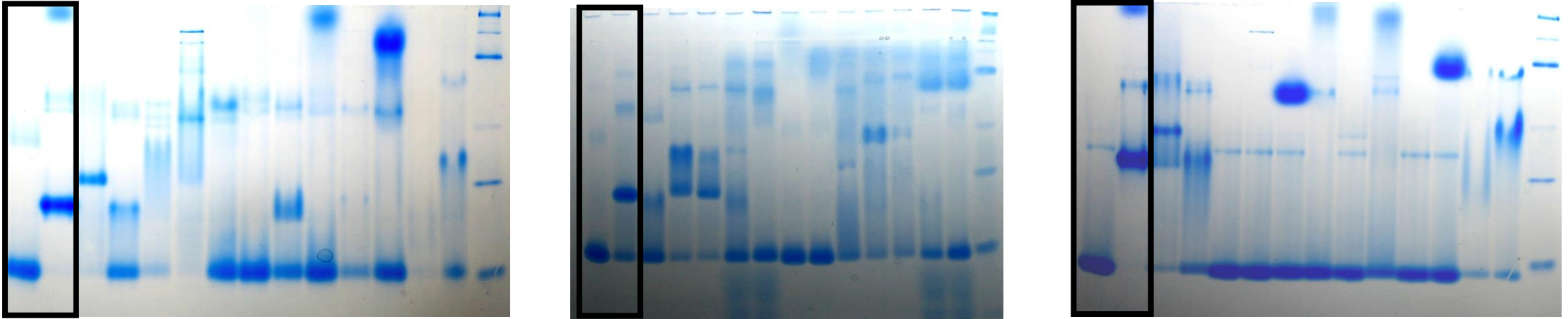

\*The same scans were used also for Fig. 7.

Supplement: Supplementary File [file pnas.2021899118.sd06.pdf]

# Uncropped scan of the native PAGE gel from Fig. S18

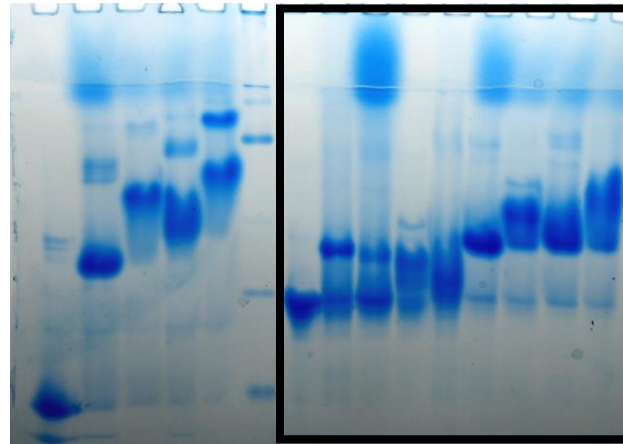

Supplement: Supplementary File [file pnas.2021899118.sd07.pdf]

# Uncropped scans of the native PAGE gels from Fig. S19

Nb26, Nb49

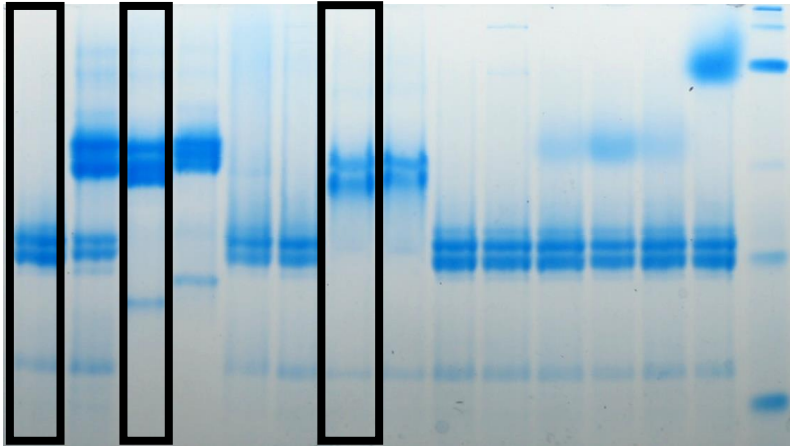

Nb28, Nb39, Nb30, Nb28 + Nb30

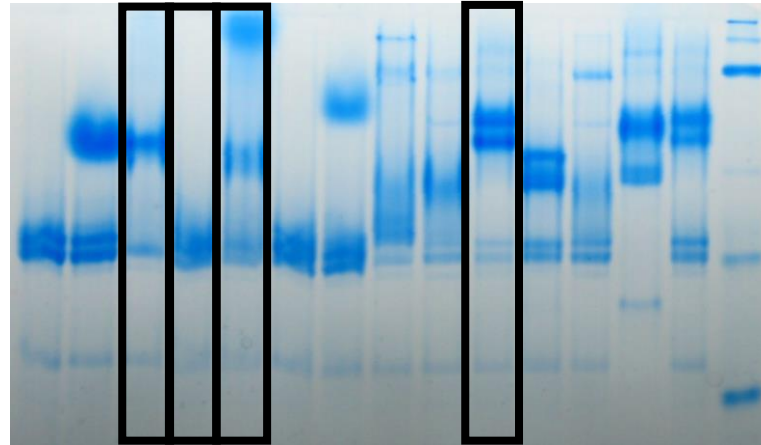

Nb34, Nb26 + Nb34

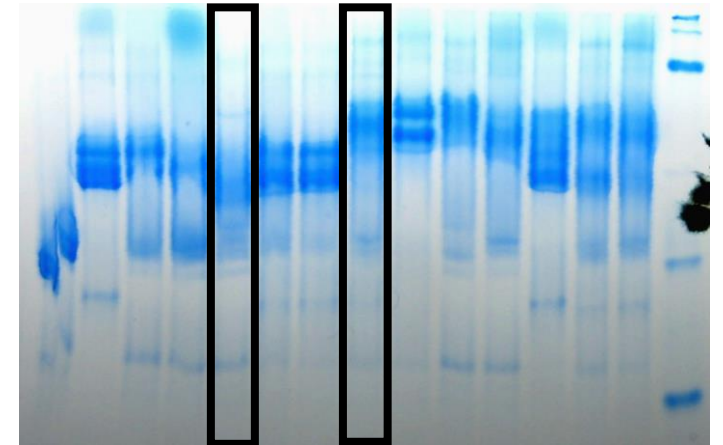

Supplement: Supplementary File [file pnas.2021899118.sd08.pdf]

## Uncropped scan of the native PAGE gel from Fig. S20

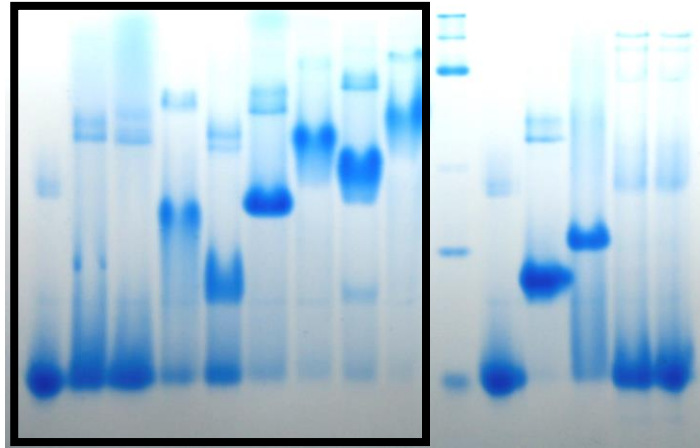

Supplement: Supplementary File [file pnas.2021899118.sd09.pdf]
