# Supplementary material for: A nanobody toolbox targeting dimeric coiled-coil modules for functionalization of designed protein origami structures
Source: Proc Natl Acad Sci U S A. 2021 Apr 23;118(17):e2021899118. doi: 10.1073/pnas.2021899118 (PMC8092592; doi:10.1073/pnas.2021899118)

Uncropped scans of the native PAGE gels from Fig. 7

TET12SN + Nb26*

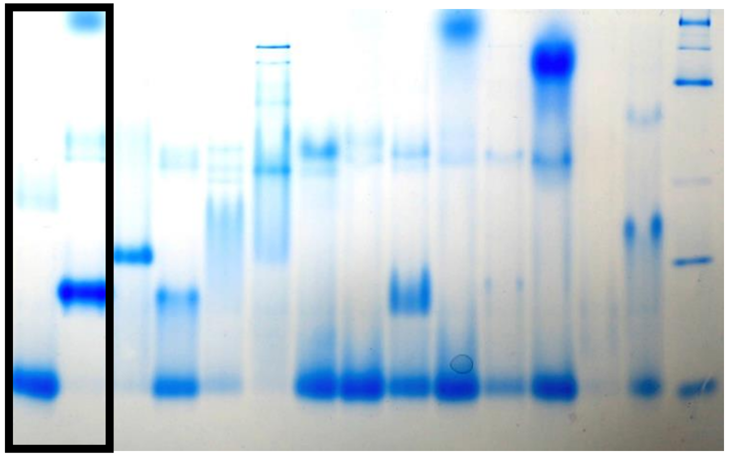

TET12SN(22CC) + Nb26*

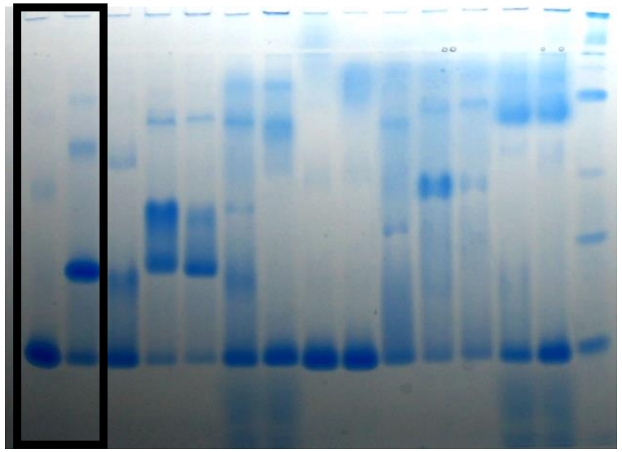

TET12SN(222CC) + Nb26*

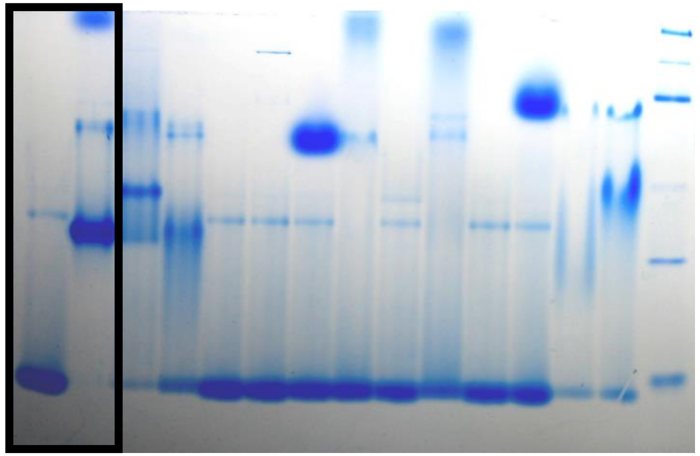

*The same scans were used also for Fig. S16.

TRIP18SN + Nb26

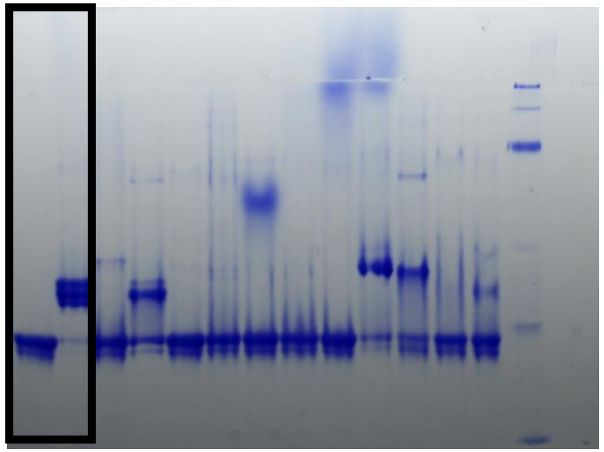

BIP18APH + Nb26

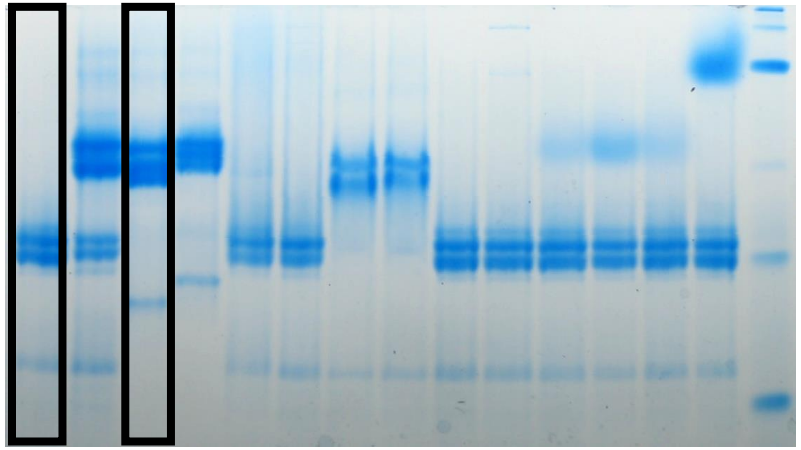

TET12SN + Nb16

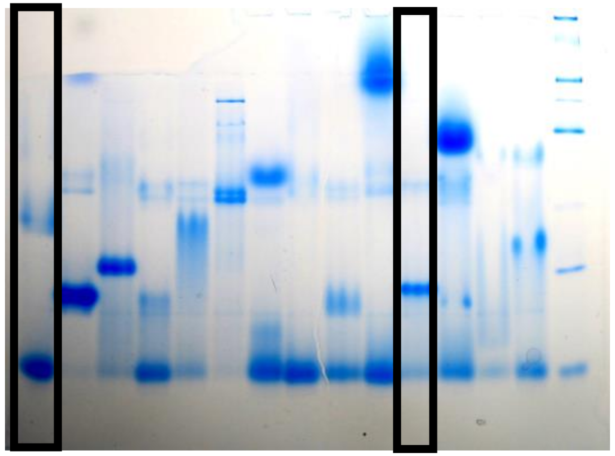

TRIP18SN + Nb16

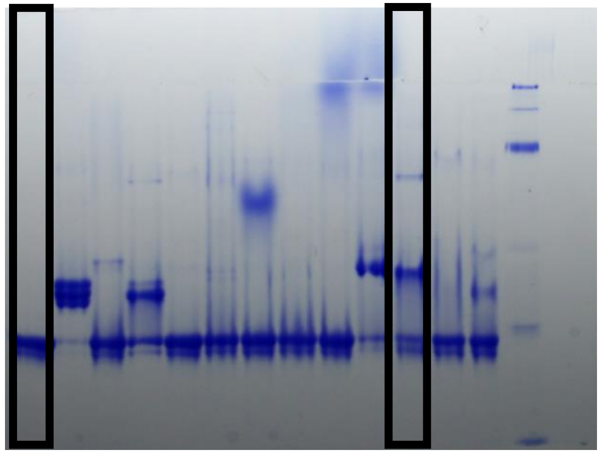

BIP18SN

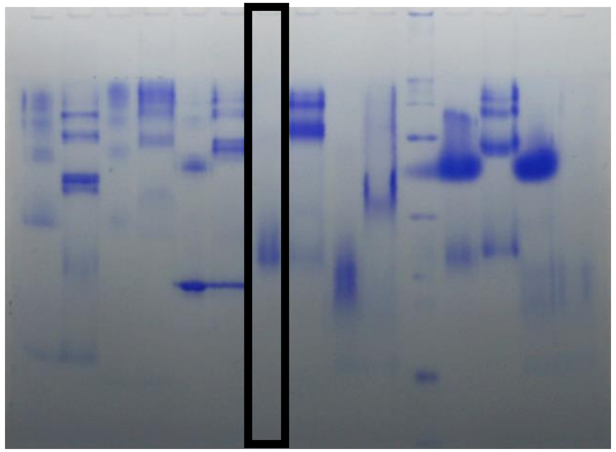

BIP18SN + Nb16

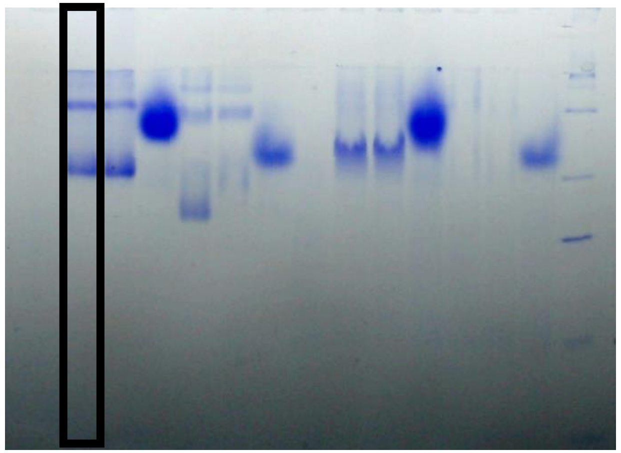

TET12SN + Nb39

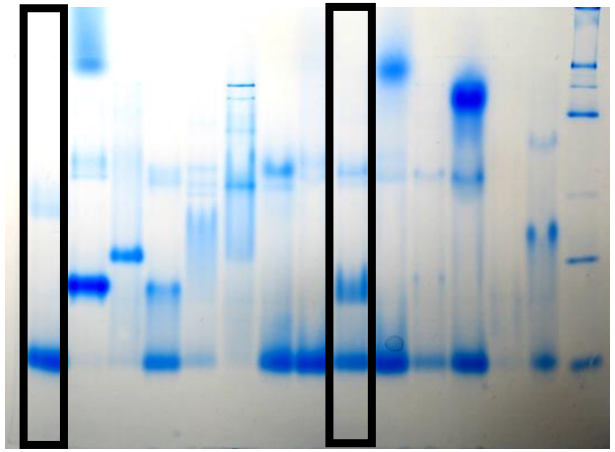

TRIP18SN + Nb39

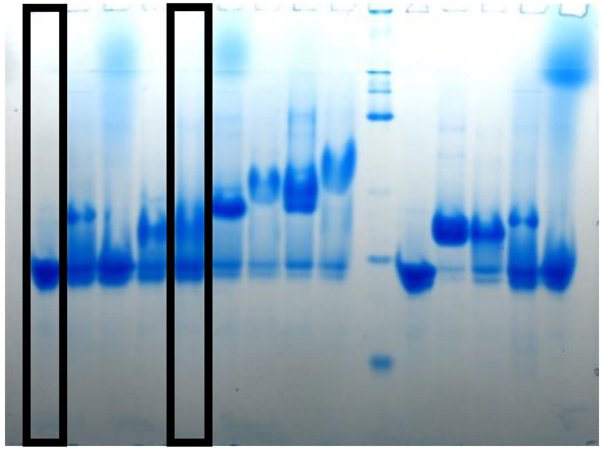

Supplement: Supplementary File [file pnas.2021899118.sd02.pdf]
