# Supplementary material for: A nanobody toolbox targeting dimeric coiled-coil modules for functionalization of designed protein origami structures
Source: Proc Natl Acad Sci U S A. 2021 Apr 23;118(17):e2021899118. doi: 10.1073/pnas.2021899118 (PMC8092592; doi:10.1073/pnas.2021899118)

Uncropped scans of the native PAGE gels from Fig. S2

Uncropped scans of the native PAGE gels from Fig. *S2A*

(Nb26, Nb15, Nb64, Nb49, Nb21, Nb59, Nb54, Nb28, Nb39, Nb30, Nb16, Nb5, Nb17, Nb34)

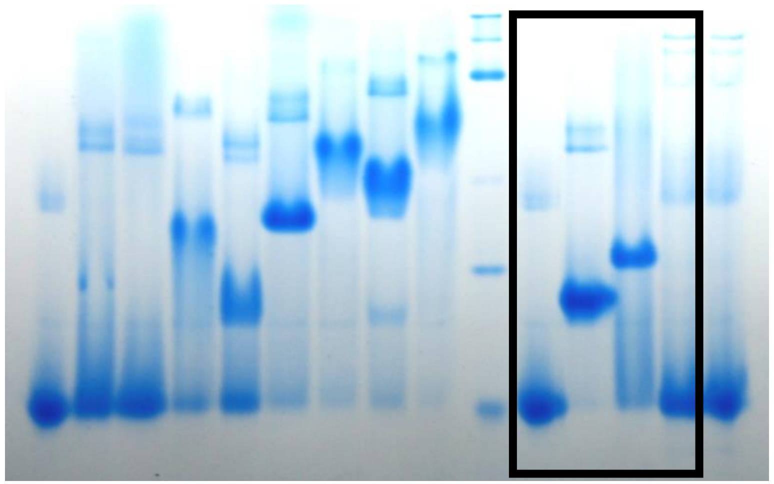

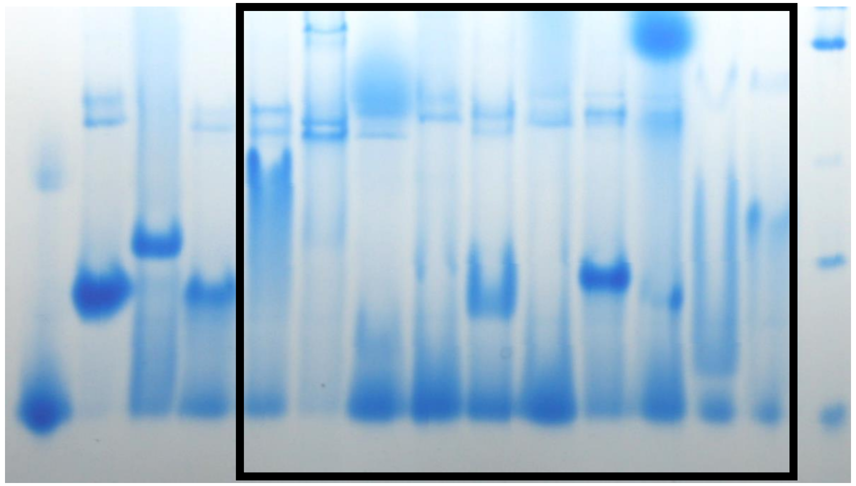

Uncropped scans of the native PAGE gels from Fig. *S2B*

Nb10

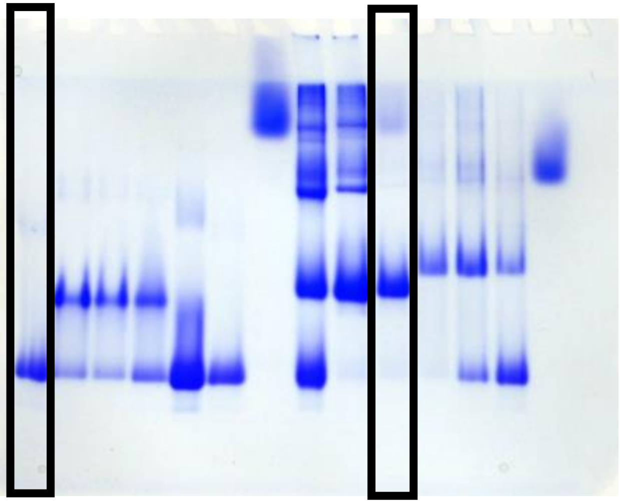

Nb13

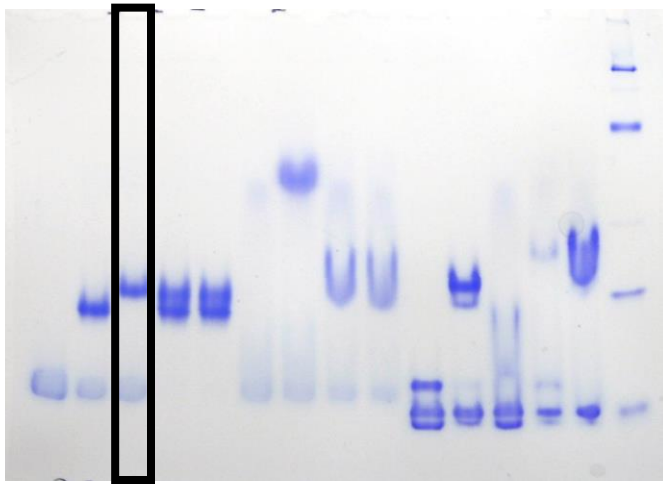

Nb66

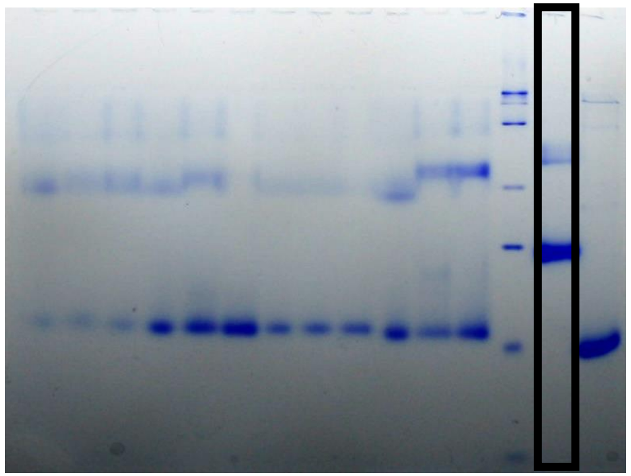

Nb94

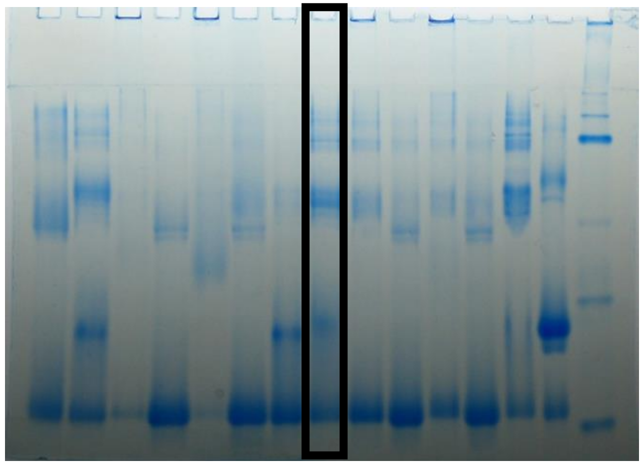

Nb1

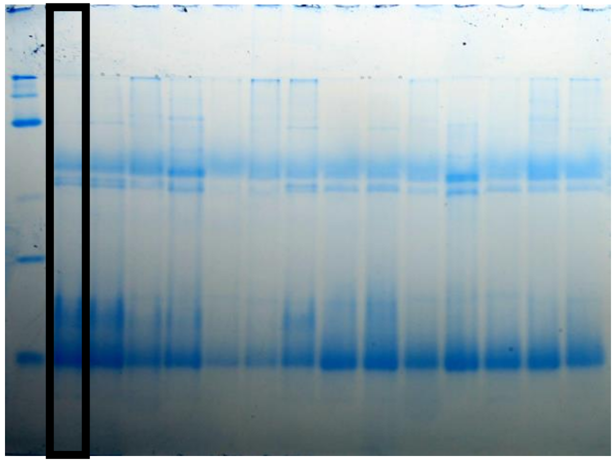

Nb48

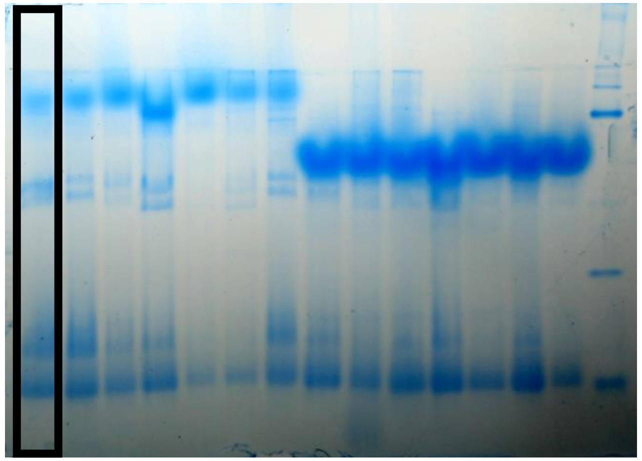

Uncropped scans of the native PAGE gel from Fig. S2*C*

Nb28

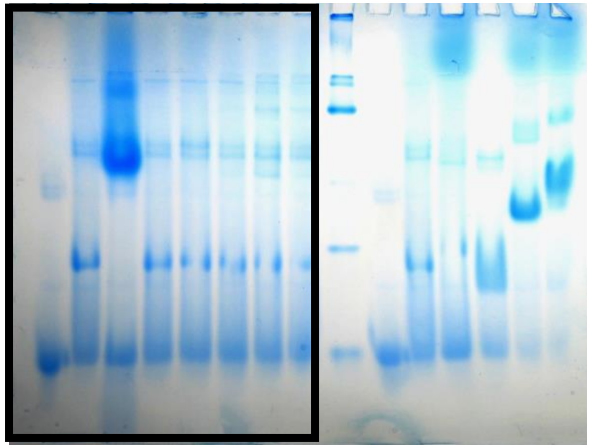

Nb30

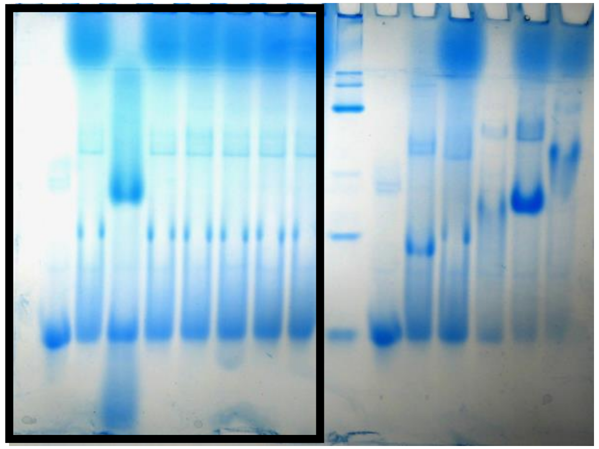

Nb26

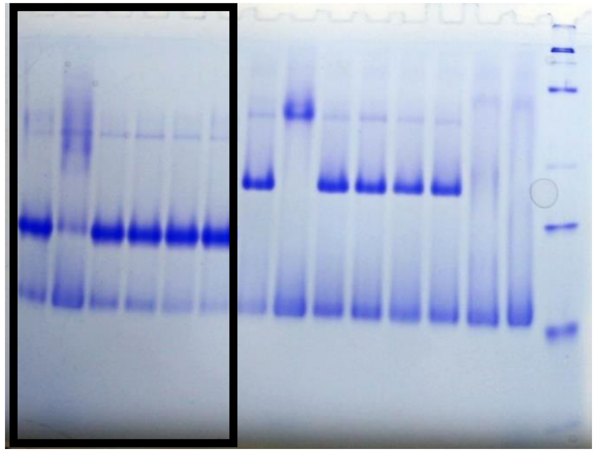

Nb26

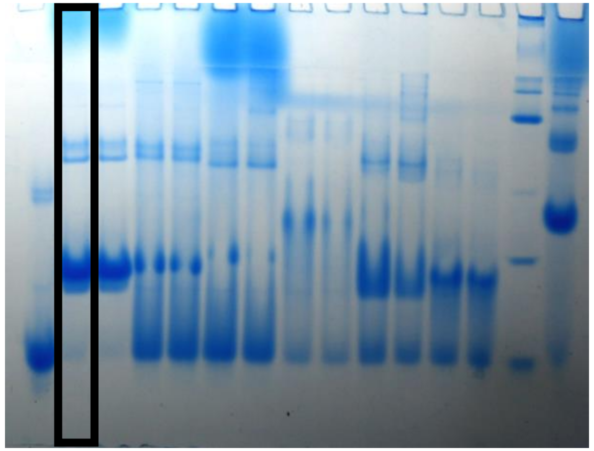

Nb49

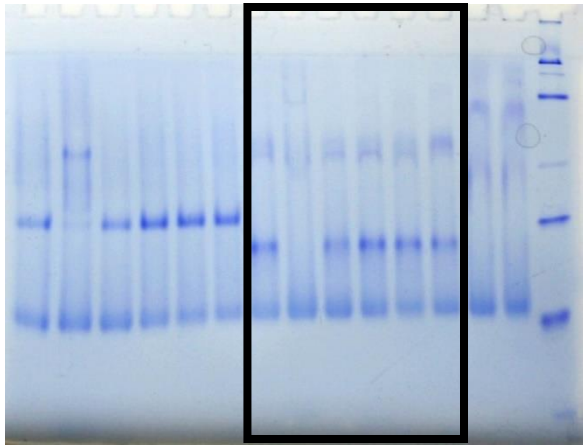

Nb49

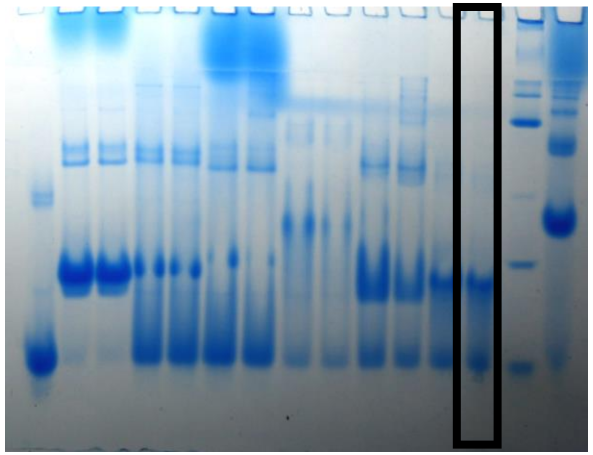

Nb16

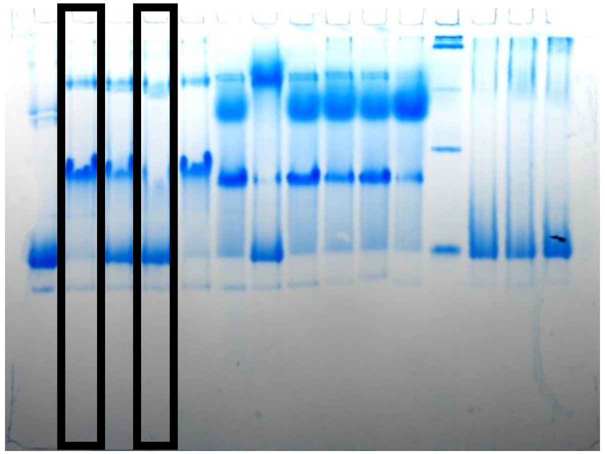

Nb16

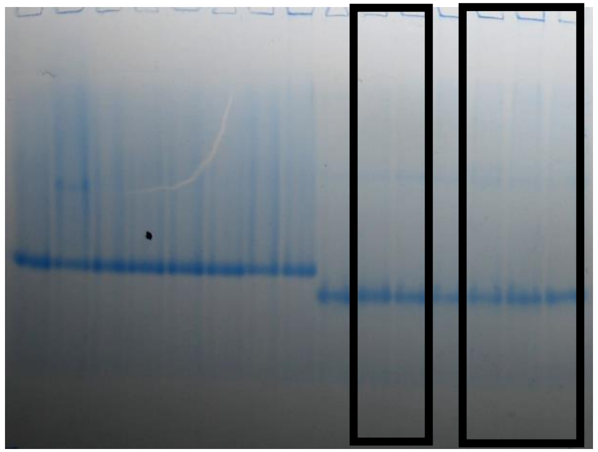

Uncropped scans of the native PAGE gel from Fig. *S2C*

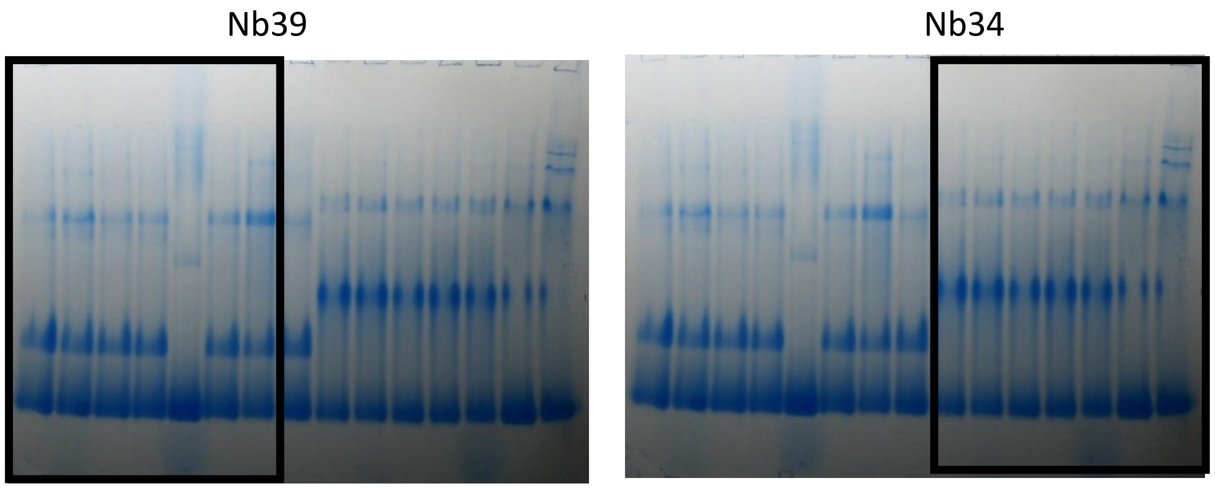

Uncropped scans of the native PAGE gels from Fig. *S2D*

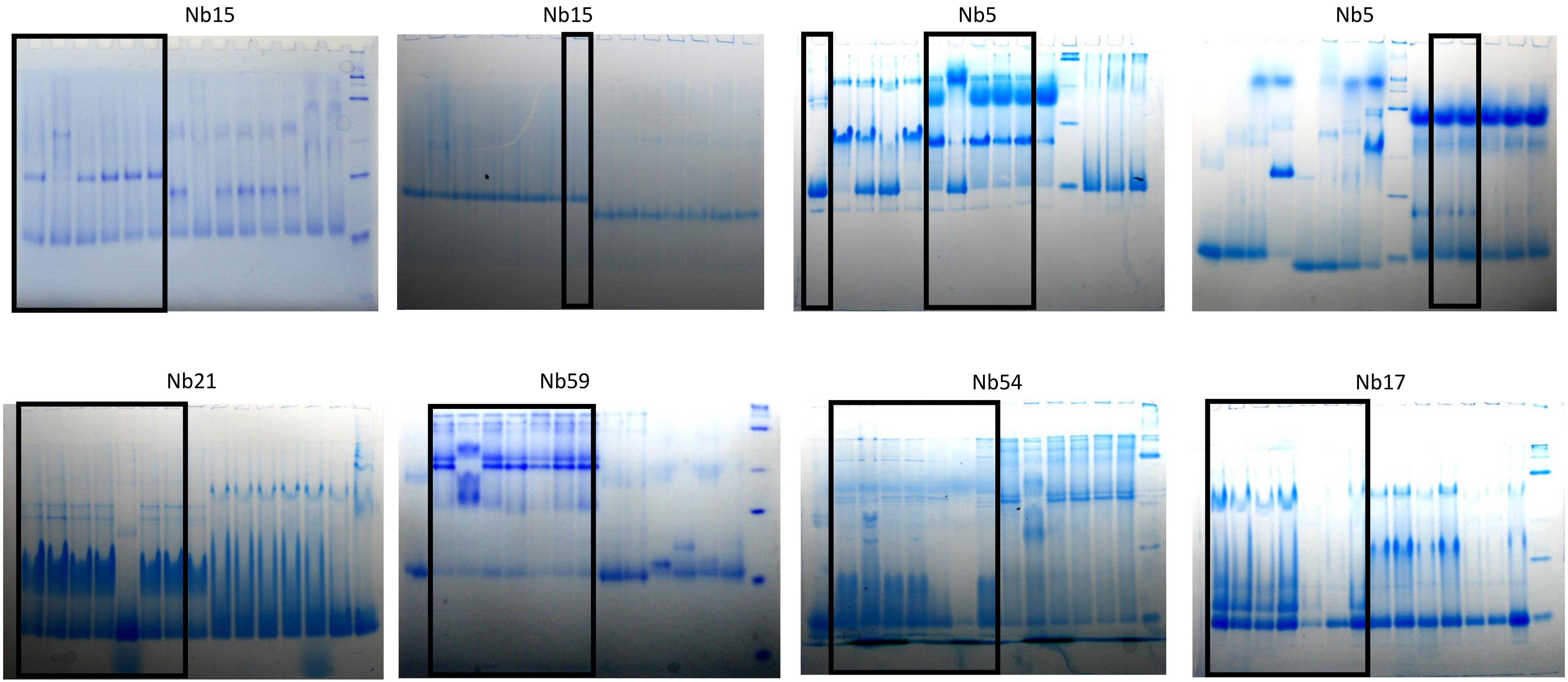

Supplement: Supplementary File [file pnas.2021899118.sd03.pdf]
