# Supplementary material for: A nanobody toolbox targeting dimeric coiled-coil modules for functionalization of designed protein origami structures
Source: Proc Natl Acad Sci U S A. 2021 Apr 23;118(17):e2021899118. doi: 10.1073/pnas.2021899118 (PMC8092592; doi:10.1073/pnas.2021899118)

Uncropped scans of the native PAGE gels for Fig. S8

Fig. S8*A**

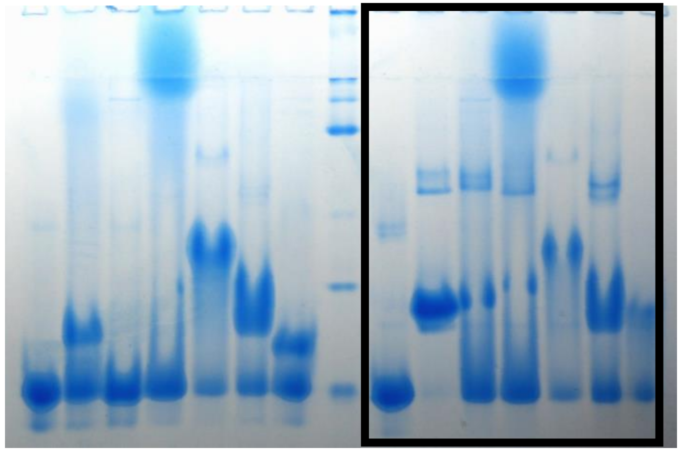

*The same scan was used also for Fig. 1*B.*

Fig. S8*B*

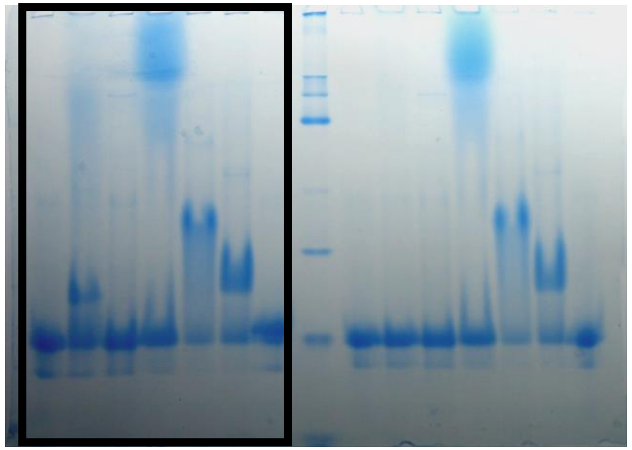

Fig. S8*C*

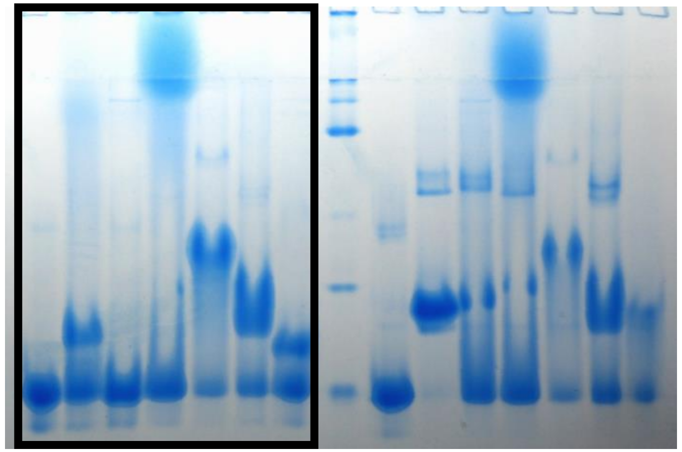

Fig. S8*D*

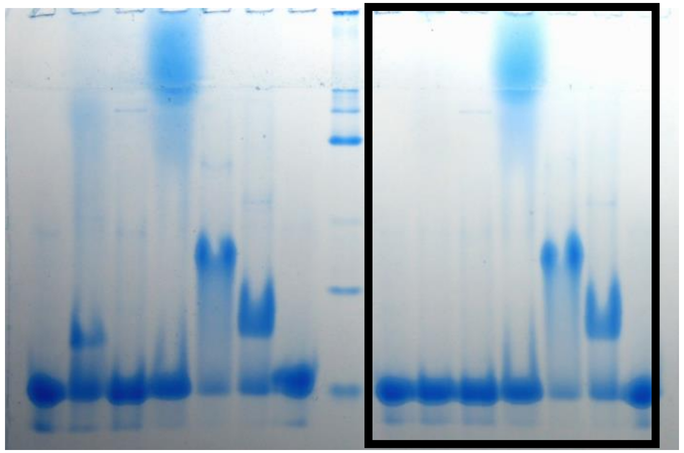

Fig. S8*E*

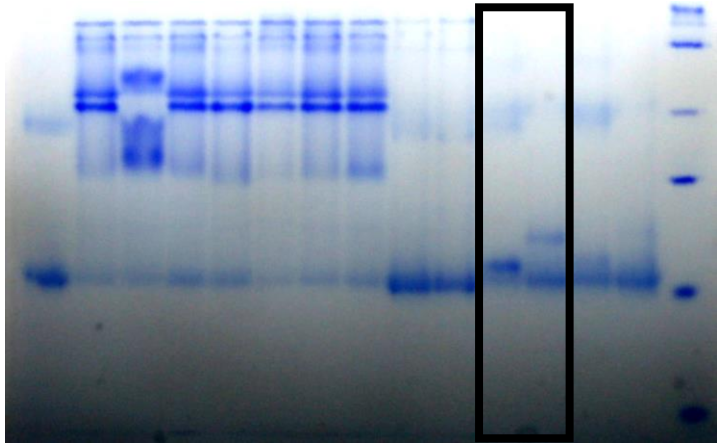

Supplement: Supplementary File [file pnas.2021899118.sd04.pdf]
