# Supplementary material for: A nanobody toolbox targeting dimeric coiled-coil modules for functionalization of designed protein origami structures
Source: Proc Natl Acad Sci U S A. 2021 Apr 23;118(17):e2021899118. doi: 10.1073/pnas.2021899118 (PMC8092592; doi:10.1073/pnas.2021899118)

Uncropped scans of the native PAGE gels from Fig. S9

Uncropped scans of the native PAGE gels from Fig. *S9A*

Nb26, Nb49, Nb28, Nb30

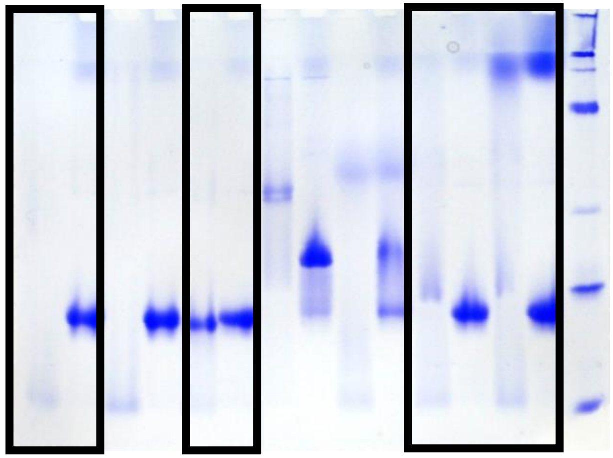

Nb49, Nb28, Nb30

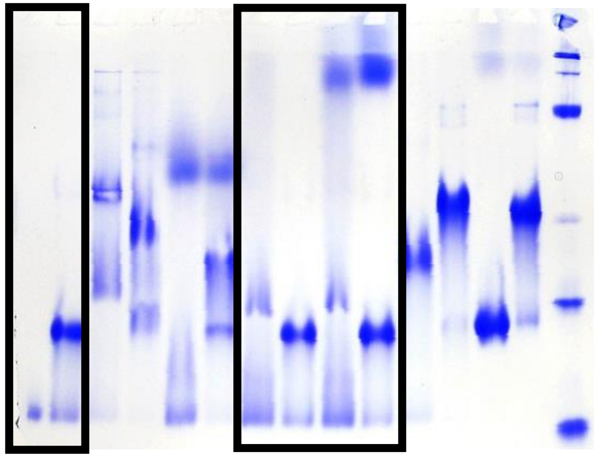

Nb28, Nb30

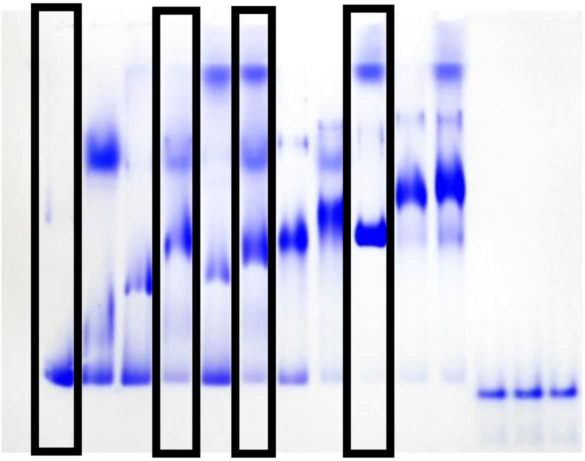

Nb26, Nb28, Nb30

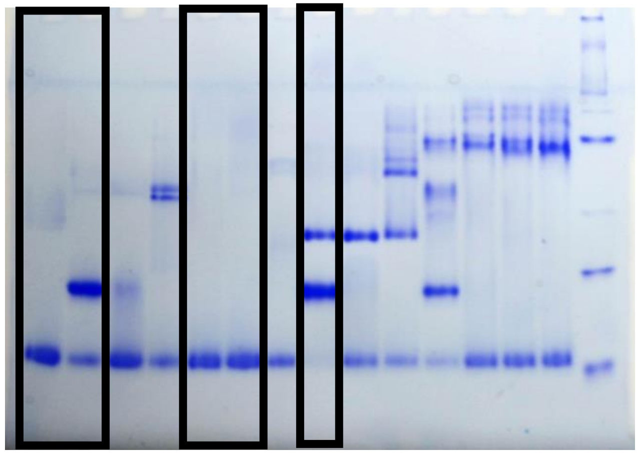

Uncropped scan of the native PAGE gel from Fig. S9*B*

Nb26, Nb13

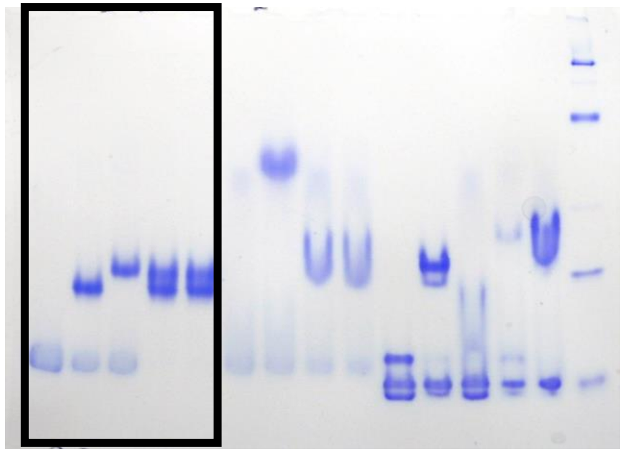

Uncropped scan of the native PAGE gel from Fig. S9*C*

Nb26, Nb15

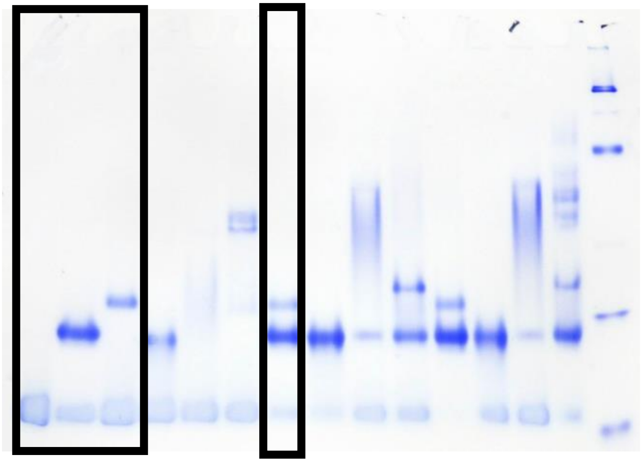

Uncropped scans of the native PAGE gel from Fig. S9*D*

Nb28, Nb30

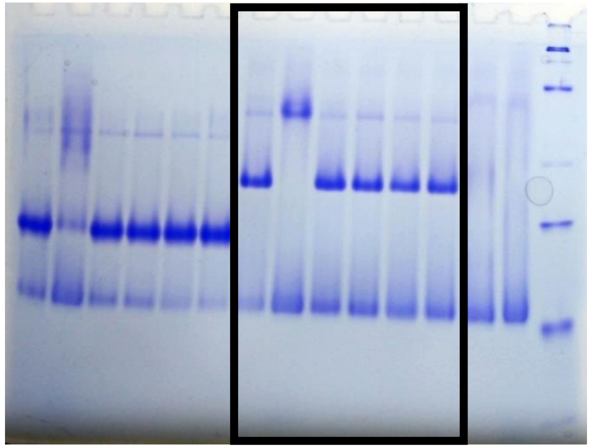

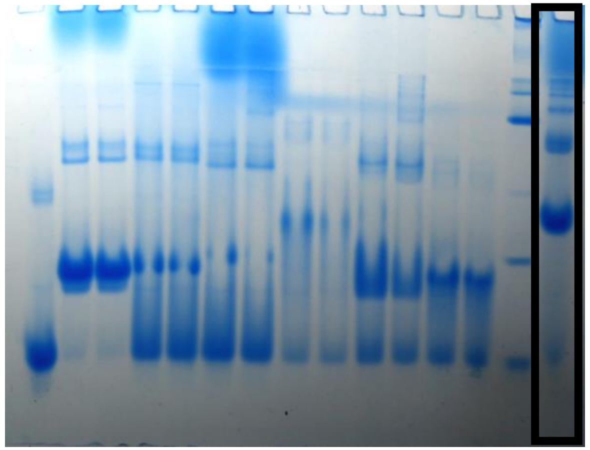

Supplement: Supplementary File [file pnas.2021899118.sd05.pdf]
